# Supplementary material for: Biosynthetic energy cost for amino acids decreases in cancer evolution
Source: Nat Commun. 2018 Oct 8;9:4124. doi: 10.1038/s41467-018-06461-1 (PMC6175916; doi:10.1038/s41467-018-06461-1)
Supplement: Supplementary file 1 — Supplementary Information [file 41467_2018_6461_MOESM1_ESM.pdf]

Biosynthetic Energy Cost for Amino Acids Decreases in Cancer Evolution

Zhang et al.

Supplementary Information

Table of Contents

Supplementary Methods 2

Supplementary Tables... 7

Supplementary Figures 20

Supplementary References.. 58

Supplementary Methods

Calculation of biosynthetic cost of amino acids in human

Amino acids (AAs) are synthesized from intermediate metabolites of glycolysis, pentose phosphate pathway and tricarboxylic (TCA) cycle. The cost of synthesizing an AA is composed of two parts: the energy would have been produced if the precursor molecule was not diverted from energy metabolism and the energy required to convert the precursor molecule to the desired AA through series enzymatic reactions[1-4]. The energetic cost is calculated in terms of high energy phosphate bonds (~P)[2].

In order to calculate the biosynthetic cost of non-essential AAs (NEAAs) in human, we took into consideration the differences in AA biosynthesis pathways between human[5] and autotrophs and calculated the cost of each AA following the procedures that were previously performed in bacteria[1,2] and yeast[3,4]. Note cysteine is synthesized from serine and homocysteine that is produced during catabolism of methionine. Tyrosine is derived by hydroxylation of phenylalanine. Since both methionine and phenylalanine are essential AAs (EAAs), the biosynthetic cost in the autotrophs was not considered here. We only calculated the cost consumed in converting homocysteine to cysteine, and the cost of converting phenylalanine to tyrosine. We assume the availability of ammonium and homocysteine as non-limiting resources of nitrogen and sulfide, similar to previous studies[1-4].

Differences in pathways of AA biosynthesis between human and autotrophs: For most of the NEAAs, the biosynthetic steps from precursor molecules to the final amino acids are well conserved among species from bacteria and fungi to plants and animals. However, there are several differences in amino acid biosynthesis that make animals different from bacteria and plants[5]:

- Cysteine and tyrosine are synthesized from EAA methionine and phenylalanine, respectively. Therefore, these two amino acids cannot be synthesized *de novo* in animals.
- In bacteria and plants, Glutamate synthase catalyzes the reaction converting α-ketoglutarate to glutamate, which consumes 1 ATP. But this enzyme is absent in most animals[6]. Instead, animals convert α-ketoglutarate to glutamate with transamination reactions, which doesn't consume ATP.
- Arginine is synthesized from glutamate and acetyl-CoA in plants and bacteria. However, several enzymes required for these reactions leading to ornithine are absent in animals. Instead, animals utilize ornithine produced in urea cycle from Δ^1 -Pyrroline-5-carboxylate (P5C) through the reversible reaction catalyzed by ornithine Δ -aminotransferase [7]. Therefore, the biosynthesis cost of arginine is different for animals.
- When oxaloacetate is used as the precursor for AA biosynthesis, the oxaloacetate is assumed to be derived from phosphoenolpyruvate (PEP) without entering into TCA cycle[1]. However, the enzyme required for carboxylation of PEP to oxaloacetate (PEP carboxylase) is found only in plants and bacteria but never in fungi and animals [8]. In animals, we assumed the oxaloacetate

is synthesized from pyruvate by pyruvate carboxylase through gluconeogenesis pathway, which consumes 1 pyruvate and 1 ATP.

- In order to synthesize cysteine, 3-phosphoglycerate is first to convert to serine. In bacteria and plants, the reduced sulfide is derived from sulfate through reduction reactions catalyzed by phosphoadenylyl-sulfate reductase and sulfide reductase. In animals, the sulfide is contributed by homocysteine, which is produced during catabolism of methionine[9].

Cost of precursors for AA biosynthesis: In animals, the 11 NEAAs are synthesized from several common precursors. glutamate, glutamine, proline and arginine are derived from α-ketoglutarate. Serine, glycine and cysteine are derived from 3-phosphoglycerate. Aspartate and asparagine are derived from oxaloacetate. Alanine is derived from pyruvate. Tyrosine is derived from phenylalanine in animals (from phosphoenolpyruvate and erythrose in bacteria and plants).

The energy sacrificed by diverting precursor molecules from energy metabolism is summarized below (see also Supplementary Fig. 1):

Precursor	ATP	$FADH_2$	NAD(P)H	Total cost (~P)
3-phosphoglycerate	2	1	4	13.5
pyruvate	1	1	4	12.5
α-ketoglutarate	2	1	2	8.5
oxaloacetate	2	1	4	13.5
phenylalanine	0	0	0	0

For precursor **pyruvate**, 1 NADH is produced when being converted to acetyl-CoA before entering TCA cycle. 1 ATP, 3 NADH and 1 $FADH_2$ are generated in the TCA cycle. Therefore, the precursor cost for pyruvate would be 1 ATP + 4 NADH + 1 $FADH_2$.

For precursor **$\boldsymbol{\alpha}$-ketoglutarate**, 1 GTP, 2 NADH and 1 $FADH_2$ are produced when α-ketoglutarate is converted to oxaloacetate. Another ATP is consumed to produce the oxaloacetate required to replenish the TCA cycle by converting pyruvate to oxaloacetate through pyruvate carboxylase. Therefore, the total precursor cost of α-ketoglutarate would be 2 NADH + 1 $FADH_2$.

For **3-phosphoglycerate**, 1 ATP is produced when 3-phosphoglycerate is converted to pyruvate and the precursor cost of pyruvate is 1 ATP + 4 NADH + 1 $FADH_2$ as deduced above. Therefore, the precursor cost of 3-phosphoglycerate would be 2 ATP + 4 NADH + 1 $FADH_2$.

For **oxaloacetate**, we assume that it is synthesized from pyruvate through gluconeogenesis pathway from pyruvate. Converting pyruvate to oxaloacetate consumes 1 ATP and 1 ATP + 4 NADH + 1 $FADH_2$ would not be produced from pyruvate through energy metabolism. Therefore, the precursor cost for oxaloacetate would be 2 ATP + 4 NADH + 1 $FADH_2$.

Phenylalanine is an EAA derived from food intake or protein turnover. It cannot be synthesized *de novo* in human so that its cost is not considered in our calculation.

Biosynthetic cost of NEAAs in human (animals): The energy cost during synthesis of amino acids from precursor molecules is listed below:

Amino acid	Precursor	ATP	$FADH_2$	NAD(P)H	Total cost (~P)
Alanine	pyruvate	0	0	0	0
Arginine	α-ketoglutarate	6	0	0	6
Asparagine	oxaloacetate	3	0	0	3
Aspartate	oxaloacetate	0	0	0	0
Cysteine	3-phosphoglycerate	0	0	-1	-2.5
Glutamate	α-ketoglutarate	0	0	0	0
Glutamine	α-ketoglutarate	1	0	0	1
Glycine	3-phosphoglycerate	0	0	-1	-2.5
Proline	α-ketoglutarate	1	0	2	6
Serine	3-phosphoglycerate	0	0	-1	-2.5
Tyrosine	phenylalanine	0	0	1	2.5

Glutamate: Glutamate is derived from α-ketoglutarate through transamination during catabolism of amino acids, which doesn't consume ATP (Supplementary Fig. 2a).

Glutamine: Glutamine is synthesized from glutamate by glutamine synthetase through a two-step reaction (Supplementary Fig. 2b):

1) glutamate + ATP -> γ-glutamyl phosphate + ADP
2) γ-glutamyl phosphate + NH_4^+ -> glutamine + P_i + H^+

Therefore, converting α-ketoglutarate to glutamine consumes 1 ATP.

Proline: Proline is derived from α -ketoglutarate, which is convert to glutamate first (Supplementary Fig. 2c). Glutamate is converted to γ-glutamylphosphate by glutamate kinase, which consumes 1 ATP. The acyl phosphate is then reduced to glutamate γ-semialdehyde by consuming 1 NADH. Glutamate γ-semialdehyde spontaneous cyclizes to form P5C. Proline is derived by reducing P5C by pyrroline carboxylate reductase, which consumes 1 NADPH. Therefore, 1 ATP + 2 NADH are consumed in reactions of proline biosynthesis.

Arginine: In animals, arginine is synthesized from ornithine (Supplementary Fig. 2d)[7]. Ornithine itself is synthesized from P5C by reverse reaction catalyzed by ornithine Δ-aminotransferase, while biosynthesis of P5C from α-ketoglutarate consumes 1 ATP and 1 NADH as we described above in proline biosynthesis. Next a carbamoyl group is transferred to ornithine from carbamoyl phosphate to form citrulline without consuming ATP. Carbamoyl phosphate is synthesized in the mitochondria matrix of liver from bicarbonate and ammonia, where the ammonia is transformed into mitochondria in the form glutamine as free ammonia is toxic to animal tissues. Conversion of free ammonia to glutamine by glutamine synthetase requires 1 ATP and derivation of carbamoyl phosphate from bicarbonate and

ammonia requires 2 ATP. In total, biosynthesis of carbamoyl phosphate requires 3 ATP. Next, argininosuccinate synthetase converts 1 molecule of citrulline and 1 molecule to 1 molecule of argininsuccinate, during which one ATP is converted to AMP (2 ~P). Finally, argininsuccinate is decomposed to produce arginine and fumarate. Recycling fumarate to aspartate will produce 1 NADH. Therefore, the net cost of biosynthetic steps is 6 ~P.

Serine: In order to synthesize serine, the hydroxyl group of 3-phosphoglycerate is oxidized to produce 3-phosphohydropyruvate and a NADH is produced. 3-phosphohydropyruvate gains an amino group to form 3-phosphoserine by transamination from glutamate. The phosphate group is then hydrolyzed from 3-phosphoserine to form serine (Supplementary Fig. 3a). In total, one NADH is produced during biosynthesis of serine.

Glycine: Serine is converted to glycine by serine-hydroxymethyl-transferase with tetrahydropholic acid as cofactor (Supplementary Fig. 3b). Therefore, the biosynthetic cost of glycine is the same as serine.

Cysteine: In order to synthesize cysteine, 3-phosphoglycerate is converted to serine first. In bacteria and plants, the sulfide group is derived from reduction of sulfate. In animals, the sulfide group is contributed by homocysteine, which is derived from catabolism of methionine. Serine and homocysteine is then converted to α -ketobutyrate and cysteine without consuming ATP (Supplementary Fig. 3c). As methionine is an EAA, we will only count ATP and NAD(P)H consumed during conversion of 3-phosphoglycerate to serine (-1 NADH) and during conversion of serine and homocysteine to cysteine (0). Therefore, the cost in biosynthetic steps of cysteine is the same as serine in our calculation.

Aspartate: Similar to glutamate, aspartate is derived through transamination without consuming ATP but to oxaloacetate (Supplementary Fig. 3d).

Asparagine: In order to synthesize asparagine, oxaloacetate is first converted to aspartate. Aspartate is converted to asparagine with glutamine donating an amino group (Supplementary Fig. 3e). During this reaction, a ATP is convert to AMP (2 ~P). Recycling glutamate to glutamine consumes 1 ATP, therefore, 3 ~P are consumed during biosynthesis of asparagine.

Alanine: Similar to glutamate and aspartate, alanine is derived by transamination from glutamate to pyruvate without consuming ATP (Supplementary Fig. 3f).

Tyrosine: Tyrosine is derived from phenylalanine by hydroxylation reaction and 1 NADH is consumed in this reaction (Supplementary Fig. 3g).

Conversion between different energy currencies: The cellular energy is stored in the form of high energy phosphate bond in ATP and electron carriers NAD(P) and $FADH_2$. During oxidative phosphorylation, 1.5 molecules of ATP are generated for each $FADH_2$ and 2.5 molecules of ATP are generated for each NAD(P)H on average. Following previous conventions, we calculate the total cost of amino acid biosynthesis by converting the number of $FADH_2$ and NAD(P)H consumed to number of high energy phosphate bonds (~P) with the relationship 1 $FADH_2$ = 1.5 ~P and 1 NAD(P)H = 2.5

~P. For hydroxylation of ATP to AMP, two high energy phosphate bonds are broken and counted as 2 ~P.

Decay-rate normalized cost (H11) is derived by normalizing the biosynthetic cost of NEAAs in human (h11) with their decay rate[10] as described in methods and the decay rate normalized cost is used throughout the main analysis. The h11 metric can be found in Supplementary Table 1 and was also presented in Supplementary Fig. 2 and 3. The H11 metric can be found in Supplementary Table 1.

Correlation between cost of 20 AAs in autotrophs and their relative abundance in the simulated free AA pool of human cells

Human intracellular free AAs are from two sources: 1) NEAAs synthesized endogenously in human cells or other heterotrophs and 2) AAs ultimately taken from autotrophs. In order to simulate the free AA pool in human cells, we assumed that AAs from source 2 are in proportion to their relative abundance the proteomes of yeast[11] or bacteria[12] and that the relative abundance of NEAAs from source 1 is the same as their abundance in human serum[13]. Since 3 and 2 amino acids in yeast and bacteria, respectively, were not quantified in those studies, we imputed their abundance from their cost in the corresponding species based on linear regression of $\log_2$(abundance of AAs) against the cost of AAs (Y20 for yeast and B20 for bacteria) using the quantified AAs. If p proportion of free AAs is from source 1, the remaining 1-p proportion of AAs will be from source 2. Then we performed correlation analysis between cost of AAs (Y20 for yeast and B20 for bacteria) and their abundance ($\log_2$ transformed) in the simulated free AA pool for each p from 0.01 to 0.9 with a step size of 0.01.

Supplementary Tables

Supplementary Table 1. Biosynthetic cost of amino acids in bacteria (B20), yeasts (Y20), and human (H11) and rank-based decay rate.

Amino acids	abbreviation	Decay rate*	b20	y20	h11	B20	Y20	H11
NEAAs								
Alanine	Ala, A	1	11.7	14.5	12.5	11.7	14.5	12.5
Arginine	Arg, R	4	27.3	20.5	14.5	109.2	82.0	58.0
Asparagine	Asn, N	10	14.7	18.5	16.5	147.0	185.0	165.0
Aspartate	Asp, D	9	12.7	15.5	13.5	114.3	139.5	121.5
Cysteine	Cys, C	30	24.7	26.5	11.0	741.0	795.0	330.0
Glutamate	Glu, E	5	15.3	9.5	8.5	76.5	47.5	42.5
Glutamine	Gln, Q	8	16.3	10.5	9.5	130.4	84.0	76.0
Glycine	Gly, G	1	11.7	14.5	11.0	11.7	14.5	11.0
Proline	Pro, P	3	20.3	14.5	14.5	60.9	43.5	43.5
Serine	Ser, S	6	11.7	14.5	11.0	70.2	87.0	66.0
Tyrosine	Tyr, Y	7	50.0	59.0	2.5	350.0	413.0	17.5
EAAs								
Histidine	His, H	14	38.3	29.0	-	536.2	406.0	-
Isoleucine	Ile, I	2	32.3	38.0	-	64.6	76.0	-
Leucine	Leu, L	2	27.3	37.0	-	54.6	74.0	-
Lysine	Lys, K	8	30.3	36.0	-	242.4	288.0	-
Methionine	Met, M	13	34.3	36.5	-	445.9	474.5	-
Phenylalanine	Phe, F	4	52.0	61.0	-	208.0	244.0	-
Threonine	Thr, T	6	18.7	21.5	-	112.2	129.0	-
Tryptophan	Trp, W	12	74.3	75.5	-	891.6	906.0	-
Valine	Val, V	2	23.3	29.0	-	46.6	58.0	-

*the AA decay rate is taken from Krick *et al.,* 2014.

The b20, y20, and h11 metric are the energy cost in the biosynthesis process, and the B20, Y20, and H11 metric are the biosynthetic cost normalized by AA decay rates.

Supplementary Table 2. Correlation between the biosynthetic cost and the experimentally quantified abundance of AAs that are hydrolyzed from proteins or free in animal tissues.

Source of AAs	Study	B20				Y20				H11			
		# of AAs	*r*	*P* value	*P′*	# of AAs	*r*	*P* value	*P′*	# of AAs	*r*	*P* value	*P′*
Hydrolyzed from proteins	Rat (Wu et al., 2013)	20	-0.91	3.46E-08	0	20	-0.92	7.92E-09	0	11	-0.63	0.037	0.0169
	Sheep (Wu et al., 2013)	20	-0.91	3.08E-08	0	20	-0.92	7.42E-09	0	11	-0.64	0.036	0.0169
	Pig (Wu et al., 2013)	20	-0.91	3.12E-08	0	20	-0.92	6.32E-09	0	11	-0.65	0.031	0.0145
	Chicken (Wu et al., 2013)	20	-0.91	3.62E-08	0	20	-0.92	9.23E-09	0	11	-0.63	0.038	0.0175
Free AAs in human tissues	Human liver, female (Blekhman et al., 2014)	20	-0.74	1.96E-04	0.0001	20	-0.73	2.33E-04	0	11	-0.78	0.005	0.0021
	Human liver, male (Blekhman et al., 2014)	20	-0.78	5.43E-05	0.0001	20	-0.79	4.07E-05	0	11	-0.80	0.003	0.0019
	Chimp liver, female (Blekhman et al., 2014)	20	-0.74	1.96E-04	0	20	-0.73	2.33E-04	0	11	-0.78	0.005	0.0021
	Chimp liver, male (Blekhman, et al., 2014)	20	-0.78	4.22E-05	0.0001	20	-0.78	4.18E-05	0	11	-0.72	0.012	0.0049
	Rhesus liver, female (Blekhman, et al., 2014)	20	-0.74	1.79E-04	0	20	-0.76	1.03E-04	0	11	-0.75	0.008	0.003

Rhesus liver, male (Blekhman, et al., 2014)	20	-0.76	9.98E-05	0	20	-0.76	9.35E-05	0	11	-0.73	0.010	0.0035
Human serum (Dereziński, et al., 2017)	20	-0.65	1.73E-03	0.0004	20	-0.69	7.18E-04	0.0003	11	-0.67	0.023	0.0105
Mouse liver (Takach, et al., 2014)	19	-0.75	2.09E-04	0	19	-0.76	1.74E-04	0.0001	10	-0.76	0.010	0.0051
Mouse kidney (Takach, et al., 2014)	20	-0.83	5.08E-06	0	20	-0.81	1.48E-05	0.0001	11	-0.71	0.013	0.0053

Pearson's *r* and *P* are obtained from the correlation tests between amino acid abundance (log_2 transformed) and biosynthetic cost.
P' is the fraction of simulations (10,000 replicates) that yield a smaller Pearson's *r* value compared to the observed *r*.

Supplementary Table 3. Pearson's *r* between the biosynthetic cost and the usage of AAs in human normal tissues (mRNA-Seq data for 27 tissues, and proteomic data for 30 tissues or cell types) after considering gene expression level.

Data type	Tissue/Cell	Number of genes expressed	Y20		B20		H11	
			r	*P* value	*r*	*P* value	*r*	*P* value
mRNA	Adipose tissue	15,893	-0.92	9.2E-09	-0.93	2.2E-09	-0.75	7.8E-03
mRNA	Adrenal gland	15,986	-0.92	8.4E-09	-0.93	2.3E-09	-0.76	6.9E-03
mRNA	Appendix	16,293	-0.92	1.4E-08	-0.93	4.2E-09	-0.73	1.0E-02
mRNA	Bone marrow	14,403	-0.91	2.1E-08	-0.92	9.5E-09	-0.72	1.2E-02
mRNA	Brain	16,325	-0.92	7.8E-09	-0.93	2.3E-09	-0.74	9.5E-03
mRNA	Colon	16,367	-0.92	1.3E-08	-0.93	2.9E-09	-0.74	9.1E-03
mRNA	Duodenum	16,108	-0.92	1.1E-08	-0.93	1.9E-09	-0.76	6.1E-03
mRNA	Endometrium	16,164	-0.92	1.3E-08	-0.93	5.0E-09	-0.73	1.1E-02
mRNA	Esophagus	16,075	-0.92	9.3E-09	-0.93	2.1E-09	-0.74	9.9E-03
mRNA	Gall bladder	16,291	-0.92	1.6E-08	-0.93	4.4E-09	-0.72	1.2E-02
mRNA	Heart	15,450	-0.93	4.5E-09	-0.94	1.1E-09	-0.77	5.4E-03
mRNA	Kidney	16,314	-0.92	1.1E-08	-0.93	2.2E-09	-0.76	6.5E-03
mRNA	Liver	15,219	-0.89	1.6E-07	-0.91	2.5E-08	-0.73	1.1E-02
mRNA	Lung	16,273	-0.91	2.2E-08	-0.92	7.1E-09	-0.72	1.2E-02
mRNA	Lymph node	15,767	-0.91	1.7E-08	-0.92	6.1E-09	-0.73	1.0E-02
mRNA	Ovary	15,377	-0.92	1.6E-08	-0.92	6.0E-09	-0.73	1.1E-02
mRNA	Pancreas	15,338	-0.80	2.2E-05	-0.84	3.6E-06	-0.59	5.7E-02
mRNA	Placenta	15,885	-0.91	2.0E-08	-0.93	5.4E-09	-0.72	1.2E-02
mRNA	Prostate	16,495	-0.92	1.3E-08	-0.93	3.9E-09	-0.74	8.9E-03
mRNA	Salivary gland	15,813	-0.94	1.3E-09	-0.94	6.9E-10	-0.78	4.3E-03
mRNA	Skin	15,870	-0.92	5.6E-09	-0.93	2.4E-09	-0.75	8.0E-03
mRNA	Small intestine	16,265	-0.92	1.1E-08	-0.93	2.2E-09	-0.76	6.9E-03
mRNA	Spleen	15,838	-0.91	2.5E-08	-0.92	1.2E-08	-0.72	1.2E-02
mRNA	Stomach	16,386	-0.92	1.4E-08	-0.94	1.5E-09	-0.76	6.9E-03
mRNA	Testis	18,140	-0.92	1.6E-08	-0.92	7.7E-09	-0.71	1.4E-02
mRNA	Thyroid gland	15,608	-0.91	2.9E-08	-0.92	8.9E-09	-0.71	1.5E-02
mRNA	Urinary bladder	16,020	-0.92	1.2E-08	-0.93	3.0E-09	-0.74	9.7E-03

protein	Adult adrenal	8,677	-0.91	2.0E-08	-0.92	5.8E-09	-0.72	1.2E-02
protein	Adult colon	8,096	-0.91	4.0E-08	-0.92	1.1E-08	-0.69	2.0E-02
protein	Adult esophagus	5,076	-0.89	1.8E-07	-0.91	2.7E-08	-0.75	7.8E-03
protein	Adult frontal cortex	9,252	-0.92	9.3E-09	-0.93	3.5E-09	-0.73	1.0E-02
protein	Adult gallbladder	7,811	-0.90	7.9E-08	-0.91	2.4E-08	-0.66	2.7E-02
protein	Adult heart	6,677	-0.84	3.3E-06	-0.87	7.2E-07	-0.74	9.3E-03
protein	Adult kidney	6,882	-0.91	3.6E-08	-0.92	8.4E-09	-0.70	1.7E-02
protein	Adult liver	9,344	-0.91	1.7E-08	-0.93	3.3E-09	-0.74	8.7E-03
protein	Adult lung	6,836	-0.90	4.5E-08	-0.91	1.6E-08	-0.68	2.2E-02
protein	Adult ovary	10,466	-0.91	2.8E-08	-0.92	7.2E-09	-0.70	1.6E-02
protein	Adult pancreas	9,380	-0.91	2.6E-08	-0.92	6.0E-09	-0.70	1.7E-02
protein	Adult prostate	9,174	-0.92	1.5E-08	-0.93	5.2E-09	-0.71	1.5E-02
protein	Adult rectum	8,216	-0.90	5.1E-08	-0.91	1.7E-08	-0.67	2.3E-02
protein	Adult retina	10,337	-0.92	1.0E-08	-0.93	2.5E-09	-0.74	9.2E-03
protein	Adult spinal cord	7,755	-0.92	1.2E-08	-0.93	4.4E-09	-0.73	1.0E-02
protein	Adult testis	11,131	-0.91	2.5E-08	-0.92	6.8E-09	-0.70	1.7E-02
protein	Adult urinary bladder	7,777	-0.92	1.4E-08	-0.93	4.6E-09	-0.72	1.3E-02
protein	B cells	9,312	-0.91	1.7E-08	-0.92	6.8E-09	-0.74	9.6E-03
protein	Cd4 cells	7970	-0.92	1.6E-08	-0.92	6.0E-09	-0.74	8.7E-03
protein	Cd8 cells	10,080	-0.92	1.3E-08	-0.93	4.8E-09	-0.75	8.1E-03
protein	Fetal brain	8,043	-0.92	7.7E-09	-0.94	1.5E-09	-0.75	8.5E-03
protein	Fetal gut	8,858	-0.91	4.1E-08	-0.92	8.6E-09	-0.71	1.5E-02
protein	Fetal heart	9,442	-0.85	1.7E-06	-0.88	3.3E-07	-0.74	8.7E-03
protein	Fetal liver	8,606	-0.91	1.7E-08	-0.93	3.1E-09	-0.75	7.6E-03
protein	Fetal ovary	9,327	-0.92	1.0E-08	-0.93	2.3E-09	-0.74	9.6E-03
protein	Fetal testis	8,130	-0.92	1.2E-08	-0.93	2.1E-09	-0.75	7.2E-03
protein	Monocytes	7,612	-0.92	1.6E-08	-0.93	3.2E-09	-0.74	9.6E-03
protein	Nk cells	8,673	-0.92	1.3E-08	-0.93	3.8E-09	-0.75	8.5E-03
protein	Placenta	6,240	-0.92	9.5E-09	-0.93	1.6E-09	-0.75	8.1E-03
protein	Platelets	6,269	-0.91	2.8E-08	-0.92	7.0E-09	-0.70	1.7E-02

The mRNA-Seq data were retrieved from (Fagerberg et al., 2014), and only genes with RPKM > 0.1 were used in the analysis. The proteomic data were taken from (Kim et al., 2014).

Supplementary Table 4 DAVID gene ontology analysis results of top 6,000 genes with highest $ECPA_{gene}$ and bottom 6,000 genes with lowest $ECPA_{gene}$.

Category	Term ID	Term description	Gene count	Adjusted *P* (Benjamini)
**Top 6,000 genes with highest $ECPA_{gene}$**				
GOTERM_BP	GO:0007186	G-protein coupled receptor signaling pathway	591	5.5E-111
GOTERM_BP	GO:0050911	detection of chemical stimulus involved in sensory perception of smell	337	1.9E-96
GOTERM_BP	GO:0007608	sensory perception of smell	114	1.4E-23
GOTERM_BP	GO:0007166	cell surface receptor signaling pathway	151	5.2E-15
GOTERM_BP	GO:0006355	regulation of transcription, DNA-templated	583	1.6E-11

GOTERM_BP	GO:0070098	chemokine-mediated signaling pathway	50	9.7E-09
GOTERM_BP	GO:0006486	protein glycosylation	64	5.1E-06
GOTERM_BP	GO:0006508	proteolysis	204	7.1E-05
GOTERM_BP	GO:0006955	immune response	176	7.5E-05
GOTERM_BP	GO:0007204	positive regulation of cytosolic calcium ion concentration	66	1.3E-03
GOTERM_BP	GO:0016579	protein deubiquitination	52	1.5E-03
GOTERM_BP	GO:0007267	cell-cell signaling	108	4.9E-03
GOTERM_MF	GO:0046872	metal ion binding	901	1.1E-43
GOTERM_MF	GO:0003676	nucleic acid binding	465	4.0E-30
GOTERM_MF	GO:0004888	transmembrane signaling receptor activity	130	2.4E-18
GOTERM_MF	GO:0004842	ubiquitin-protein transferase activity	149	5.8E-07
GOTERM_MF	GO:0004222	metalloendopeptidase activity	64	1.4E-06
GOTERM_MF	GO:0004252	serine-type endopeptidase activity	118	4.9E-06
GOTERM_MF	GO:0008201	heparin binding	81	7.0E-06
GOTERM_MF	GO:0030246	carbohydrate binding	95	7.2E-06
KEGG_PATHWAY	hsa04740	olfactory transduction	340	1.1E-112
KEGG_PATHWAY	hsa04080	neuroactive ligand-receptor interaction	174	5.0E-25
KEGG_PATHWAY	hsa04060	cytokine-cytokine receptor interaction	136	4.7E-16
KEGG_PATHWAY	hsa04742	taste transduction	34	7.3E-07
KEGG_PATHWAY	hsa04610	complement and coagulation cascades	43	2.8E-05
KEGG_PATHWAY	hsa04550	signaling pathways regulating pluripotency of stem cells	70	3.0E-04
KEGG_PATHWAY	hsa04310	Wnt signaling pathway	68	6.8E-04
**Bottom 6,000 genes with lowest $ECPA_{gene}$**				
GOTERM_BP	GO:0007156	homophilic cell adhesion via plasma membrane adhesion molecules	122	2.9E-32
GOTERM_BP	GO:0006366	transcription from RNA polymerase II promoter	278	3.1E-31
GOTERM_BP	GO:0006334	nucleosome assembly	73	3.1E-10
GOTERM_BP	GO:0008380	RNA splicing	79	1.1E-04
GOTERM_BP	GO:0031047	gene silencing by RNA	57	2.8E-04
GOTERM_BP	GO:0043488	regulation of mRNA stability	53	5.8E-04
GOTERM_BP	GO:0044267	cellular protein metabolic process	56	4.7E-03
GOTERM_CC	GO:0000790	nuclear chromatin	112	1.1E-14
GOTERM_CC	GO:0005581	collagen trimer	64	6.0E-13
GOTERM_CC	GO:0000786	nucleosome	65	6.5E-13
GOTERM_CC	GO:0005667	transcription factor complex	103	2.6E-10
GOTERM_CC	GO:0015629	actin cytoskeleton	103	1.6E-06
GOTERM_CC	GO:0005856	cytoskeleton	152	4.2E-05
GOTERM_CC	GO:0005815	microtubule organizing center	74	4.3E-05
GOTERM_CC	GO:0016607	nuclear speck	92	4.3E-05
GOTERM_CC	GO:0005874	microtubule	129	1.2E-04
GOTERM_CC	GO:0005813	centrosome	168	1.2E-04
GOTERM_CC	GO:0030529	intracellular ribonucleoprotein complex	66	1.3E-04
GOTERM_CC	GO:0000784	nuclear chromosome, telomeric region	61	1.1E-03

GO category “BP”, “CC” and “MF” stand for biological process, cellular component and molecular function, respectively.

Supplementary Table 5. Spearman's *rho* between $ECPA_{gene}$ and gene expression level in human normal tissues (mRNA-Seq data for 27 tissues, and proteomic data for 30 tissues or cell types).

Data type	Tissue/Cell	Number of genes expressed	Y20		B20		H11	
			r	*P* value	*r*	*P* value	*r*	*P* value
mRNA	Adipose tissue	15,893	-0.70	0.0E+00	-0.78	0.0E+00	-0.71	1.1E-16
mRNA	Adrenal gland	15,986	-0.61	0.0E+00	-0.66	0.0E+00	-0.65	0.0E+00
mRNA	Appendix	16,293	-0.55	5.6E-09	-0.65	0.0E+00	-0.45	3.2E-06
mRNA	Bone marrow	14,403	-0.61	0.0E+00	-0.65	0.0E+00	-0.44	4.8E-06
mRNA	Brain	16,325	-0.77	0.0E+00	-0.81	0.0E+00	-0.69	0.0E+00
mRNA	Colon	16,367	-0.61	0.0E+00	-0.77	0.0E+00	-0.64	0.0E+00
mRNA	Duodenum	16,108	-0.63	0.0E+00	-0.76	0.0E+00	-0.70	0.0E+00
mRNA	Endometrium	16,164	-0.70	0.0E+00	-0.77	0.0E+00	-0.55	6.7E-09
mRNA	Esophagus	16,075	-0.61	0.0E+00	-0.70	0.0E+00	-0.56	2.4E-09
mRNA	Gall bladder	16,291	-0.43	8.1E-06	-0.58	0.0E+00	-0.49	4.1E-07
mRNA	Heart	15,450	-0.66	0.0E+00	-0.73	0.0E+00	-0.71	0.0E+00
mRNA	Kidney	16,314	-0.60	0.0E+00	-0.71	0.0E+00	-0.76	0.0E+00
mRNA	Liver	15,219	-0.35	4.7E-04	-0.46	1.7E-06	-0.63	0.0E+00
mRNA	Lung	16,273	-0.61	0.0E+00	-0.69	0.0E+00	-0.66	0.0E+00
mRNA	Lymph node	15,767	-0.50	1.3E-07	-0.64	0.0E+00	-0.27	7.1E-03
mRNA	Ovary	15,377	-0.70	0.0E+00	-0.74	0.0E+00	-0.58	0.0E+00
mRNA	Pancreas	15,338	-0.73	0.0E+00	-0.76	0.0E+00	-0.76	0.0E+00
mRNA	Placenta	15,885	-0.54	1.0E-08	-0.66	0.0E+00	-0.56	2.5E-09
mRNA	Prostate	16,495	-0.71	0.0E+00	-0.77	0.0E+00	-0.74	0.0E+00
mRNA	Salivary gland	15,813	-0.75	0.0E+00	-0.79	0.0E+00	-0.73	0.0E+00
mRNA	Skin	15,870	-0.73	0.0E+00	-0.79	0.0E+00	-0.71	0.0E+00
mRNA	Small intestine	16,265	-0.54	1.2E-08	-0.74	0.0E+00	-0.58	8.7E-11
mRNA	Spleen	15,838	-0.62	0.0E+00	-0.64	0.0E+00	-0.59	0.0E+00
mRNA	Stomach	16,386	-0.71	0.0E+00	-0.79	0.0E+00	-0.70	0.0E+00
mRNA	Testis	18,140	-0.75	0.0E+00	-0.80	0.0E+00	-0.52	5.3E-08
mRNA	Thyroid gland	15,608	-0.49	4.3E-07	-0.62	0.0E+00	-0.55	6.7E-09
mRNA	Urinary bladder	16,020	-0.39	7.6E-05	-0.62	0.0E+00	-0.38	9.9E-05
protein	Adult adrenal	8,677	-0.20	4.4E-02	-0.40	4.1E-05	-0.58	2.2E-10
protein	Adult colon	8,096	-0.33	8.9E-04	-0.49	2.5E-07	-0.56	1.4E-09
protein	Adult esophagus	5,076	-0.21	3.6E-02	-0.37	1.3E-04	-0.31	1.7E-03
protein	Adult frontal cortex	9,252	-0.41	2.8E-05	-0.57	7.1E-10	-0.59	1.3E-10
protein	Adult gallbladder	7,811	-0.35	3.6E-04	-0.48	4.7E-07	-0.51	4.4E-08
protein	Adult heart	6,677	-0.37	1.6E-04	-0.50	8.7E-08	-0.56	1.2E-09
protein	Adult kidney	6,882	-0.33	9.5E-04	-0.49	2.3E-07	-0.74	1.1E-18
protein	Adult liver	9,344	-0.21	3.2E-02	-0.47	9.6E-07	-0.54	5.4E-09
protein	Adult lung	6,836	-0.34	4.5E-04	-0.49	2.1E-07	-0.59	7.1E-11
protein	Adult ovary	10,466	-0.26	8.1E-03	-0.42	1.4E-05	-0.46	1.8E-06
protein	Adult pancreas	9,380	-0.53	1.4E-08	-0.68	5.5E-15	-0.64	7.9E-13
protein	Adult prostate	9,174	-0.46	1.2E-06	-0.52	2.9E-08	-0.51	6.4E-08

protein	Adult rectum	8,216	-0.25	1.3E-02	-0.37	1.8E-04	-0.55	4.0E-09
protein	Adult retina	10,337	-0.44	6.0E-06	-0.58	3.4E-10	-0.56	1.4E-09
protein	Adult spinal cord	7,755	-0.38	1.1E-04	-0.53	1.2E-08	-0.54	5.7E-09
protein	Adult testis	11,131	-0.51	4.6E-08	-0.65	3.6E-13	-0.53	2.0E-08
protein	Adult urinary bladder	7,777	-0.37	1.2E-04	-0.50	1.0E-07	-0.45	2.2E-06
protein	B cells	9,312	-0.48	4.6E-07	-0.55	3.6E-09	-0.48	4.6E-07
protein	Cd4 cells	7,970	-0.36	2.0E-04	-0.54	5.9E-09	-0.51	6.3E-08
protein	Cd8 cells	10,080	-0.60	5.8E-11	-0.68	3.9E-15	-0.62	5.8E-12
protein	Fetal brain	8,043	-0.35	4.2E-04	-0.52	2.9E-08	-0.48	3.7E-07
protein	Fetal gut	8,858	-0.18	7.5E-02	-0.35	4.3E-04	-0.51	6.9E-08
protein	Fetal heart	9,442	-0.62	4.8E-12	-0.72	2.8E-17	-0.80	4.2E-23
protein	Fetal liver	8,606	-0.35	3.4E-04	-0.55	3.8E-09	-0.55	2.5E-09
protein	Fetal ovary	9,327	-0.40	4.6E-05	-0.57	4.2E-10	-0.58	3.6E-10
protein	Fetal testis	8,130	-0.36	1.9E-04	-0.55	2.5E-09	-0.62	4.4E-12
protein	Monocytes	7,612	-0.20	4.8E-02	-0.36	2.0E-04	-0.41	2.7E-05
protein	Nk cells	8,673	-0.43	7.1E-06	-0.60	4.8E-11	-0.59	1.5E-10
protein	Placenta	6,240	-0.23	2.0E-02	-0.36	2.1E-04	-0.54	7.6E-09
protein	Platelets	6,269	-0.05	6.2E-01	-0.23	2.0E-02	-0.26	9.6E-03

The mRNA-Seq data were retrieved from (Fagerberg et al., 2014), and only genes with RPKM > 0.1 were used in the analysis. The proteomic data were taken from (Kim et al., 2014). For each tissue, genes were divided into 100 groups with increasing expression levels and the median expression level and median $ECPA_{gene}$ in each group were used in the correlation analysis.

Supplementary Table 6. Spearman's rank *rho* between $ECPA_{gene}$ and gene expression level in different matched cancer and normal tissues using TCGA mRNA-Seq data.

Acronym	Genes expressed	Sample type	Number of samples	*rho* (Y20)			*rho* (B20)			*rho* (H11)		
				Mean	Lower	Upper	Mean	Lower	Upper	Mean	Lower	Upper
BLCA	14,825	Tumor	408	-0.77	-0.77	-0.76	-0.81	-0.81	-0.80	-0.81	-0.81	-0.80
BLCA	14,825	Normal	19	-0.75	-0.77	-0.73	-0.79	-0.81	-0.78	-0.79	-0.82	-0.77
BRCA	15,198	Normal	114	-0.61	-0.63	-0.58	-0.70	-0.72	-0.69	-0.69	-0.72	-0.67
BRCA	15,198	Tumor	1097	-0.70	-0.71	-0.70	-0.77	-0.77	-0.76	-0.74	-0.75	-0.74
COAD	14,942	Normal	41	-0.70	-0.72	-0.68	-0.76	-0.78	-0.75	-0.79	-0.80	-0.78
COAD	14,942	Tumor	286	-0.74	-0.75	-0.74	-0.80	-0.80	-0.79	-0.80	-0.81	-0.80
ESCA	15,317	Tumor	184	-0.76	-0.77	-0.75	-0.81	-0.82	-0.81	-0.78	-0.79	-0.77
ESCA	15,317	Normal	11	-0.75	-0.76	-0.73	-0.80	-0.82	-0.78	-0.78	-0.82	-0.74
HNSC	15,032	Tumor	520	-0.75	-0.75	-0.74	-0.80	-0.81	-0.80	-0.80	-0.81	-0.80
HNSC	15,032	Normal	44	-0.76	-0.77	-0.74	-0.81	-0.82	-0.80	-0.81	-0.82	-0.80
KICH	14,562	Tumor	66	-0.71	-0.73	-0.69	-0.77	-0.78	-0.76	-0.81	-0.82	-0.80
KICH	14,562	Normal	25	-0.72	-0.75	-0.69	-0.77	-0.80	-0.75	-0.83	-0.85	-0.82
KIRC	15,137	Tumor	533	-0.64	-0.65	-0.63	-0.72	-0.73	-0.71	-0.78	-0.79	-0.77
KIRC	15,137	Normal	72	-0.63	-0.65	-0.60	-0.72	-0.73	-0.71	-0.78	-0.79	-0.76
KIRP	14,847	Tumor	290	-0.76	-0.77	-0.75	-0.80	-0.81	-0.80	-0.85	-0.85	-0.84
KIRP	14,847	Normal	32	-0.74	-0.75	-0.72	-0.78	-0.80	-0.77	-0.85	-0.85	-0.84

LIHC	14,056	Tumor	371	-0.64	-0.65	-0.63	-0.72	-0.72	-0.71	-0.84	-0.85	-0.84
LIHC	14,056	Normal	50	-0.51	-0.52	-0.49	-0.62	-0.63	-0.61	-0.84	-0.85	-0.84
LUAD	15,360	Tumor	515	-0.69	-0.70	-0.68	-0.76	-0.76	-0.75	-0.79	-0.80	-0.79
LUAD	15,360	Normal	59	-0.64	-0.66	-0.62	-0.72	-0.73	-0.71	-0.79	-0.81	-0.78
LUSC	15,428	Tumor	503	-0.70	-0.71	-0.70	-0.78	-0.78	-0.77	-0.78	-0.79	-0.78
LUSC	15,428	Normal	51	-0.63	-0.65	-0.60	-0.71	-0.73	-0.69	-0.78	-0.80	-0.76
PRAD	15,258	Tumor	497	-0.75	-0.75	-0.75	-0.80	-0.80	-0.80	-0.82	-0.83	-0.82
PRAD	15,258	Normal	52	-0.73	-0.75	-0.71	-0.79	-0.80	-0.78	-0.82	-0.83	-0.81
STAD	14,984	Tumor	380	-0.76	-0.77	-0.75	-0.82	-0.82	-0.81	-0.81	-0.82	-0.81
STAD	14,984	Normal	37	-0.73	-0.76	-0.70	-0.79	-0.81	-0.77	-0.82	-0.84	-0.80
THCA	14,964	Tumor	505	-0.72	-0.73	-0.72	-0.77	-0.77	-0.76	-0.80	-0.81	-0.80
THCA	14,964	Normal	59	-0.70	-0.71	-0.68	-0.75	-0.76	-0.73	-0.75	-0.76	-0.73
UCEC	15,065	Tumor	176	-0.79	-0.79	-0.78	-0.82	-0.83	-0.82	-0.83	-0.84	-0.82
UCEC	15,065	Normal	24	-0.78	-0.80	-0.75	-0.81	-0.83	-0.79	-0.81	-0.82	-0.79

In each cancer type, the genes with a median expression level RPKM > 0.1 across all the samples were analyzed. For each sample of each cancer type, genes were divided into 100 groups with increasing expression level, and the median expression level and median ECPA in each group were used in the correlation analysis. "Lower" and "Upper" denote lower and upper bound of 95% confidence interval for the mean value of Spearman's *rho*.

Supplementary Table 7. Correlation between cost of amino acids and experimentally measured amino acid abundance in tumor and normal tissue samples compared to that of randomly shuffled cost of AAs for 10,000 times.

Study	Reference	# of AAs	Tumor			Normal		
			r	*P* value	*P′*	*r*	*P* value	*P′*
**Y20**								
Breaster cancer, plasma	Miyagi et al., 2011	17	-0.68	2.9E-03	0.0009	-0.64	5.3E-03	0.0025
Cervical cancer, plasma	Hasim et al., 2013	18	-0.56	1.6E-02	0.0065	-0.70	1.4E-03	0.0008
Colorectal carcinoma, plasma	Bener et al., 2006	18	-0.77	2.1E-04	0	-0.61	6.9E-03	0.0028
Colorectal carcinoma, plasma	Miyagi et al., 2011	17	-0.68	2.8E-03	0.001	-0.65	4.8E-03	0.0022
Gastric cancer, plasma	Miyagi et al., 2011	17	-0.67	3.0E-03	0.0012	-0.65	4.7E-03	0.0021
Lung cancer, plasma	Miyagi et al., 2011	17	-0.68	2.6E-03	0.0008	-0.65	4.7E-03	0.0021
Prostate cancer, plasma	Miyagi et al., 2011	17	-0.66	3.8E-03	0.0018	-0.65	4.8E-03	0.0021
Prostate cancer, serum	Dereziński et al., 2017	20	-0.71	5.1E-04	0.0003	-0.69	7.2E-04	0.0003
Hepatocellular carcinoma	Takashi et al., 1995	20	-0.75	1.6E-04	0	-0.62	3.8E-03	0.002
Lung cancer	Kami et al., 2013	19	-0.79	6.2E-05	0	-0.75	2.5E-04	0
Prostate cancer	Kami et al., 2013	19	-0.76	1.4E-04	0	-0.77	1.1E-04	0
Squamous cell carcinoma	Leme et al., 2012	13	-0.78	1.9E-03	0.0016	-0.84	3.0E-04	0.0003
**B20**								
Breaster cancer, plasma	Miyagi et al., 2011	17	-0.62	8.5E-03	0.0042	-0.58	1.4E-02	0.0072
Cervical cancer, plasma	Hasim et al., 2013	18	-0.58	1.2E-02	0.0047	-0.70	1.3E-03	0.0008
Colorectal carcinoma, plasma	Bener et al., 2006	18	-0.73	6.5E-04	0.0003	-0.61	7.0E-03	0.0034
Colorectal carcinoma, plasma	Miyagi et al., 2011	17	-0.62	8.4E-03	0.0047	-0.59	1.3E-02	0.0063

Gastric cancer, plasma	Miyagi et al., 2011	17	-0.61	9.1E-03	0.0047	-0.59	1.3E-02	0.0062
Lung cancer, plasma	Miyagi et al., 2011	17	-0.62	7.7E-03	0.0038	-0.59	1.3E-02	0.0063
Prostate cancer, plasma	Miyagi et al., 2011	17	-0.60	1.1E-02	0.0051	-0.59	1.3E-02	0.0063
Prostate cancer, serum	Dereziński et al., 2017	20	-0.67	1.2E-03	0.0003	-0.65	1.7E-03	0.0004
Hepatocellular carcinoma	Takashi et al., 1995	20	-0.72	3.9E-04	0.0002	-0.60	5.3E-03	0.0033
Lung cancer	Kami et al., 2013	19	-0.72	5.1E-04	0	-0.69	1.1E-03	0.0001
Prostate cancer	Kami et al., 2013	19	-0.69	9.8E-04	0.0002	-0.70	7.9E-04	0.0004
Squamous cell carcinoma	Leme et al., 2012	13	-0.71	6.2E-03	0.0036	-0.80	9.3E-04	0.0005
**H11**								
Breaster cancer, plasma	Miyagi et al., 2011	8	-0.35	4.0E-01	0.192	-0.32	4.4E-01	0.2143
Cervical cancer, plasma	Hasim et al., 2013	10	-0.76	1.0E-02	0.0055	-0.75	1.2E-02	0.0054
Colorectal carcinoma, plasma	Bener et al., 2006	9	-0.51	1.6E-01	0.0826	-0.50	1.7E-01	0.0803
Colorectal carcinoma, plasma	Miyagi et al., 2011	8	-0.32	4.4E-01	0.2123	-0.32	4.3E-01	0.2117
Gastric cancer, plasma	Miyagi et al., 2011	8	-0.31	4.5E-01	0.2211	-0.32	4.3E-01	0.2127
Lung cancer, plasma	Miyagi et al., 2011	8	-0.36	3.9E-01	0.1878	-0.33	4.3E-01	0.21
Prostate cancer, plasma	Miyagi et al., 2011	8	-0.33	4.3E-01	0.2068	-0.32	4.4E-01	0.2133
Prostate cancer, serum	Dereziński et al., 2017	11	-0.63	3.7E-02	0.0174	-0.67	2.3E-02	0.0105
Hepatocellular carcinoma	Takashi et al., 1995	11	-0.46	1.5E-01	0.0784	-0.33	3.2E-01	0.1629
Lung cancer	Kami et al., 2013	10	-0.28	4.3E-01	0.2102	-0.28	4.3E-01	0.2119
Prostate cancer	Kami et al., 2013	10	-0.43	2.2E-01	0.1009	-0.38	2.8E-01	0.139
Squamous cell carcinoma	Leme et al., 2012	7	-0.10	8.4E-01	0.4166	-0.37	4.2E-01	0.2062

Pearson's *r* and *P* are obtained from the correlation tests between amino acid abundance ($\log_2$ transformed) and biosynthetic cost.
P' is the fraction of simulations (10,000 replicates) that yield a smaller Pearson's *r* value compared to the observed *r*.

Supplementary Table 8. Number of genes that have expression level significantly correlated with the ECPA$_{cell}$ (Y20) among samples in each of the 31 TCGA cancer types with at least 50 samples.

Cancer type	Number of genes	Positively correlated genes (FDR adjusted $P < 0.05$)	Negatively correlated genes (FDR adjusted $P < 0.05$)
ACC	14,076	379	273
BLCA	14,558	2,118	2,743
BRCA	14,607	4,848	5,665
CESC	14,421	2,963	2,545
COAD	13,991	3,315	4,769
ESCA	14,796	4,294	4,346
GBM	14,457	4,595	4,759
HNSC	14,424	5,651	3,733
KICH	14,156	2,814	2,569
KIRC	14,478	4,760	5,814
KIRP	14,382	5,091	4,155
LAML	13,829	3,970	3,824
LGG	14,257	5,398	4,345
LIHC	14,089	3,629	5,281
LUAD	14,815	4,763	4,609

LUSC	14,880	4,980	4,492
MESO	14,332	1,692	2,474
OV	14,675	3,320	4,408
PAAD	14,850	3,370	2,657
PCPG	14,178	3,337	2,827
PRAD	14,187	3,595	4,909
READ	14,090	787	1,729
SARC	14,782	2,766	3,028
SKCM	14,278	5,676	4,910
STAD	15,872	4,805	3,870
TGCT	15,173	5,047	5,382
THCA	13,962	5,161	5,142
THYM	14,338	5,109	3,645
UCEC	14,822	1,900	1,956
UCS	14,977	103	276
UVM	13,099	5,442	3,528

FDR: false discover rate was used for multiple testing correction.

Supplementary Table 9. Number of genes that had expression levels correlated with $ECPA_{cell}$ (Y20) in the same direction across different numbers of the 20 cancer types that have significantly lower $ECPA_{cell}$ in tumors or have $ECPA_{cell}$ associated with pathological stage of tumors or patient survival time.

Number of cancer types	Positively correlated genes (FDR adjusted $P < 0.05$)	Negatively correlated genes (FDR adjusted $P < 0.05$)
1	2,628	2,565
2	1,865	1,929
3	1,603	1,582
4	1,366	1,384
5	1,195	1,190
6	1,104	1,005
7	979	861
8	752	810
9	659	626
10	472	547
11	383	465
12	328	423
13	213	313
14	160	221
15	105	150
16	60	90
17	29	32
18	9	10
19	5	2

Supplementary Table 10. DAVID gene ontology analysis of genes that had expression levels correlated with $ECPA_{cell}$ in the same direction across samples in at least 10 of the 20 cancer types that have significantly lower $ECPA_{cell}$ in tumors or had $ECPA_{cell}$ associated with pathological stage of tumors or patient survival time for cost metric B20 (at least 11 of the 20 cancer types for cost metric H11).

Category	Term ID	Term Description	Count	Adjusted *P* (Benjamini)	$ECPA_{gene}$ (mean ± SE)
**Positively correlated genes (based on cost metric B20)**					
GOTERM_CC	GO:0005777	peroxisome	41	1.1E-10	137.7±2.1
KEGG_PATHWAY	hsa01212	Fatty acid metabolism	22	7.3E-06	134.1±2.8
GOTERM_BP	GO:0006888	ER to Golgi vesicle-mediated transport	41	1.8E-03	141.9±2.9
KEGG_PATHWAY	hsa00020	Citrate cycle (TCA cycle)	13	5.1E-03	137.2±4.8
GOTERM_MF	GO:0005525	GTP binding	69	5.1E-02	137.6±1.3
**Negatively correlated genes (based on cost metric B20)**					
GOTERM_CC	GO:0005913	cell-cell adherens junction	94	2.0E-11	126.0±1.2
GOTERM_BP	GO:0030574	collagen catabolic process	33	4.9E-09	118.6±5.2
KEGG_PATHWAY	hsa04512	ECM-receptor interaction	34	5.1E-07	126.1±4.7
GOTERM_BP	GO:0007062	sister chromatid cohesion	36	6.1E-05	133.3±2.4
KEGG_PATHWAY	hsa04151	PI3K-Akt signaling pathway	74	5.9E-04	132.9±2.6
GOTERM_MF	GO:0004672	protein kinase activity	83	6.4E-04	135.8±1.3
KEGG_PATHWAY	hsa03040	Spliceosome	36	1.1E-03	127.1±1.9
KEGG_PATHWAY	hsa04330	Notch signaling pathway	17	5.4E-03	146.7±6.8
GOTERM_CC	GO:0005871	kinesin complex	18	9.2E-03	125.5±2.8
GOTERM_CC	GO:0035097	histone methyltransferase complex	11	9.6E-03	127.6±5.3
KEGG_PATHWAY	hsa04974	Protein digestion and absorption	24	1.1E-02	97.0±2.8
**Positively correlated genes (based on cost metric H11)**					
GOTERM_CC	GO:0005777	peroxisome	36	2.6E-05	65.1±1.1
GOTERM_BP	GO:0006468	protein phosphorylation	113	3.0E-05	68.6±0.5
KEGG_PATHWAY	hsa04662	B cell receptor signaling pathway	25	4.5E-04	69.2±1.1
KEGG_PATHWAY	hsa00562	Inositol phosphate metabolism	24	1.4E-03	69.9±1.0
GOTERM_MF	GO:0004843	thiol-dependent ubiquitin-specific protease activity	27	6.2E-03	72.4±1.4
KEGG_PATHWAY	hsa00071	Fatty acid degradation	16	7.4E-03	63.6±1.3
KEGG_PATHWAY	hsa04620	Toll-like receptor signaling pathway	27	1.7E-02	72.9±1.5
KEGG_PATHWAY	hsa04370	VEGF signaling pathway	18	2.1E-02	70.7±1.0
GOTERM_CC	GO:0005913	cell-cell adherens junction	66	3.8E-02	65.9±0.6
**Negatively correlated genes (based on cost metric H11)**					
GOTERM_BP	GO:0006364	rRNA processing	137	3.2E-58	61.8±0.7
GOTERM_BP	GO:0006412	translation	139	6.2E-48	61.0±0.7
GOTERM_BP	GO:0000398	mRNA splicing, via spliceosome	88	2.4E-17	63.9±1.1
GOTERM_BP	GO:0043488	regulation of mRNA stability	45	9.2E-10	62.3±1.1
GOTERM_CC	GO:0000502	proteasome complex	28	3.2E-07	61.1±1.2
GOTERM_CC	GO:0005913	cell-cell adherens junction	79	2.5E-05	60.9±0.8
KEGG_PATHWAY	hsa00190	Oxidative phosphorylation	41	2.5E-04	58.8±1.4
GOTERM_CC	GO:0005655	nucleolar ribonuclease P complex	7	3.2E-03	70.6±2.8
GOTERM_BP	GO:0050852	T cell receptor signaling pathway	39	1.4E-02	63.9±1.2
GOTERM_BP	GO:0030574	collagen catabolic process	21	2.8E-02	54.1±2.5

The genomic background $ECPA_{gene}$ is 141.5±0.15 for cost metric B20 and 67.3±0.08 for H11.

Supplementary Table 11. Summary of the pan-cancer analysis with different cost metrics (B20, Y20, or H11) after excluding different categories of genes. The number and acronyms of the cancer types that showed significant results in each analysis are presented.

Gene category excluded	Cost metric	Number of cancer types with significantly lower $ECPA_{cell}$ in tumor compared to normal samples ($P < 0.05$)	Number of cancer types with significant negative correlation between $ECPA_{cell}$ and pathologic stage ($P < 0.05$)	Number of cancer types with significant negative association between $ECPA_{cell}$ and patient survival in Log-rank test of Cox model ($P < 0.05$)
**Enriched pathways in positively correlated genes**	Y20	**12** (LIHC, THCA, KIRC, KICH, KIRP, LUAD, COAD, LUSC, BRCA, STAD, ESCA, HNSC)	**9** (CHOL, ACC, CRC, KICH, LIHC, BLCA, LUAD, KIRC, THCA, BRCA)	**11** (MESO, SKCM, LIHC, KIRC, ACC, BLCA, LUAD, SARC, COAD, LAML, OV)
	B20	**11** (LIHC, THCA, KIRC, KICH, KIRP, LUAD, COAD, LUSC, BRCA, STAD, HNSC)	**9** (ACC, LIHC, CRC, KIRP, KIRC, LUAD, BLCA, THCA, BRCA)	**11** (MESO, SKCM, LIHC, KIRC, ACC, BLCA, LUAD, SARC, LAML, OV)
	H11	**13** (LIHC, THCA, KIRC, KICH, KIRP, LUAD, ESCA, COAD, LUSC, BRCA, STAD, ESCA, HNSC)	**6** (KIRC, LIHC, KIRP, LUAD, CRC, THCA)	**7** (MESO, SKCM, LIHC, KIRC, ACC, LUAD, LAML)
**Enriched pathways in negatively correlated genes**	Y20	**10** (LIHC, THCA, KIRC, LUAD, KICH, KIRP, BRCA, LUSC, UCEC, COAD)	**4** (LIHC, LUAD, CRC, BRCA)	**7** (MESO, SKCM, LIHC, KIRC, ACC, LUAD, LAML)
	B20	**11** (LIHC, THCA, KIRC, PRAD, LUAD, KICH, KIRP, BRCA, UCEC, LUSC, COAD)	**3** (LIHC, LUAD, BRCA)	**6** (MESO, SKCM, LIHC, KIRC, ACC, LUAD)
	H11	**14** (LIHC, THCA, KIRC, LUAD, STAD, ESCA, BRCA, KICH, LUSC, KIRP, PRAD, COAD, UCEC, BLCA)	**4** (LIHC, KIRC, THCA, LUAD)	**5** (SKCM, LIHC, KIRC, LUAD, LAML)
**Tumor suppressors**	Y20	**12** (LIHC, THCA, KIRC, KICH, KIRP, LUAD, COAD, LUSC, BRCA, STAD, ESCA, HNSC)	**8** (CHOL, ACC, CRC, LIHC, LUAD, BLCA, THCA, BRCA)	**10** (MESO, SKCM, LIHC, KIRC, ACC, BLCA, LUAD, SARC, LAML, OV)
	B20	**11** (LIHC, THCA, KIRC, KIRP, KICH, LUAD, LUSC, COAD, BRCA, STAD, HNSC)	**11** (CHOL, ACC, KICH, LIHC, LUAD, CRC, BLCA, KIRP, THCA, KIRC, BRCA)	**11** (MESO, SKCM, LIHC, KIRC, ACC, BLCA, LUAD, SARC, COAD, LAML, OV)
	H11	**13** (LIHC, THCA, KIRC, LUAD, KIRP, KICH, ESCA, STAD, LUSC, PRAD, BRCA, COAD, BLCA)	**7** (KICH, KIRC, LUAD, LIHC, KIRP, CRC, THCA)	**8** (MESO, SKCM, LIHC, KIRC, ACC, LUAD, SARC, LAML)
**Cancer driver genes**	Y20	**12** (LIHC, THCA, KIRC, KICH, KIRP, LUAD, COAD, LUSC, BRCA, STAD, ESCA, HNSC)	**8** (CHOL, ACC, CRC, LIHC, LUAD, BLCA, THCA, BRCA)	**12** (MESO, SKCM, LIHC, KIRC, ACC, BLCA, LUAD, SARC, COAD, LAML, OV, STAD)
	B20	**12** (LIHC, THCA, KIRC, PRAD, KICH, KIRP, LUAD, LUSC, COAD, BRCA, STAD, HMSC)	**10** (KICH, ACC, CRC, LIHC, LUAD, KIRP, BLCA, THCA, KIRC, BRCA)	**12** (MESO, SKCM, LIHC, KIRC, ACC, BLCA, LUAD, SARC, COAD, LAML, OV, STAD)

	H11	**13** (LIHC, THCA, KIRC, LUAD, KIRP, KICH, ESCA, STAD, PRAD, LUSC, BRCA, COAD, BLCA)	**7** (KIRC, LIHC, LUAD, KIRP, CRC, THCA, BRCA)	**8** (MESO, SKCM, LIHC, KIRC, ACC, LUAD, SARC, LAML)
**AA biosynthesis**	Y20	**11** (LIHC, THCA, KIRC, KICH, KIRP, LUAD, COAD, LUSC, BRCA, STAD, HNSC)	**9** (CHOL, KICH, ACC, CRC, LIHC, LUAD, BLCA, THCA, BRCA)	**11** (MESO, SKCM, LIHC, KIRC, ACC, BLCA, LUAD, SARC, COAD, LAML, OV)
	B20	**12** (LIHC, THCA, KIRC, PRAD, KICH, KIRP, LUAD, LUSC, COAD, BRCA, STAD, HNSC)	**10** (KICH, ACC, CRC, LIHC, LUAD, KIRP, BLCA, THCA, KIRC, BRCA)	**12** (MESO, SKCM, LIHC, KIRC, ACC, BLCA, LUAD, SARC, COAD, LAML, OV, STAD)
	H11	**13** (LIHC, THCA, KIRC, LUAD, KIRP, KICH, ESCA, STAD, PRAD, LUSC, BRCA, COAD, BLCA)	**6** (KIRC, LIHC, LUAD, KIRP, CRC, THCA)	**8** (MESO, SKCM, LIHC, KIRC, ACC, LUAD, SARC, LAML)
**AA transport**	Y20	**11** (LIHC, THCA, KIRC, KICH, KIRP, LUAD, COAD, LUSC, BRCA, STAD, HNSC)	**9** (CHOL, KICH, ACC, CRC, LIHC, LUAD, BLCA, THCA, BRCA)	**11** (MESO, SKCM, LIHC, KIRC, ACC, BLCA, LUAD, SARC, COAD, LAML, OV)
	B20	**12** (LIHC, THCA, KIRC, PRAD, KICH, KIRP, LUAD, LUSC, COAD, BRCA, STAD, HNSC)	**10** (KICH, ACC, LIHC, CRC, LUAD, KIRP, BLCA, THCA, KIRC, BRCA)	**11** (MESO, SKCM, LIHC, KIRC, ACC, BLCA, LUAD, SARC, COAD, LAML, OV)
	H11	**13** (LIHC, THCA, KIRC, LUAD, KIRP, KICH, ESCA, STAD, PRAD, LUSC, BRCA, COAD, BLCA)	**6** (KIRC, LIHC, LUAD, KIRP, THCA, CRC)	**8** (MESO, SKCM, LIHC, KIRC, ACC, LUAD, SARC, LAML)

Supplementary Table 12. Summary of the source of experimentally measured abundance of AAs.

Study	Reference	Sample	Units
The amino acid composition of mammalian and bacterial cells	Okayasu et al., 1997, Amino Acids	Amino acid compositions of mammalian and bacterial cells after hydrolysis and derivatizations	Percentage of total amino acids
Amino Acid Composition of Whole Cells of Different Yeasts	Martini et al., 1979, Journal of Agricultural and Food Chemistry	Nitrogen components of whole cells of various yeasts	g per 100g of dry cell weight
Dietary requirements of “nutritionally non-essential amino acids” by animals and humans	Wu et al., 2013, Amino acids	amino acid composition in proteins of rat, pig, sheep and chicken	mg AA per g of protein
Comparative metabolomics in primates reveals the effects of diet and gene regulatory variation on metabolic divergence	Blekhman et al., 2014, Scientific Report	free amino acid concentration in liver tissue from three primates including human, chimpanzee and rehsus macaque	NA
Plasma and tissue free amino acid profiles and their concentration correlation in patients with lung cancer	Zhao et al., 2014, Asia Pacific Journal of Clinical Nutrition	tissue free amino acid in lung cancer and paracarcinomous tissues from patients	µmol per kg wet weight

Amino Acid Profiles of Serum and Urine in Search for Prostate Cancer Biomarkers: a Pilot Study	Dereziński et al., 2017, International Journal of Medical Sciences	concentration of free amino acids in serum samples from prostate cancer patients and healthy control subjects	μM
High-throughput quantitation of amino acids in rat and mouse biological matrices using stable isotope labeling and UPLC–MS/MS analysis	Takach et al., 2014, Journal of Chromatography B	free amino acids conecentration in liver and kidney of mouse	μM
Plasma Free Amino Acid Profiling of Five Types of Cancer Patients and Its Application for Early Detection	Miyagi et al., 2011, plos one	concentration of free amino acids in plasma samples of patients with lung cancer, gastric cancer, colorectal cancer, breast cancer, and prostate cancer and healthy controls	NA
8Amino acids in squamous cell carcinomas and adjacent normal tissues from patients with larynx and oral cavity lesions	Leme et al., 2012, clinics	content of amino acids in malignant and normal tissues from patients with squamous cell carcinoma of the larynx	μmol per g
Metabolomic profiling of lung and prostate tumor tissues by capillary electrophoresis time-of-flight mass spectrometry	Kami et al., 2013, metabolomics	free amino acid content in tumor and surrounding normal tissues from patients with lung cancer or prostate cancer	nmol per g tissue
Levels of amino acids in human hepatocellular carcinoma and adjacent liver tissue	Takashi et al., 1995, nutrition and cancer	concentration of amino acids in human hepatocellular carcinoma and adjacent hepatic tissue	mmol per 10 g wet weight
Plasma-free amino acid profiling of cervical cancer and cervical intraepithelial neoplasia patients and its application for early detection	Hasim et al., 2013, Molecular Biology Reports	concentration of free amino acids in plasma samples of patheints with cervical cancers and healthy subjects	NA
Plasma amino acid profiles of breast cancer patients early in the trajectory of the disease differ from healthy comparison groups	Barnes et al., 2014, Applied Physiology Nutrition and Metabolism	concentration of free amino acids in plasma of breast cancer patients and healthy age- and body mass index-matched females	μmol per L
Amino acid profiles among colorectal cancer patients	Bener et al., 2006, Biomedical Research	concentration of free amino acids in plasma of colorectal cancer patients and healthy control group	μmol per L

Supplementary Figures

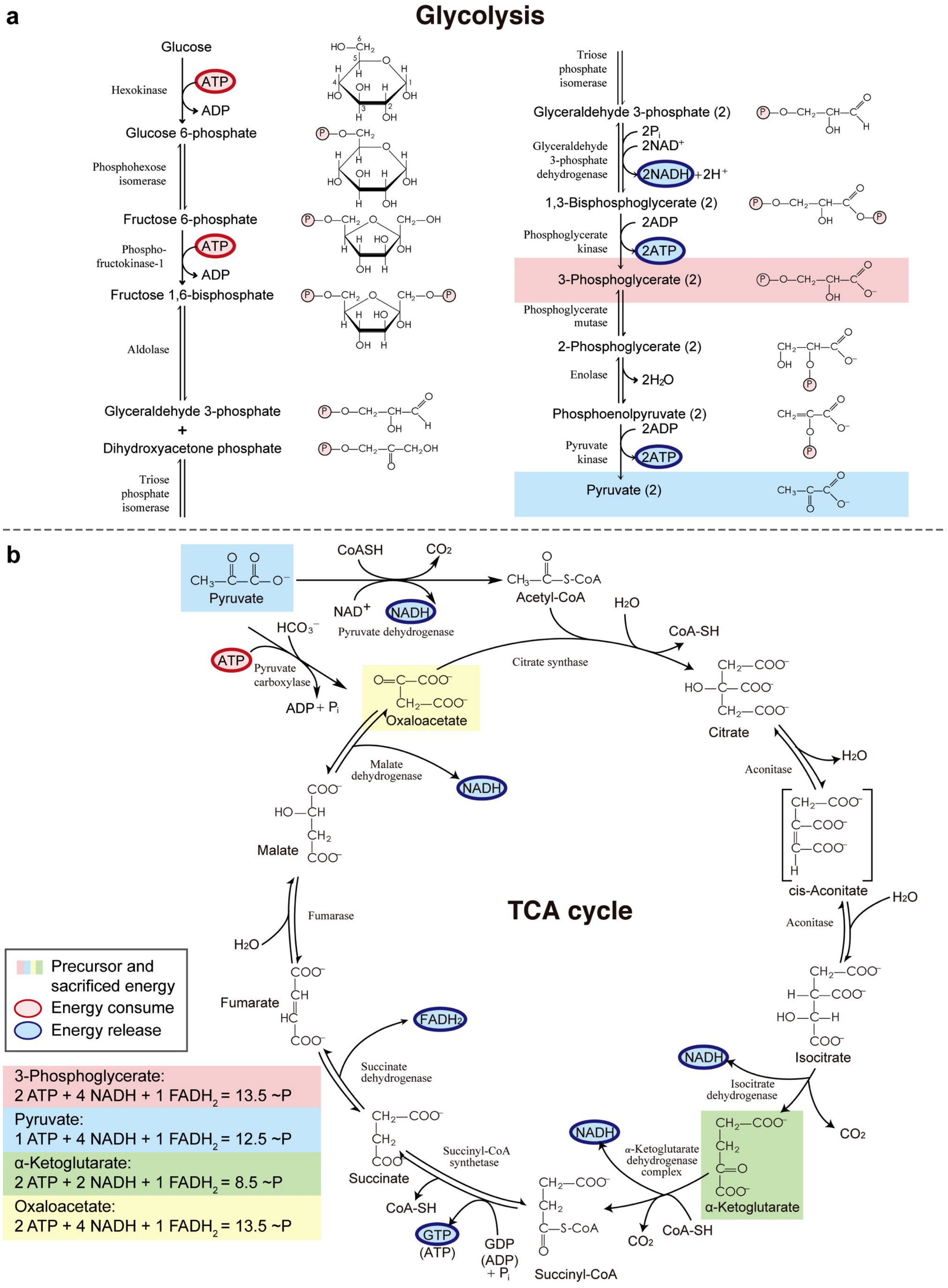

Supplementary Figure 1. The energy (~P) that would be produced if the precursors for amino acid biosynthesis (3-phosphoglycerate, pyruvate, α-ketoglutarate, and oxaloacetate) were used for energy production through glycolysis (a) and tricarboxylic acid (TCA) cycle (b) in human. The energy is stored in the form of high energy phosphate bonds (~P), such as ATP and GTP, or electron carrier NAD(P)H and $FADH_2$. The energy in reducing equivalents will be used to synthesize ATP during oxidative phosphorylation and therefore, is converted to the number of ~P with the following relationship: 1 NAD(P)H = 2.5 ~P, 1 $FADH_2$ = 1.5 ~P, and 1 ATP converted to AMP = 2 ~P. When oxaloacetate is used as a precursor for amino acid (aspartate and asparagine) biosynthesis, we assume it is synthesized from pyruvate by pyruvate carboxylase. For α-ketoglutarate, the energy required to synthesize an oxaloacetate to replenish TCA cycle from pyruvate through the anaplerotic pathway was incorporated. The energy consumed and released in the form of A(G)TP or reducing equivalents (NADH, NADPH, $FADH_2$) was highlighted with red and blue circles, respectively. The total energy would be produced from each precursor through glycolysis or TCA cycle was summarized at left bottom.

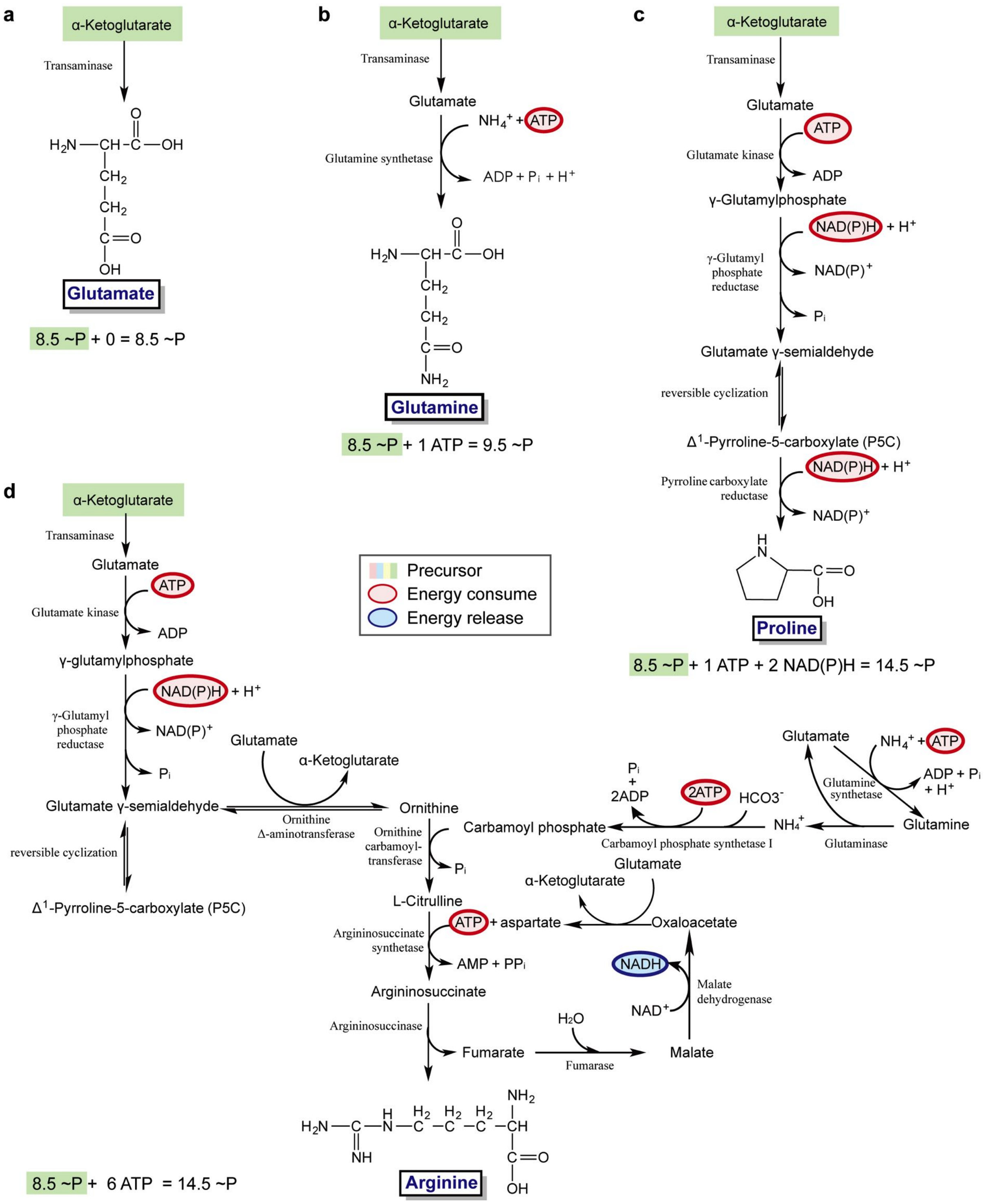

Supplementary Figure 2. The energy (~P) required in enzymatic reactions during biosynthesis of glutamate (a), glutamine (b), proline (c), and arginine (d) in human cells.

The energy consumed was highlighted with red circles and energy produced with blue circles. The total biosynthetic cost (~P) of each amino acid is the sum of energy (~P) would be produced if the precursors were used for energy production (colored box) and the energy (~P) required for converting precursors to AA. The cost is summarized below the biosynthetic pathway of each amino acid.

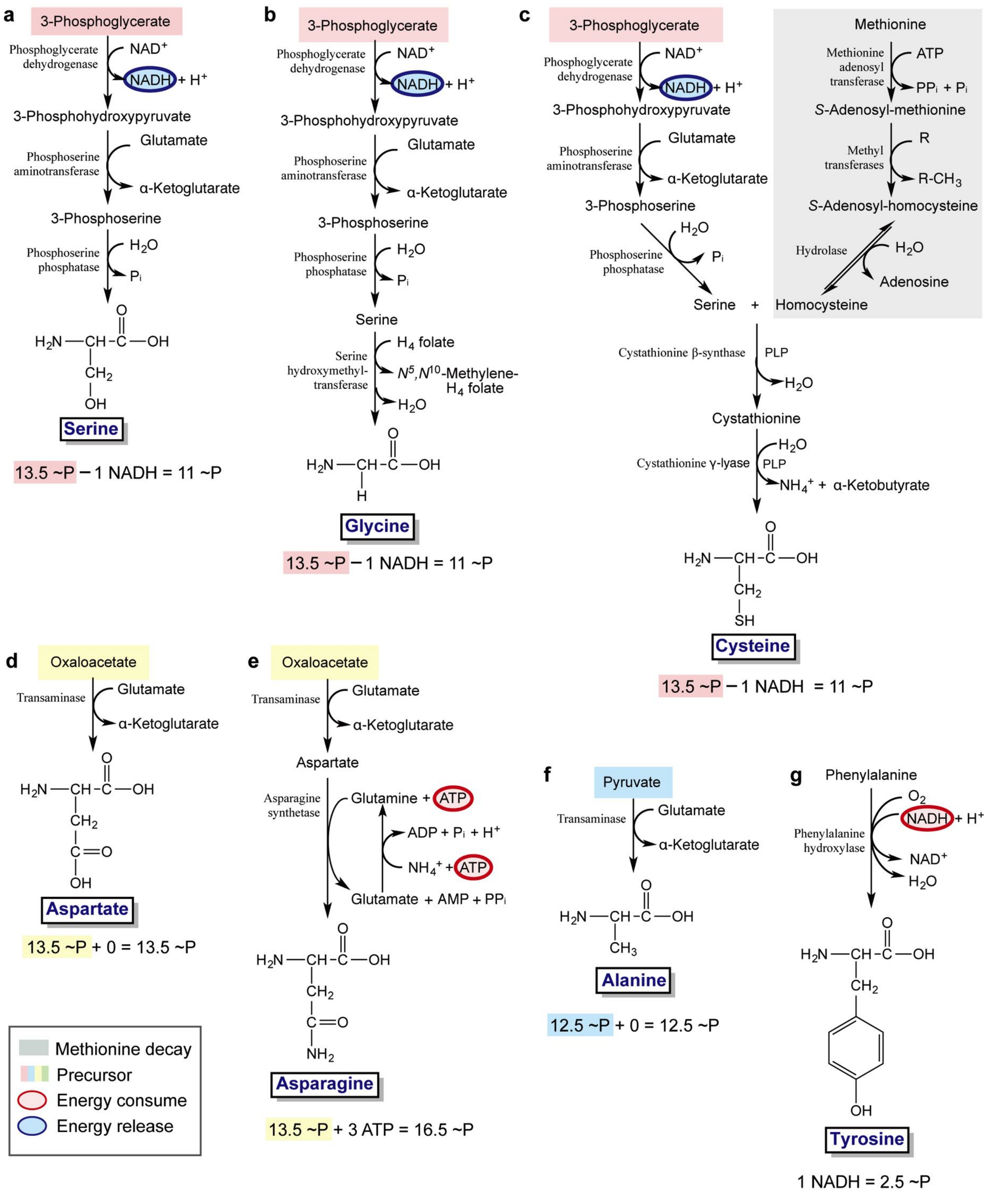

Supplementary Figure 3. The energy (~P) required in enzymatic reactions during biosynthesis of serine (a), glycine (b), cysteine (d), aspartate (d), asparagine (e), alanine (f) and tyrosine (g) in human cells.

Note cysteine is synthesized from serine and homocysteine that is produced during catabolism of methionine. Tyrosine is derived by hydroxylation of phenylalanine. Since both methionine and phenylalanine are EAAs, the biosynthetic cost in the autotrophs was not considered here. We only calculated the cost (~P) consumed in converting homocysteine to cysteine, and the cost of converting phenylalanine to tyrosine.

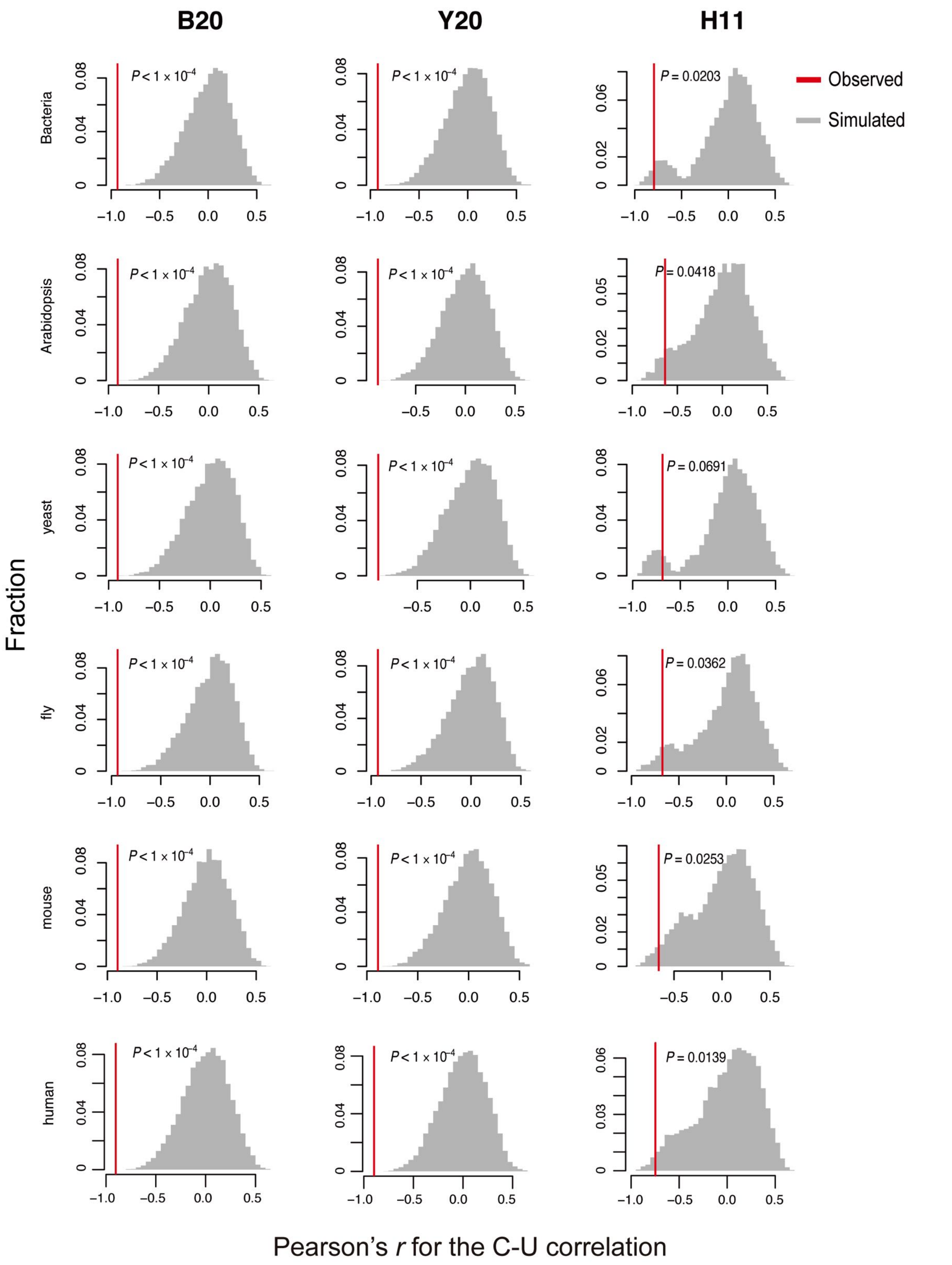

Supplementary Figure 4. The observed (red line) and simulated (grey) Pearson's *r* for the C-U correlation in six model organisms (*E. coli*, *A. thaliana*, yeast, fly, mouse, and human).

The permutation analysis was performed by randomly shuffling the costs of AAs for 10,000 times and conducting the correlation analysis. Each of the three cost metrics (B20, Y20 or H11) was used in the analysis. In each panel, the *P* value indicates the fraction of simulations yielding smaller Pearson's *r* than the observed one (red line).

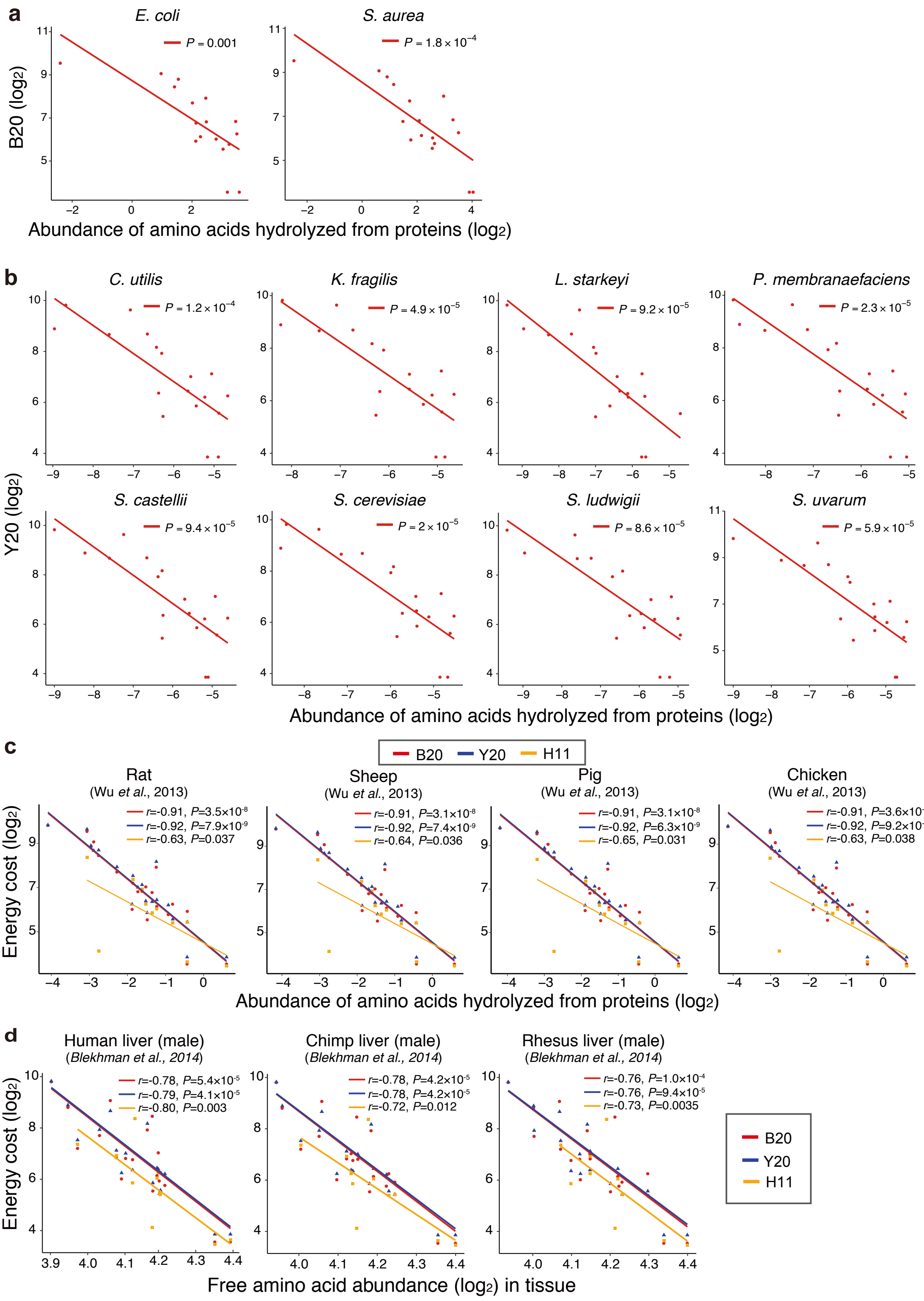

Supplementary Figure 5. Relationship between biosynthetic cost of AAs and abundances of AAs hydrolyzed from proteins or free AAs in animal tissues.

(a) The relationship between biosynthetic cost (B20, log_2 transformed) and the experimentally measured abundance of AAs (log_2 transformed) hydrolyzed from proteins in whole cells of two bacterial species (Okayasu et al., 1997). Pearson's r and *P* value were calculated and displayed in the top of each panel.

(b) The relationship between biosynthetic cost (Y20, log_2 transformed) and the experimentally measured abundance of AAs (log_2 transformed) hydrolyzed from proteins in whole cells of various yeast species (Martini et al., 1979). Pearson's *r* and *P*-value were calculated and displayed in the top of each panel.

(c) The relationship between biosynthetic cost (B20, Y20, or H11, log_2 transformed) and the experimentally measured abundance of AAs (log_2 transformed) hydrolyzed from proteins in whole bodies of Rat, Sheep, Pig, and Chicken (Wu et al., 2013).

(d) The relationship between biosynthetic cost of AAs (B20, Y20, H11) and the experimentally measured *in vivo* concentration of free AAs in livers of males of humans, chimpanzee and rhesus monkey (Blekhman, et al. 2014).

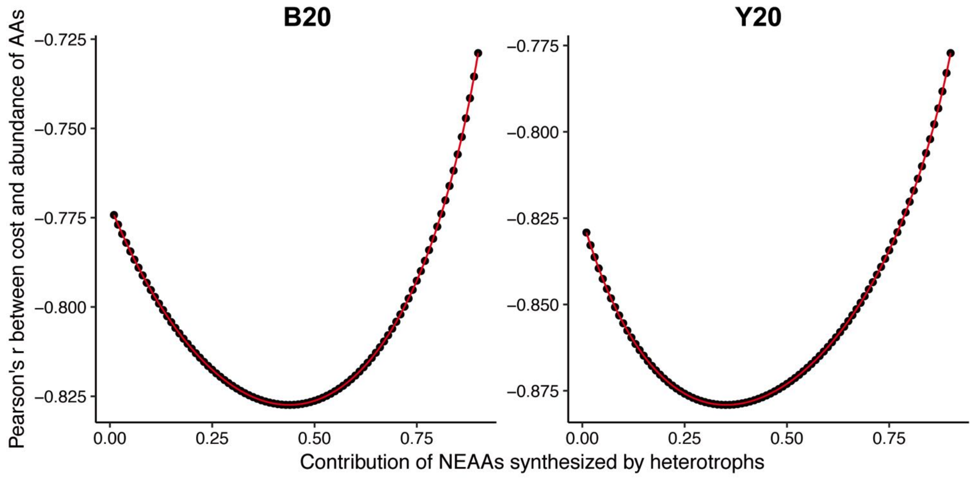

Supplementary Figure 6. The relationship between cost of AAs in autotrophs and their relative abundance in the simulated free AA pool of human cells.

Human free AA pool is composed of two sources of AAs: NEAAs synthesized by human cells or other heterotrophs and all 20 kinds of AAs taken from autotrophs. For AAs from autotrophs, their abundance is in proportion to their composition in the proteome of yeast (Martini et al., 1979) or *E. coli* (Okayasu et al., 1997). NEAAs from heterotrophs are assumed to be in proportion to their abundance in human serum. The X-axis is the proportion of AAs contributed by NEAAs from heterotrophs. The Y-axis is the Pearson's *r* between abundance of the 20 AAs in the simulated free AA pool and their cost in autotrophs.

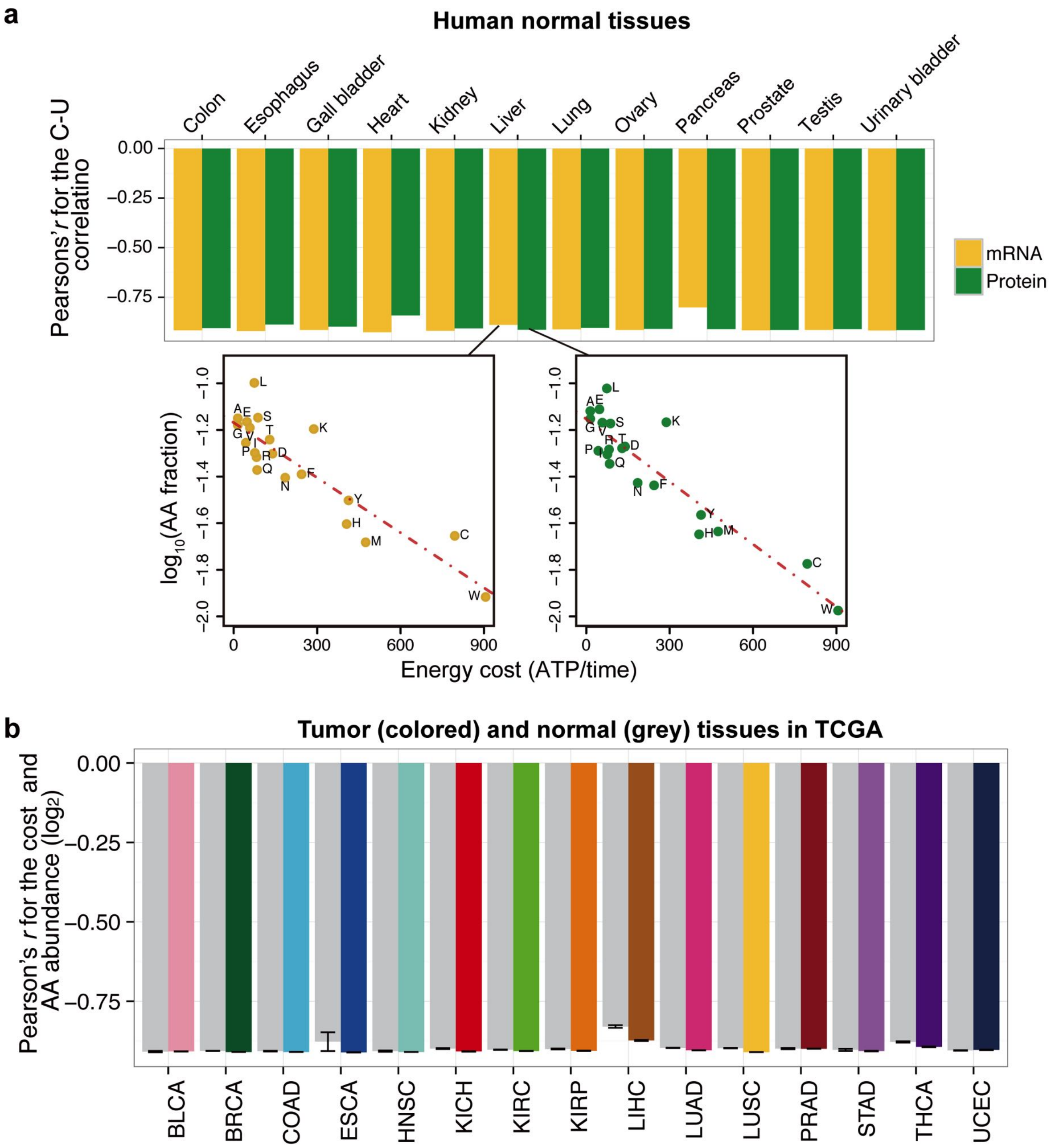

Supplementary Figure 7. Impact of biosynthetic costs of AAs (Y20) on the usage of AAs in the proteomes of normal and cancer tissues.

(a) Pearson's *r* for the C-U correlation after considering gene expression levels in 12 human normal tissues that have both mRNA-Seq and proteomic data available. Two representative correlations (mRNA-Seq and proteome of liver) are magnified for more detail.

(b) Pearson's *r* for the C-U correlation after considering gene expression levels in different cancer (colored) and normal (grey) tissues using TCGA mRNA-Seq data. Error bars indicate the 95% confidence intervals of *r*.

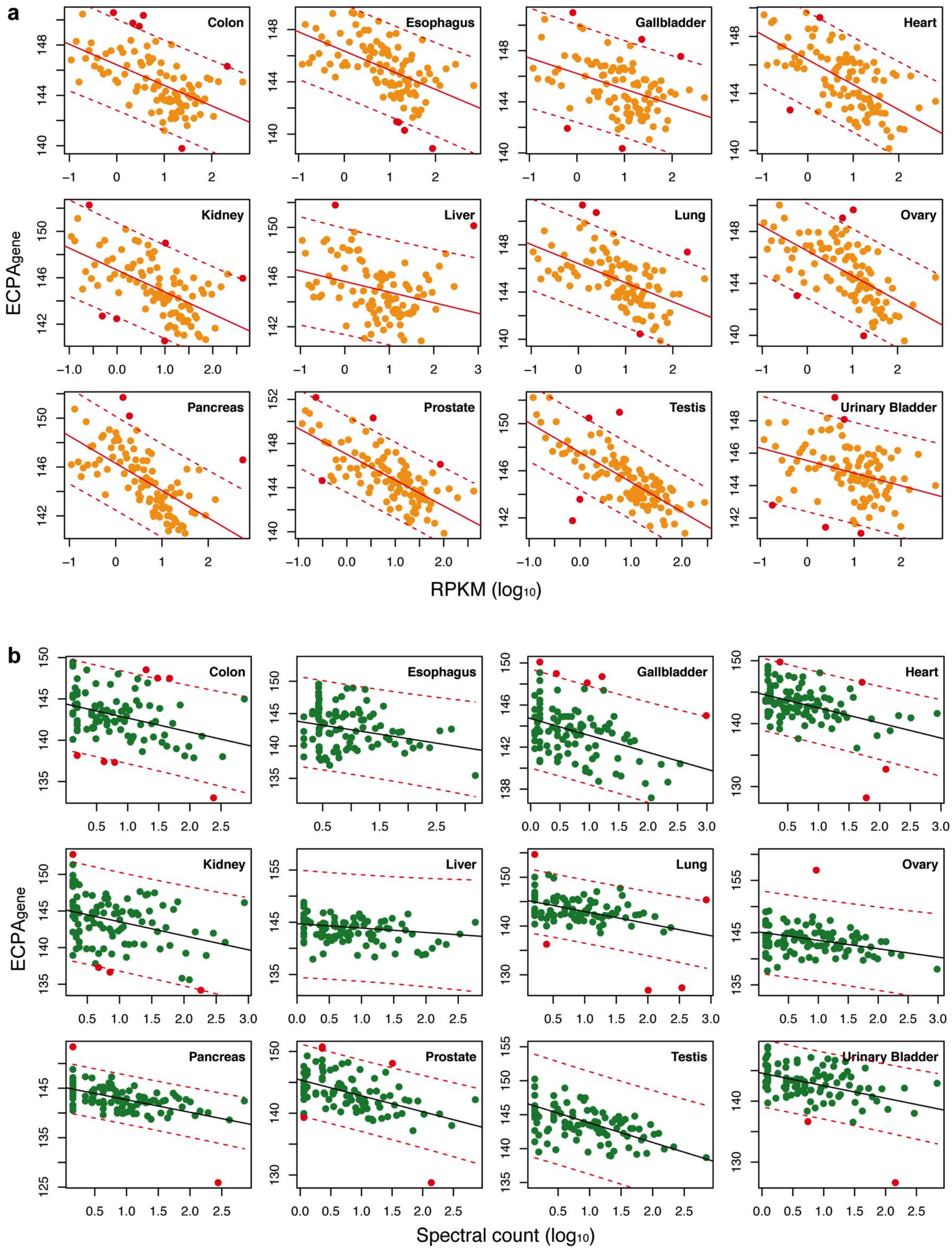

Supplementary Figure 8. The relationship between ECPA$_{gene}$ and expression levels of genes in 12 normal human tissues with both RNA-Seq and proteomic data available.

For each tissue, genes were divided into 100 bins based on increased expression levels (RPKM for RNA-Seq and spectral count for proteomic data). Median expression level (log_{10}) and median ECPA$_{gene}$ in each bin were displayed in the plot. In each panel, genes with higher expression level have significantly lower ECPA$_{gene}$ (Spearman's correlation, $P < 0.05$). The red dashed lines indicate 95% prediction interval. **(a)** RNA-Seq data. **(b)** Proteomic data.

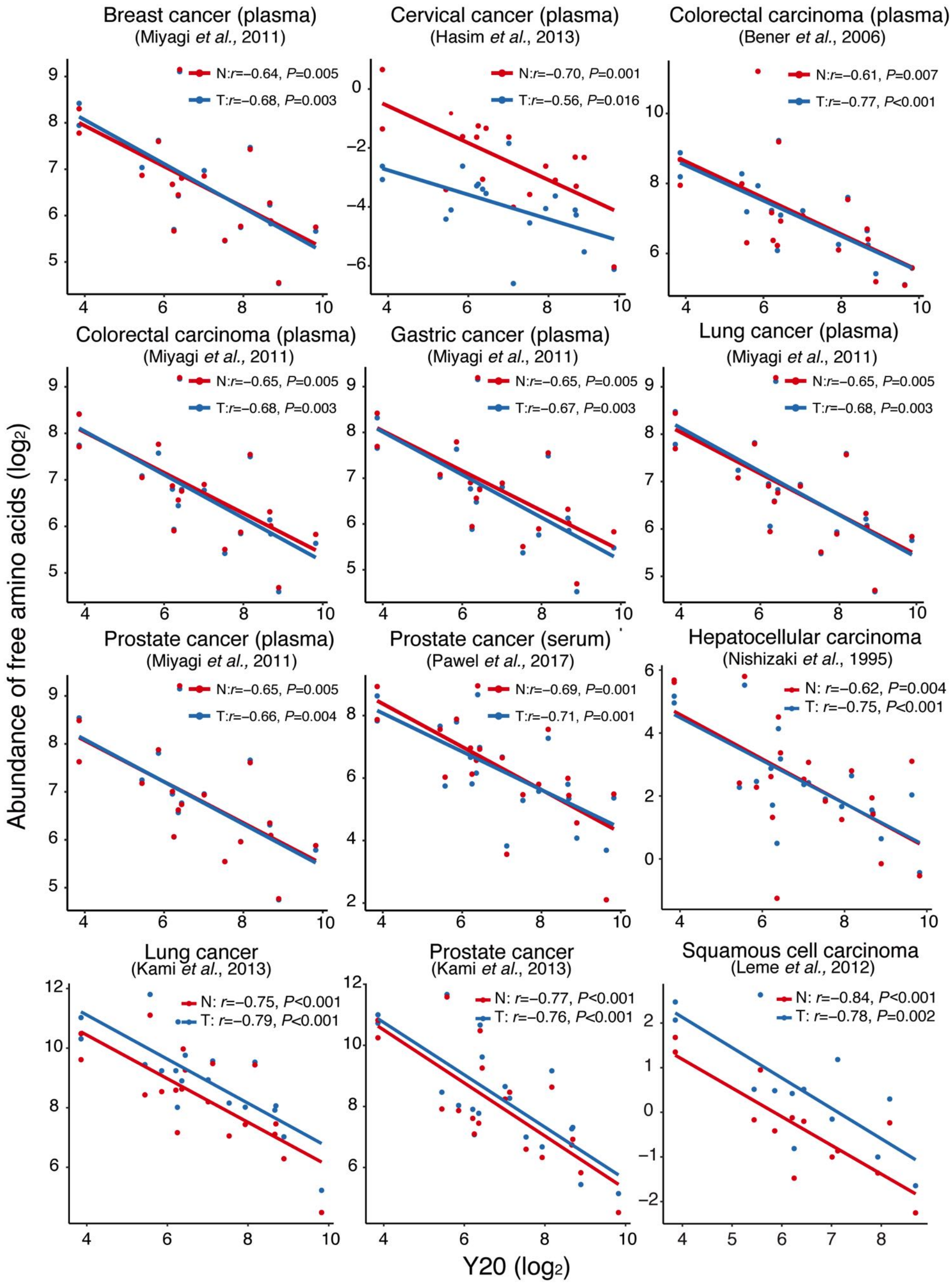

Supplementary Figure 9. The relationship between biosynthetic cost of amino acids (Y20) and the *in vivo* concentration of free AAs (log_2 transformed) in tumorous (T) or normal (N) samples. Information of the samples are as the following: plasma of heathy controls and patients with breast cancer (Miyagi et al., 2011), cervical cancer (Hasim et al., 2013), colorectal carcinoma (Bener et al., 2006; Miyagi et al., 2011), gastric cancer (Miyagi et al., 2011), lung cancer (Miyagi et al., 2011), prostate cancer (Miyagi et al., 2011), serum of healthy controls and patients with prostate cancer (Dereziński et al., 2017), tumor and normal samples from liver of patients with hepatocellular carcinoma (Nishizaki et al., 1995), lung of patients with lung cancer (Kami et al., 2013), prostate of patients with prostate cancer (Kami et al., 2013), and skin of patients with squamous cell carcinoma (Leme Ide et al., 2012). Pearson's correlations were calculated for both tumor and normal samples in each study.

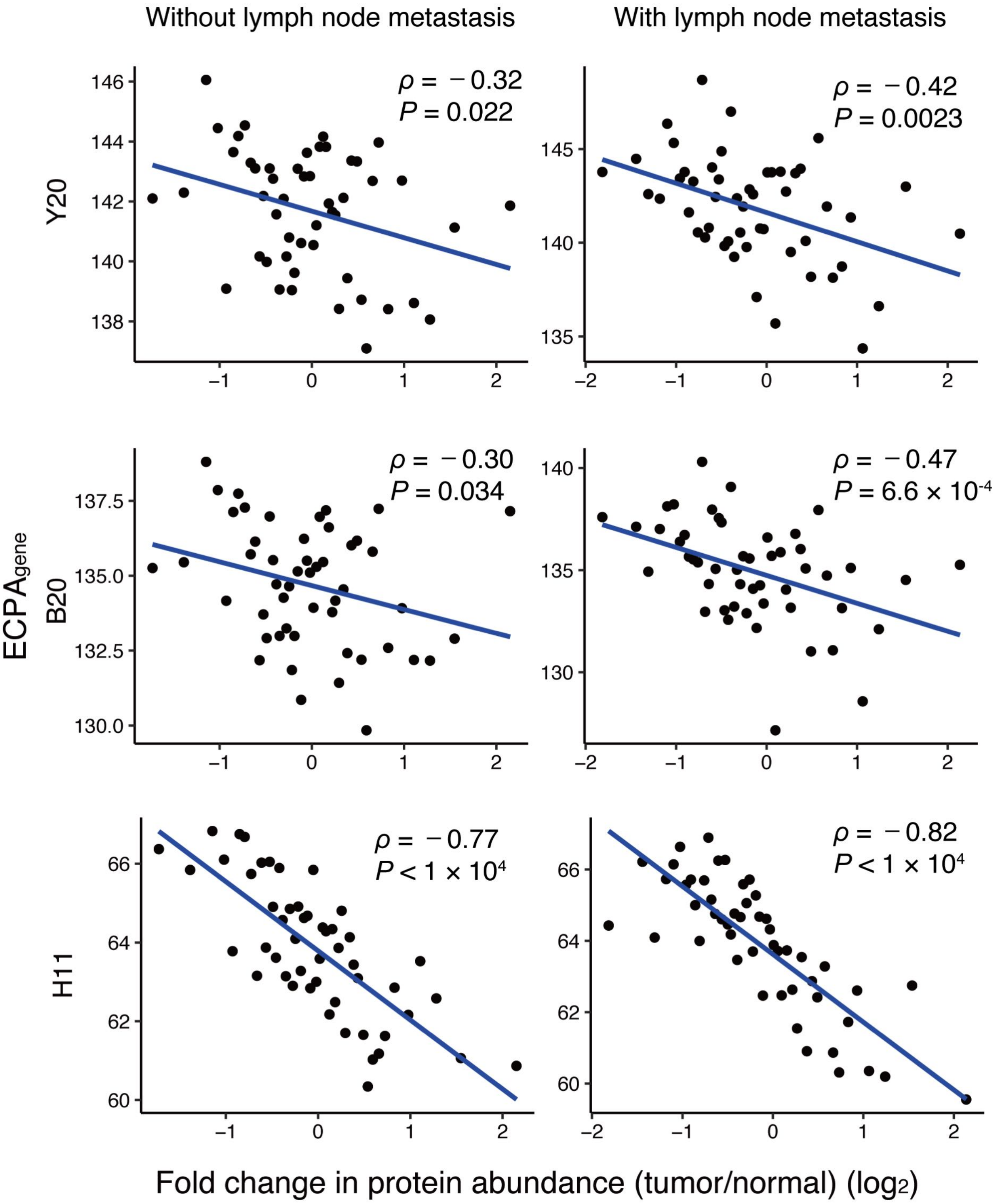

Supplementary Figure 10. Negative correlation between the $\log_2$(fold change) of protein abundance in tumor relative to normal tissues (*x-axis*) in breast cancer without (right) or with (left) lymph node metastasis and the $ECPA_{gene}$ calculated with cost metric Y20 (top), B20 (middle) and H11 (bottom), respectively.

The genes were grouped into 50 bins based on increasing $\log_2$(fold change), and medians of $ECPA_{gene}$ and $\log_2$(fold change) for each bin are displayed in the plot and used for correlation analysis.

NO.	Cancer type	Name	Number of samples		Significance of Cancer-Normal Difference (15)	Significance of ECPA-Stage correlation (20)	Significance of ECPA-Survival correlation (17)	
			Tumor	Nomal			Log-Rank	Cox
1	ACC	Adrenocortical carcinoma	79	/	/	*	*	**
2	BLCA	Bladder Urothelial Carcinoma	408	19		**	*	**
3	BRCA	Breast invasive carcinoma	1097	114	***	*		
4	CHOL	Cholangiocarcinoma	36	9	/	*		
5	COAD	Colon adenocarcinoma	286	41	***	***	*	
6	ESCA	Esophageal carcinoma	184	11	*			
7	GBM	Glioblastoma multiforme	154	5	/	/		
8	HNSC	Head and Neck squamous cell carcinoma	520	44				
9	KICH	Kidney Chromophobe	66	25	***	*		
10	KIRC	Kidney renal clear cell carcinoma	533	72	***		**	***
11	KIRP	Kidney renal papillary cell carcinoma	290	32	***			
12	LAML	Acute Myeloid Leukemia	179	/	/	/		*
13	LIHC	Liver hepatocellular carcinoma	371	50	***	***	***	***
14	LUAD	Lung adenocarcinoma	515	59	***	**	*	*
15	LUSC	Lung squamous cell carcinoma	502	51	***			
16	MESO	Mesothelioma	87	/	/		***	***
17	OV	Ovarian serous cystadenocarcinoma	304	/	/	/		*
18	PAAD	Pancreatic adenocarcinoma	178	4	/			
19	PRAD	Prostate adenocarcinoma	497	52		/	*	*
20	READ	Rectum adenocarcinoma	94	10	*	***		
21	SARC	Sarcoma	259	2	/	/	**	
22	SKCM	Skin Cutaneous Melanoma	104	1	/	/	***	***
23	STAD	Stomach adenocarcinoma	380	37	*			
24	TGCT	Testicular Germ Cell Tumors	150	/	/			
25	THCA	Thyroid carcinoma	505	59	***	*		
26	UCEC	Uterine Corpus Endometrial Carcinoma	176	24		/		
27	UVM	Uveal Melanoma	80	/	/			
28	PCPG	Pheochromocytoma and Paraganglioma	179	3	/	/	*	
29	LGG	Brain Lower Grade Glioma	516	/	/	/		*
30	CESC	Cervical squamous cell carcinoma and endocervical adenocarcinoma	305	3	/	/		
31	DLBC	Lymphoid Neoplasm Diffuse Large B-cell Lymphoma	48	/	/	/		
32	THYM	Thymoma	120	2	/	/		
33	UCS	Uterine Carcinosarcoma	57	/	/	/		

"*": *P*-value < 0.05, "**": *P*-value < 0.01, "***": *P*-value < 0.001.

Supplementary Figure 11. Summary of clinically relevant patterns of 33 TCGA cancer types.

For each cancer type, the numbers of tumor and normal samples, the statistical difference in $ECPA_{cell}$ between tumor and normal samples, the association of $ECPA_{cell}$ with cancer stage, and the association between $ECPA_{cell}$ and patient survival time (log-rank and Cox) are given. Negative associations are indicated by blue and positive associations are indicated by red. Cancer types without required information are denoted with "/". Cancer types without sufficient data are denoted with light colors.

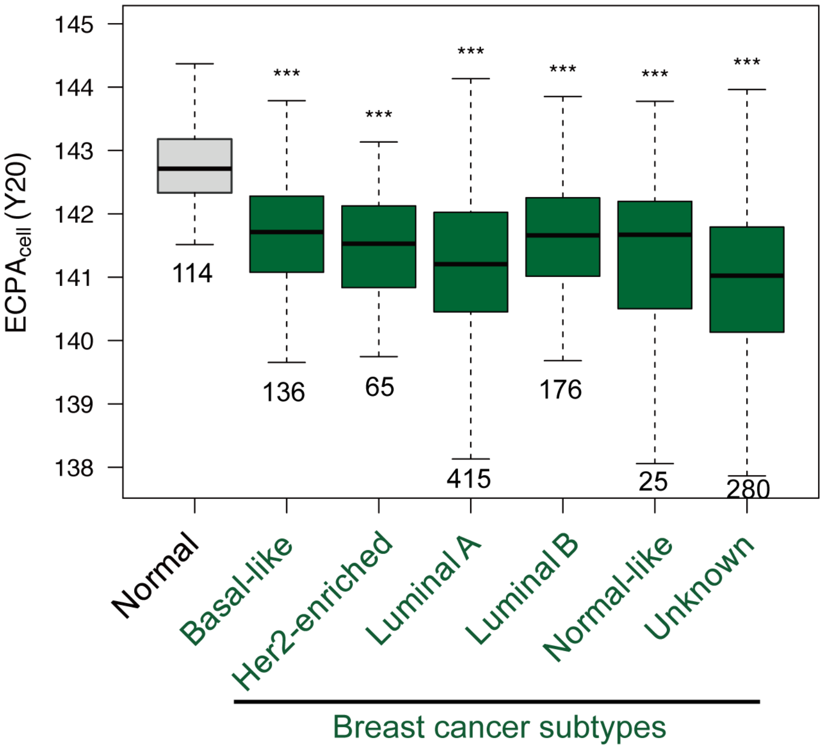

Supplementary Figure 12. The ECPA$_{cell}$ (Y20) for different subtypes of breast cancer and matched normal samples.

The PAM50 molecular subtypes of breast cancer samples were obtained from a recent study (Ciriello, et al., 2015). The number of samples for each subtype is displayed below the boxes. Differences in ECPA$_{cell}$ between each subtype and normal samples are compared with Wilcoxon rank-sum test (***, $P < 0.001$). Center line, median; box limits, upper and lower quartiles; whiskers, 1.5 times the interquartile range.

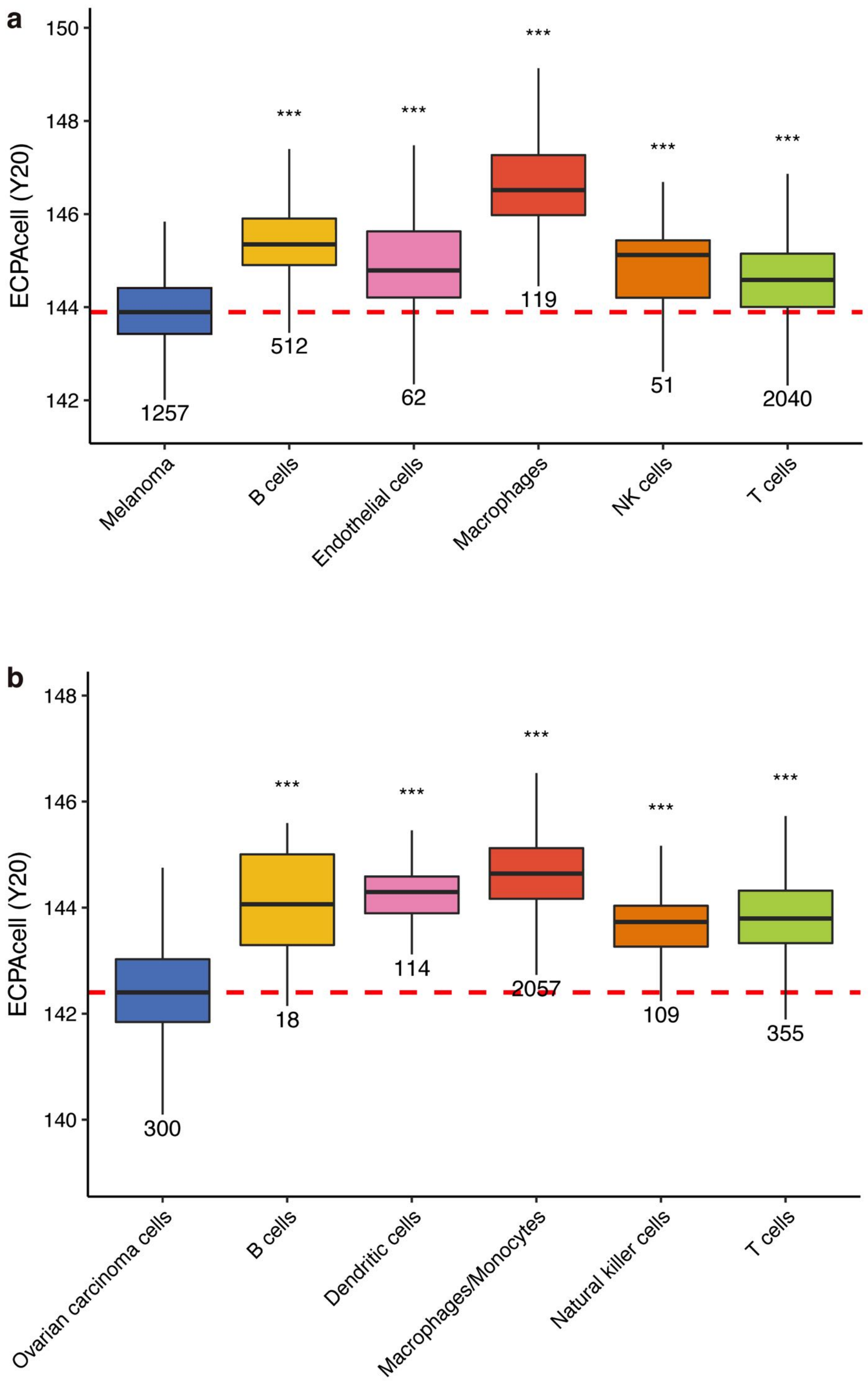

Supplementary Figure 13. The $\text{ECPA}_{\text{cell}}$ (Y20) for different types of cells in the tumor microenvironment.

The number of cells belonging to each cell type is displayed below the boxes. The differences in $\text{ECPA}_{\text{cell}}$ between malignant cancer cells and immune or stromal cells are compared with Wilcoxon rank-sum tests (***, $P < 0.001$). The red dashed line indicates median $\text{ECPA}_{\text{cell}}$ of cancer cells. **(a)** The results for melanoma dataset (Tirosh et al., 2016). **(b)** The results for ovarian cancer ascites dataset (Schelker et al., 2017). Center line, median; box limits, upper and lower quartiles; whiskers, 1.5 times the interquartile range.

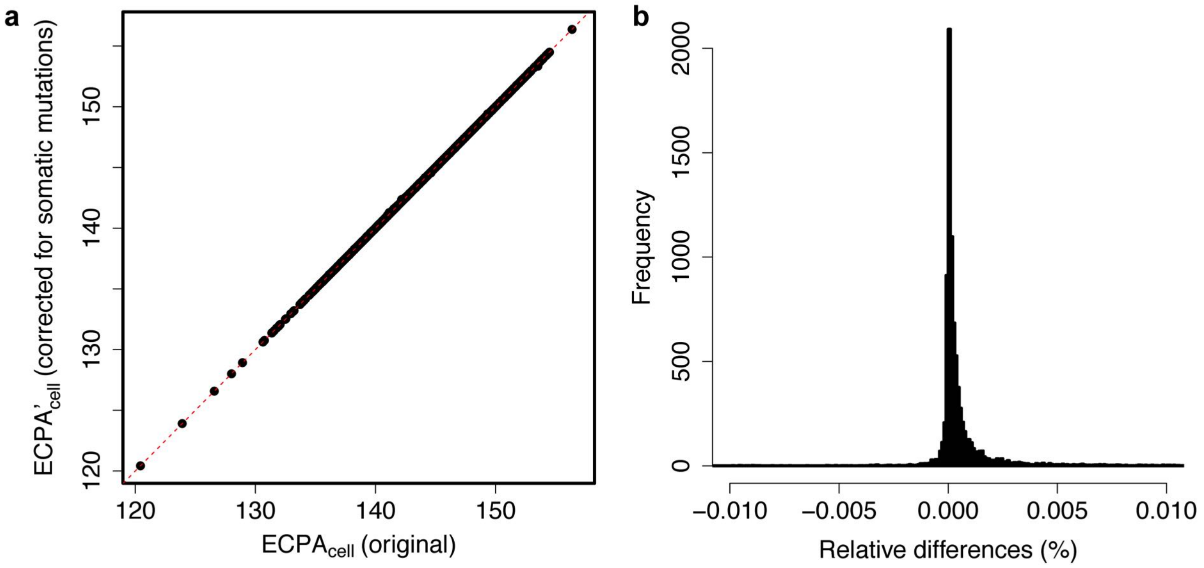

Supplementary Figure 14. Somatic mutations have negligible influence on ECPAcell values.

(a) The relationship between $ECPA_{cell}$ after correcting for somatic mutations ($ECPA_{cell}'$, *y*-axis) and the original $ECPA_{cell}$ (*x*-axis) for all tumor samples with somatic mutation data available.

(b) The distribution of the relative differences between $ECPA_{cell}$ and $ECPA_{cell}'$.

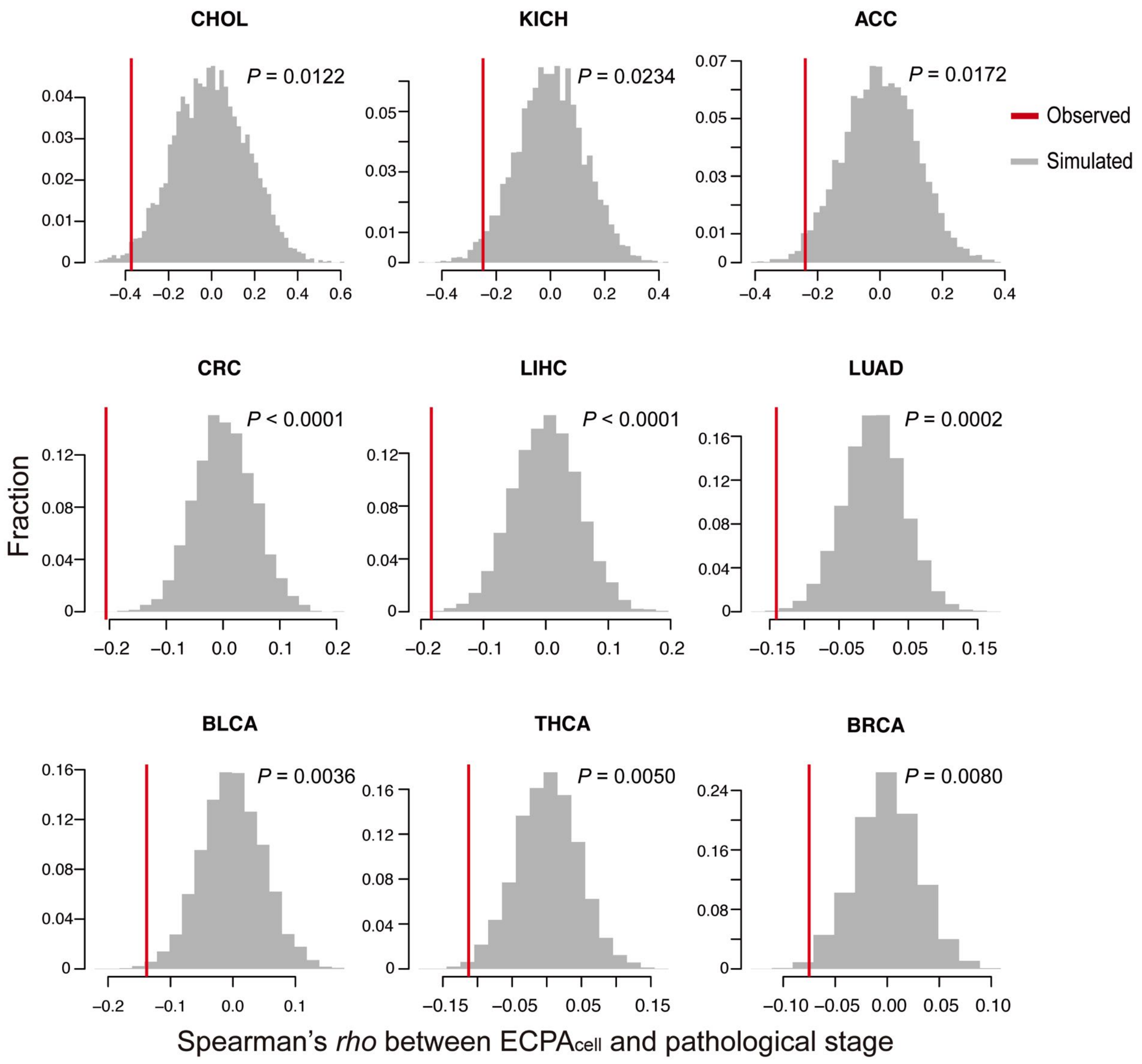

Supplementary Figure 15. The observed (red line) and simulated (grey) Spearman's *rho* between the pathological stage of tumors and $ECPA_{cell}$ (Y20) in 9 cancer types.

For each cancer type, the permutation analysis was performed by randomly shuffling the $ECPA_{cell}$ of samples for 5,000 times and conducting the correlation analysis. The *P* value in each cancer type was obtained by comparing the fraction of simulations that yielded a smaller Spearman's *rho* compared to the observed *rho*.

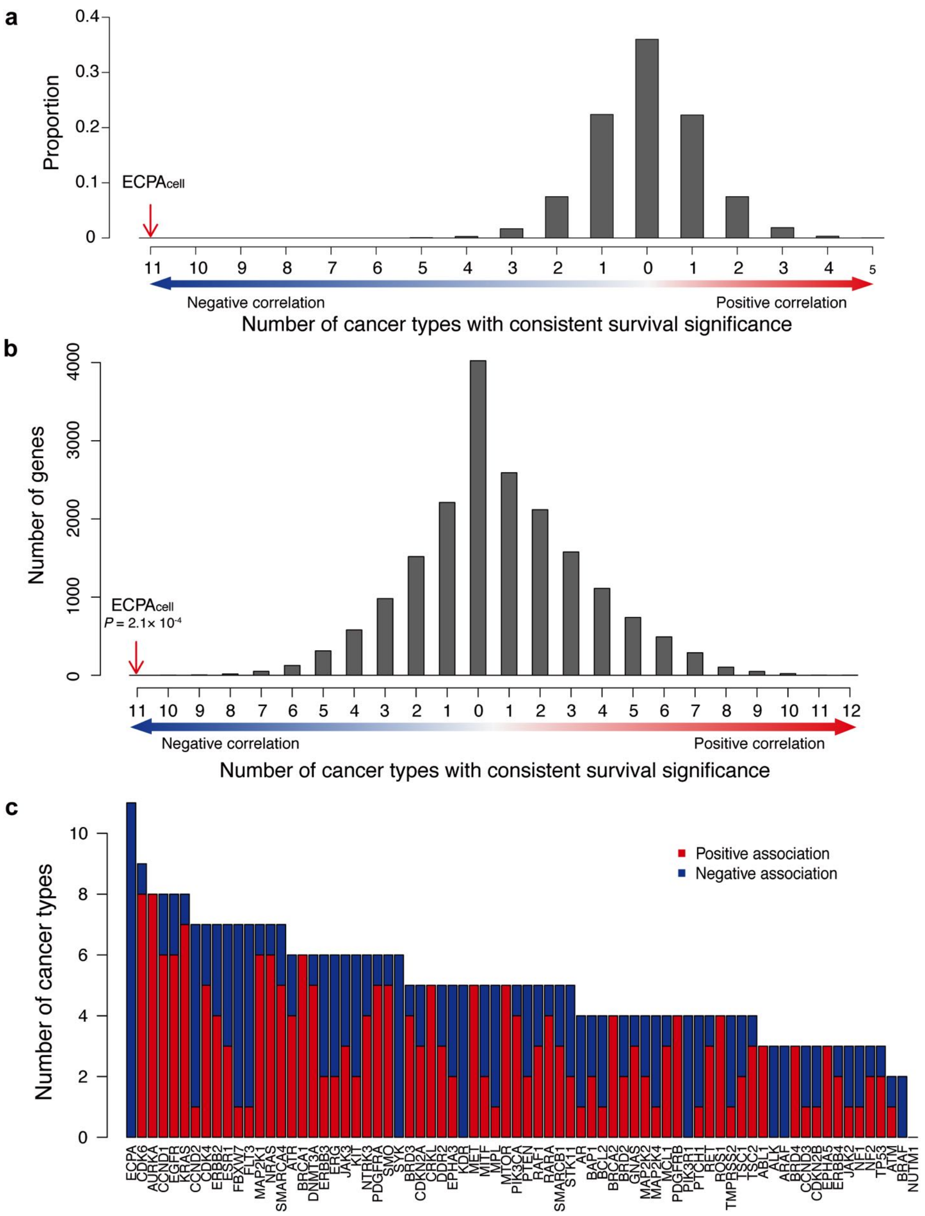

Supplementary Figure 16. Associations of ECPA$_{cell}$ with patient prognosis across cancer types.

(a) The observed number of cancer types with a consistent survival correlation by ECPA$_{cell}$ (arrow) compared to random expectations by permuting ECPA$_{cell}$ of cancer samples (5,000 times). The x-axis is the number of cancer types with a consistent survival correlation in the simulations (negatively or positively correlated) and the y-axis is the fraction of simulations obtained for that particular number of cancer types.

(b) The number of genes (y-axis) whose expression levels show a consistent association in the survival analyses of different cancer types compared to ECPA$_{cell}$.

(c) The number of cancer types in which the survival time is significantly correlated with ECPA$_{cell}$ or a set of known cancer therapeutic targets or biomarker genes. Different colors represent different correlation directions.

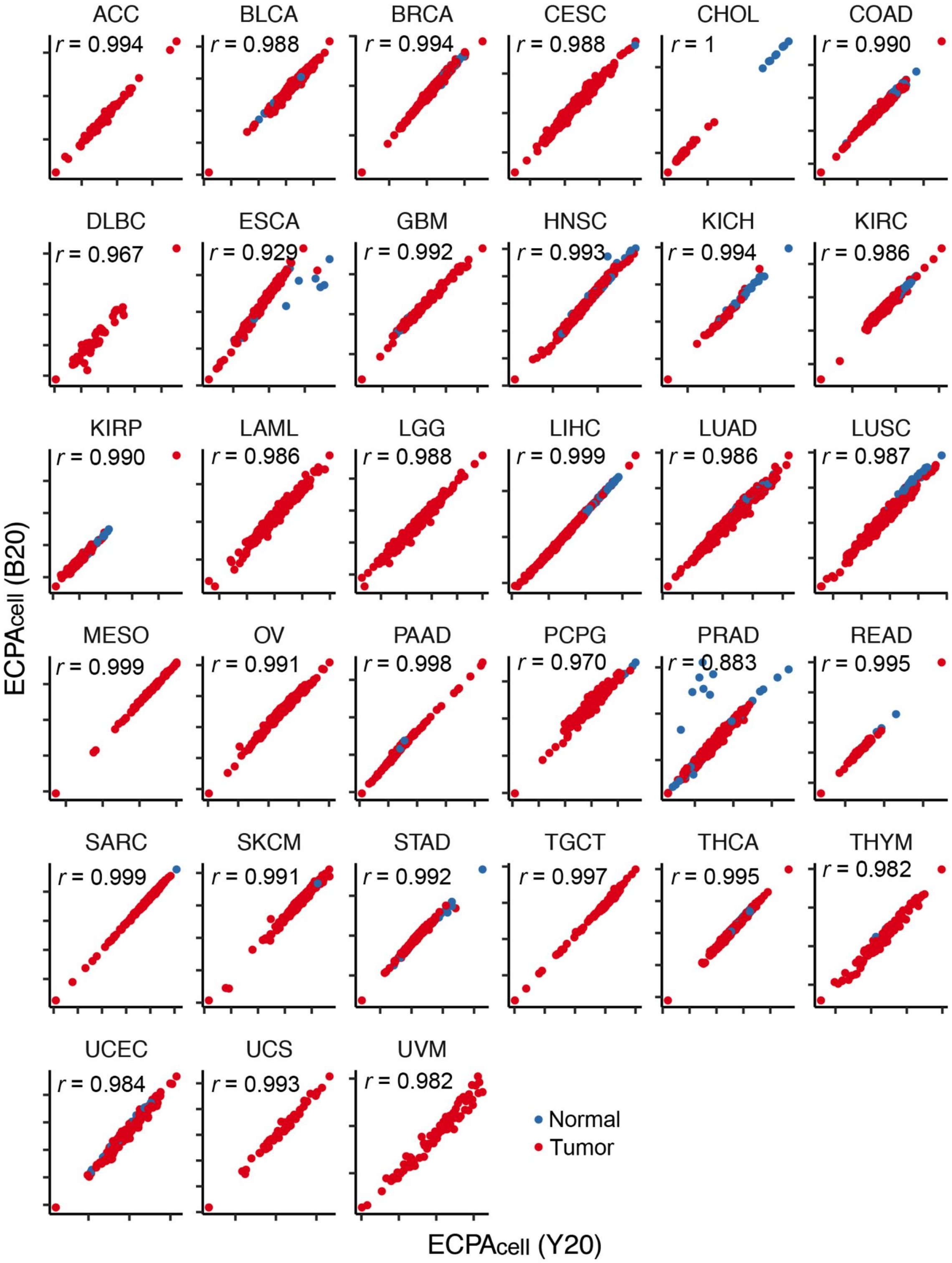

Supplementary Figure 17. The relationship between ECPA$_{cell}$ calculated with Y20 (*x-axis*) and the corresponding value calculated with B20 (*y-axis*).

Tumor samples and normal tissue samples were denoted with red and blue dots, respectively. Pearson's *r* was calculated and displayed in the top left of each panel. $P < 10^{-5}$ in all the analysis.

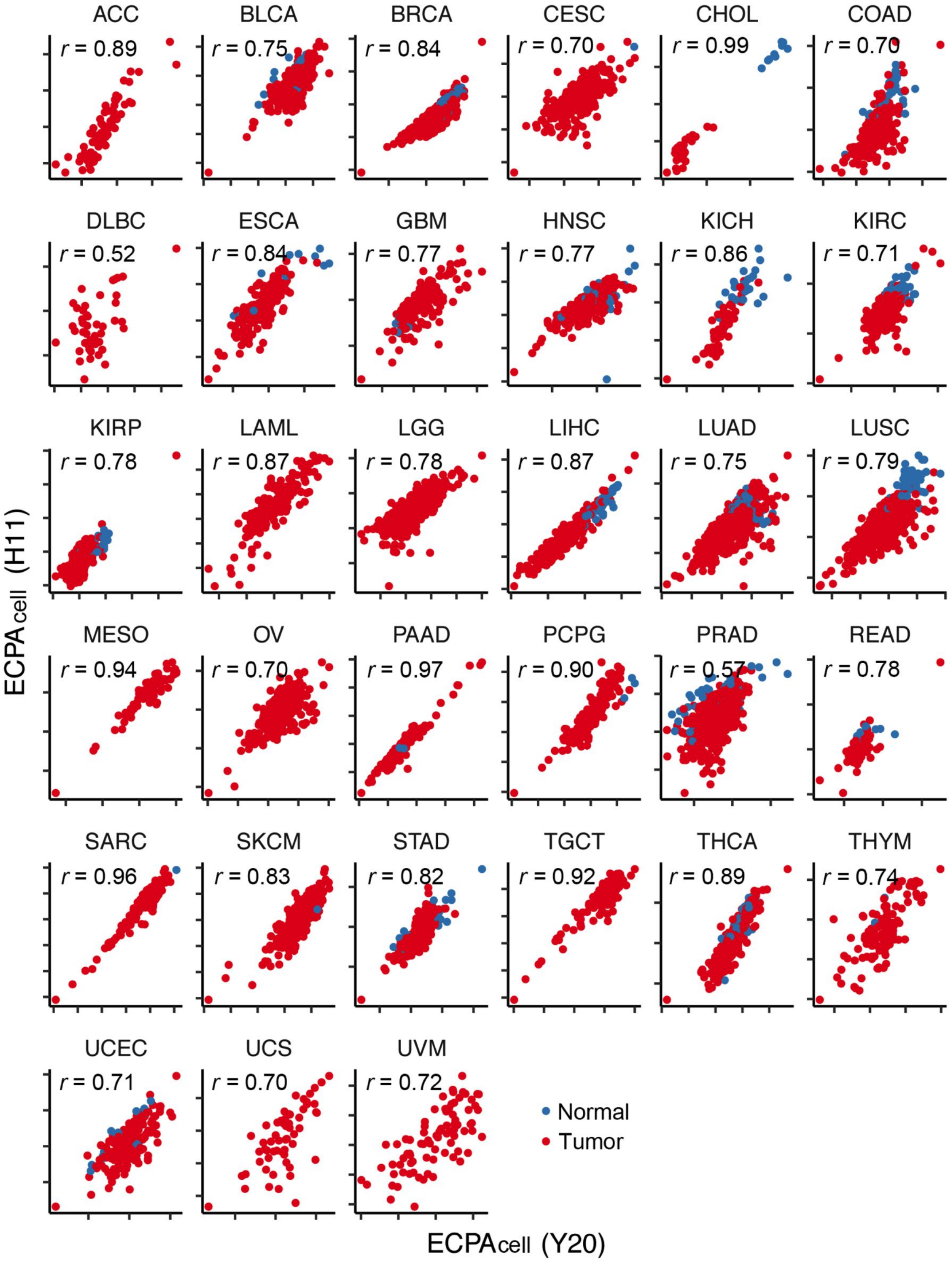

Supplementary Figure 18. The relationship between $\text{ECPA}_{\text{cell}}$ calculated with Y20 (*x-axis*) and the corresponding value calculated with H11 (*y-axis*).

Tumor samples and normal tissue samples were denoted with red and blue dots, respectively. Pearson's *r* was calculated and displayed in the top left of each panel. $P < 10^{-5}$ in all the analysis.

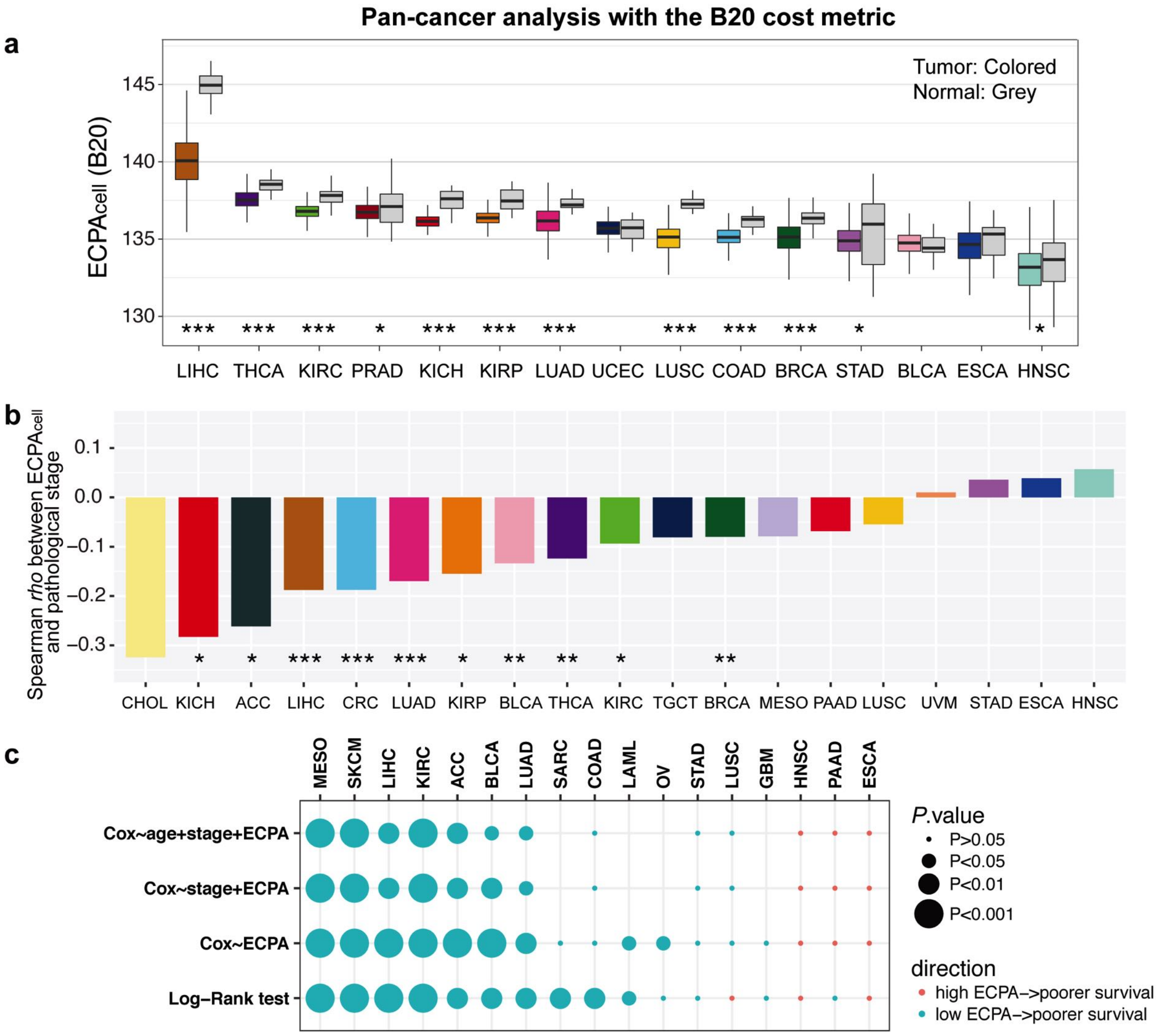

Supplementary Figure 19. Clinically relevant patterns of ECPA$_{cell}$ across cancer types using ECPA$_{cell}$ calculated with B20 metric.

(a) ECPA$_{cell}$ of tumor samples is significantly lower than that of matched normal tissue samples in 12 out of 15 cancer types for which RNA-Seq data of >10 normal samples were available. Wilcoxon rank-sum tests are performed to compared the difference in ECPA$_{cell}$ between tumor samples and normal tissue samples (***, $P < 0.001$; **, $P < 0.01$; *, $P < 0.05$). Center line, median; box limits, upper and lower quartiles; whiskers, 1.5 times the interquartile range.

(b) ECPA$_{cell}$ is significantly negatively correlated with the pathologic stage for patients in 10 out of 19 cancer types that have pathological stage data available (***, $P < 0.001$; **, $P < 0.01$; *, $P < 0.05$). Colon and rectum adenocarcinoma are merged as colorectal carcinoma (CRC) in the analysis.

(c) ECPA$_{cell}$ is significantly associated with the patients' survival times in 11 out of 17 cancer types that have ≥75 cases and ≥25% events. Circle size indicates significance of the correlation; color indicates direction of the correlation. The log-rank tests or Cox proportional hazards model was used in the analysis.

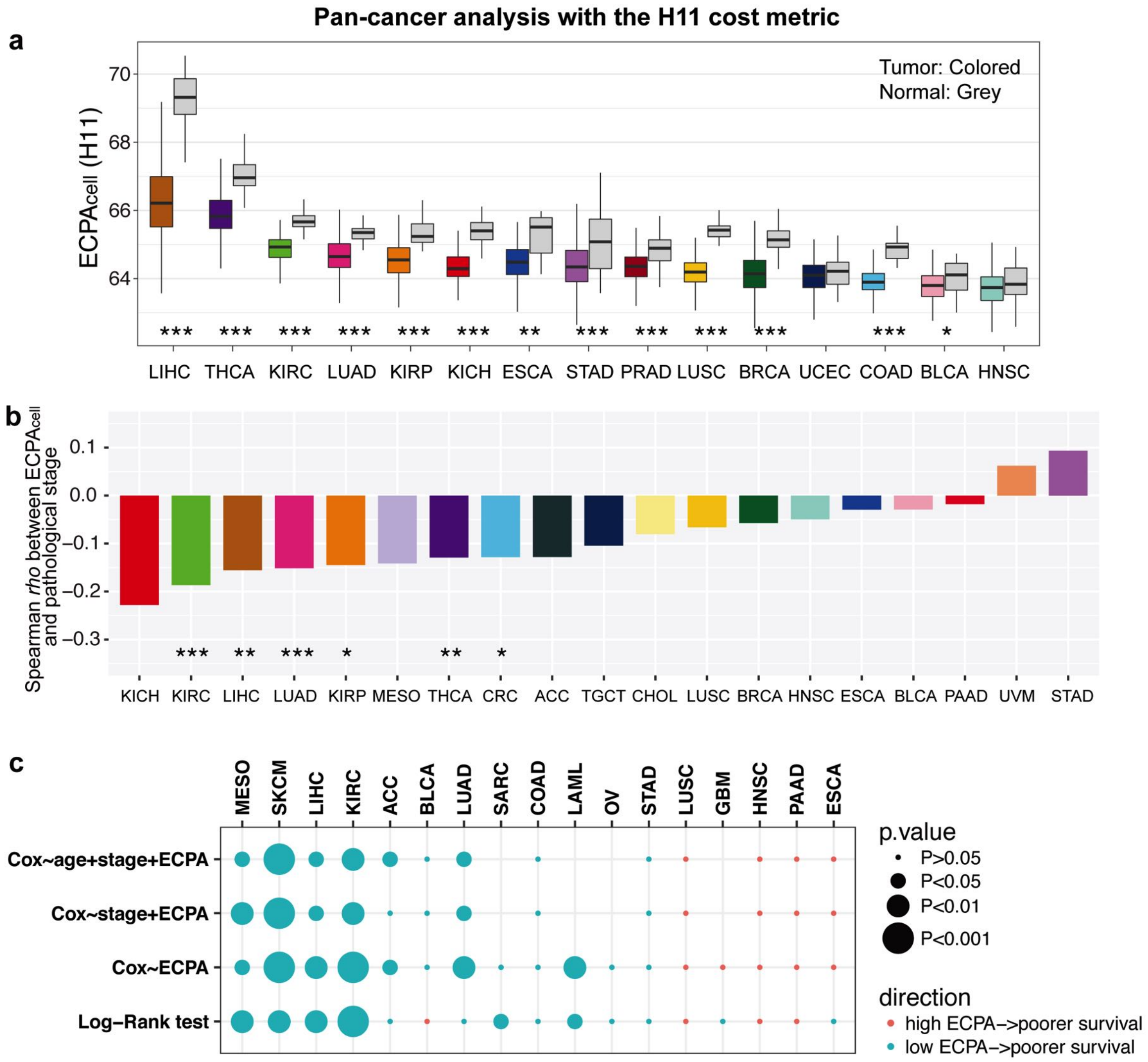

Supplementary Figure 20. Clinically relevant patterns of $ECPA_{cell}$ across cancer types using $ECPA_{cell}$ calculated with H11 metric.

(a) $ECPA_{cell}$ of tumor samples is significantly lower than that of matched normal tissue samples in 13 out of 15 cancer types for which RNA-Seq data of >10 normal samples were available. Wilcoxon rank-sum tests are performed to compared the difference in $ECPA_{cell}$ between tumor samples and normal tissue samples (***, $P < 0.001$; **, $P < 0.01$; *, $P < 0.05$). Center line, median; box limits, upper and lower quartiles; whiskers, 1.5 times the interquartile range.

(b) $ECPA_{cell}$ is significantly negatively correlated with the pathologic stage for patients in 6 out of 19 cancer types that have pathological stage data available (***, $P < 0.001$; **, $P < 0.01$; *, $P < 0.05$). Colon and rectum adenocarcinoma are merged as colorectal carcinoma (CRC) in the analysis.

(c) $ECPA_{cell}$ is significantly associated with the patients' survival times in 8 out of 17 cancer types that have ≥75 cases and ≥25% events. Circle size indicates significance of the correlation; color indicates direction of the correlation. The log-rank tests or Cox proportional hazards model was used in the analysis.

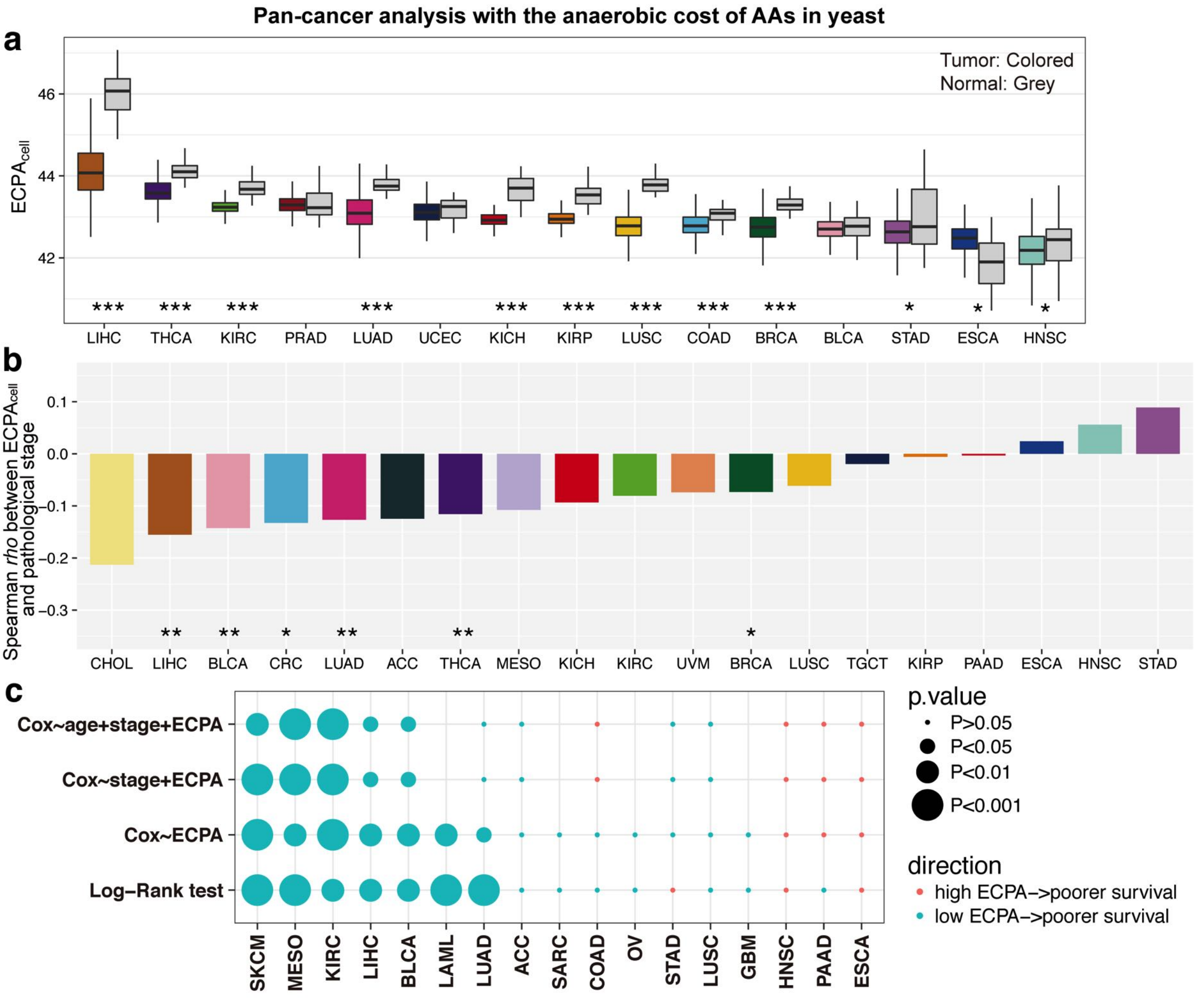

Supplementary Figure 21. Clinically relevant patterns of $ECPA_{cell}$ calculated with anaerobic costs of 20 AAs in yeasts across cancer types.

(a) $ECPA_{cell}$ of tumor samples is significantly lower than that of matched normal tissue samples in 11 out of 15 cancer types for which RNA-Seq data of >10 normal samples were available. Wilcoxon rank-sum tests are performed to compare the difference in $ECPA_{cell}$ between tumor samples and normal tissue samples (***, $P< 0.001$; **, $P < 0.01$; *, $P < 0.05$). Center line, median; box limits, upper and lower quartiles; whiskers, 1.5 times the interquartile range.

(b) $ECPA_{cell}$ is significantly negatively correlated with the pathologic stage for patients in 6 out of 19 cancer types that have pathological stage data available (***, $P < 0.001$; **, $P < 0.01$; *, $P < 0.05$). Colon and rectum adenocarcinoma are merged as colorectal carcinoma (CRC) in the analysis.

(c) $ECPA_{cell}$ is significantly associated with the patients' survival times in 7 out of 17 cancer types that have ≥75 cases and ≥25% events. Circle size indicates significance of the correlation; color indicates direction of the correlation. The log-rank tests or Cox proportional hazards model was used in the analysis.

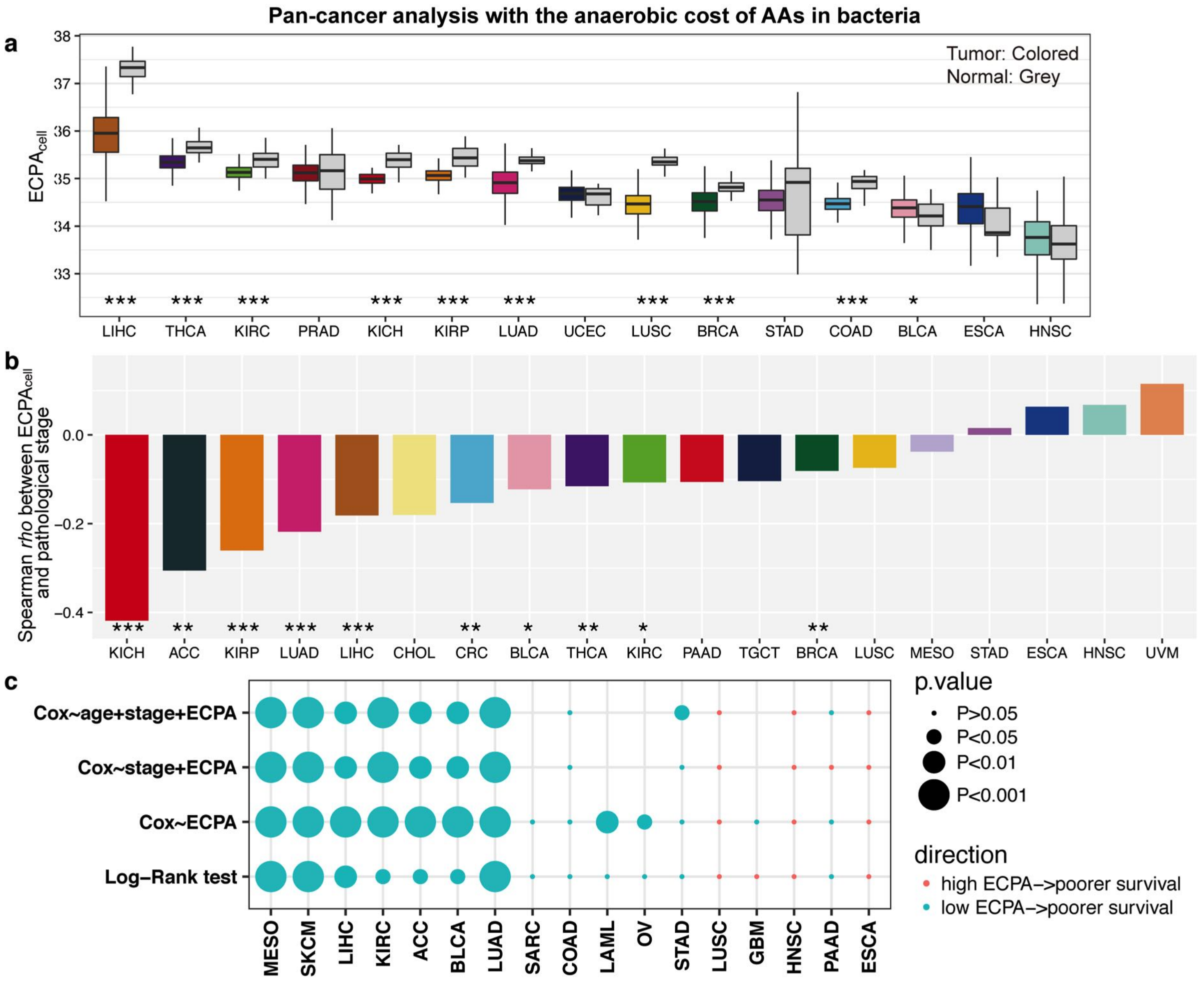

Supplementary Figure 22. Clinically relevant patterns of ECPA$_{cell}$ calculated with anaerobic costs of 20 AAs in bacteria across cancer types.

(a) ECPA$_{cell}$ of tumor samples is significantly lower than that of matched normal tissue samples in 9 out of 15 cancer types for which RNA-Seq data of >10 normal samples were available. Wilcoxon rank-sum tests are performed to compare the difference in ECPA$_{cell}$ between tumor samples and normal tissue samples (***, P< 0.001; **, P < 0.01; *, P < 0.05). Center line, median; box limits, upper and lower quartiles; whiskers, 1.5 times the interquartile range.

(b) ECPA$_{cell}$ is significantly negatively correlated with the pathologic stage for patients in 10 out of 19 cancer types that have pathological stage data available (***, P < 0.001; **, P < 0.01; *, P < 0.05). Colon and rectum adenocarcinoma are merged as colorectal carcinoma (CRC) in the analysis.

(c) ECPA$_{cell}$ is significantly associated with the patients' survival times in 10 out of 17 cancer types that have ≥75 cases and ≥25% events. Circle size indicates significance of the correlation; color indicates direction of the correlation. The log-rank tests or Cox proportional hazards model was used in the analysis.

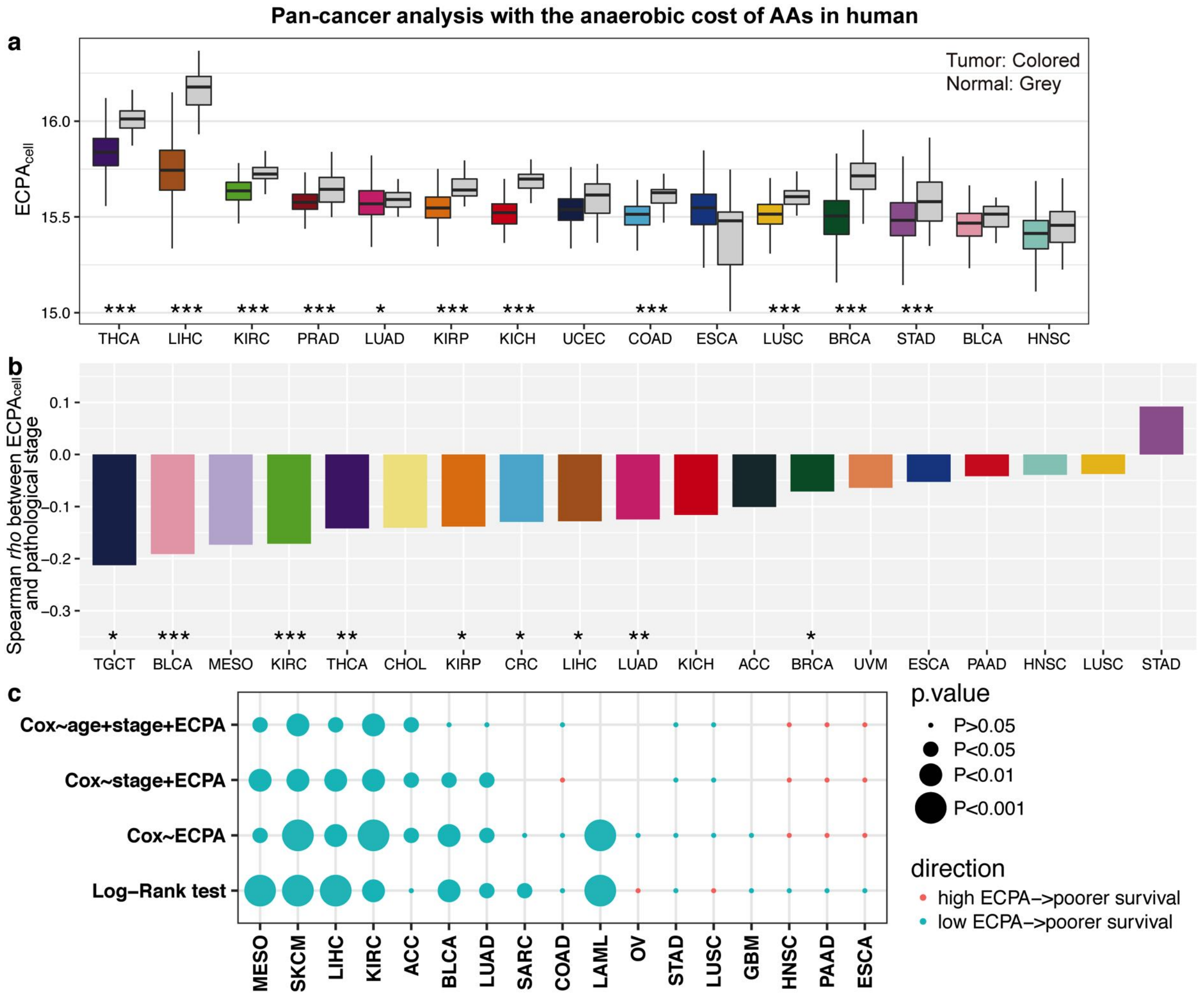

Supplementary Figure 23. Clinically relevant patterns of ECPA$_{cell}$ calculated with anaerobic costs of 11 NEAAs in human across cancer types.

(a) ECPA$_{cell}$ of tumor samples is significantly lower than that of matched normal tissue samples in 11 out of 15 cancer types for which RNA-Seq data of >10 normal samples were available. Wilcoxon rank-sum tests are performed to compare the difference in ECPA$_{cell}$ between tumor samples and normal tissue samples (***, P< 0.001; **, P < 0.01; *, P < 0.05). Center line, median; box limits, upper and lower quartiles; whiskers, 1.5 times the interquartile range.

(b) ECPA$_{cell}$ is significantly negatively correlated with the pathologic stage for patients in 9 out of 19 cancer types that have pathological stage data available (***, P < 0.001; **, P < 0.01; *, P < 0.05). Colon and rectum adenocarcinoma are merged as colorectal carcinoma (CRC) in the analysis.

(c) ECPA$_{cell}$ is significantly associated with the patients' survival times in 9 out of 17 cancer types that have ≥75 cases and ≥25% events. Circle size indicates significance of the correlation; color indicates direction of the correlation. The log-rank tests or Cox proportional hazards model was used in the analysis.

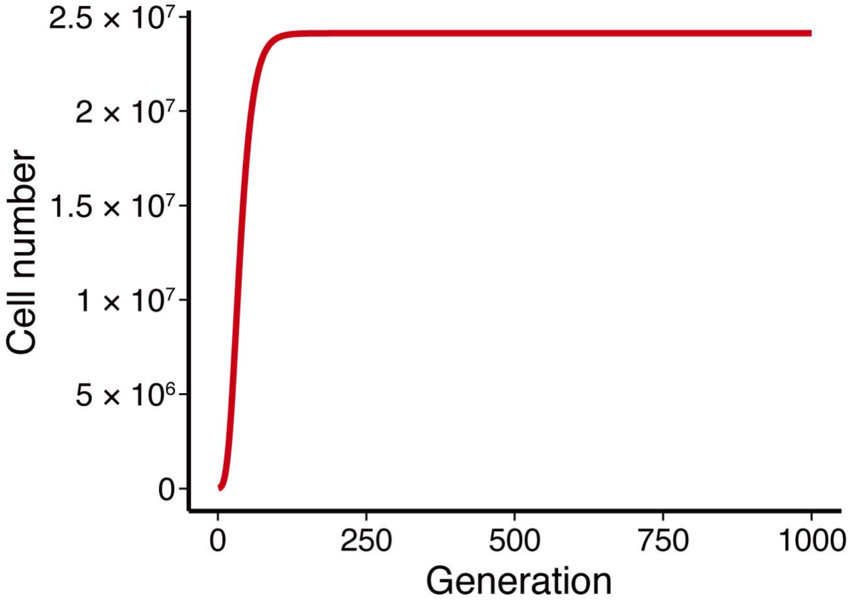

Supplementary Figure 24. The size of the cancer cell population (y-axis) along the generation (x-axis) in the simulations.

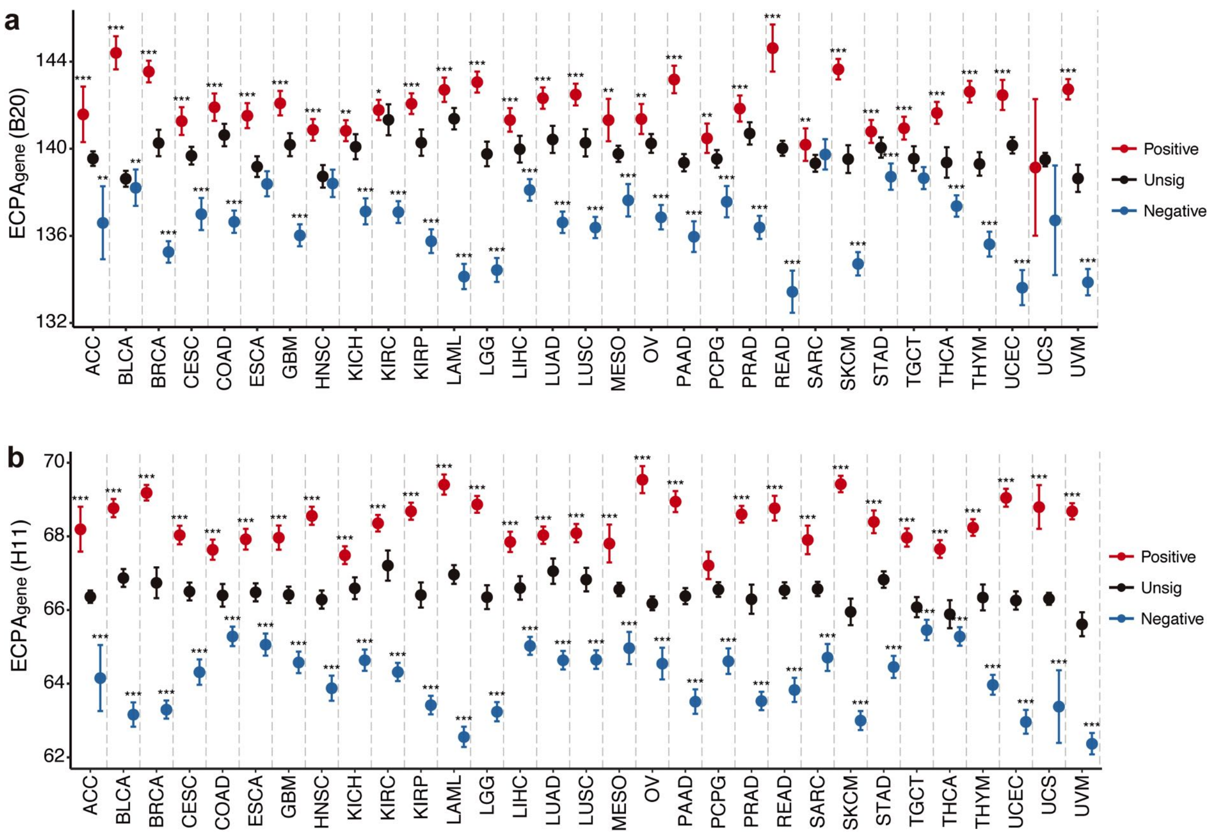

Supplementary Figure 25. Differences in ECPA$_{gene}$ of genes that had expression levels correlated with ECPA$_{cell}$ among samples in 31 TCGA cancer types that have at least 50 samples.

In each cancer type, genes were grouped into three categories: 1) genes that had expression levels positively correlated with ECPA$_{cell}$ (FDR adjusted $P < 0.05$); 2) genes that had expression levels negatively correlated with ECPA$_{cell}$ (FDR adjusted $P < 0.05$); 3) remaining genes that had no significant relationship with ECPA$_{cell}$.

(a) Distribution of ECPA$_{gene}$ of the three categories in each cancer type. ECPA was calculated with cost of AAs in bacteria (B20 metric). Positively correlated genes, negatively correlated genes and the remaining genes were displayed in red, blue, and black, respectively. Wilcoxon rank-sum tests were performed to compare the ECPA$_{gene}$ of positively or negatively correlated genes and that of the remaining genes (*, $P < 0.05$; **, $P < 0.01$; ***, $P < 0.001$).

(b) Distribution of ECPA$_{gene}$ of the three categories in each cancer type. ECPA was calculated with cost of NEAAs in human (H11 metric). Positively correlated genes, negatively correlated genes and the remaining genes were displayed in red, blue, and green, respectively. Wilcoxon rank-sum tests were performed to compare the ECPA$_{gene}$ of positively or negatively correlated genes and that of the remaining genes (*, $P < 0.05$; **, $P < 0.01$; ***, $P < 0.001$). Error bars indicate 95% confidence intervals.

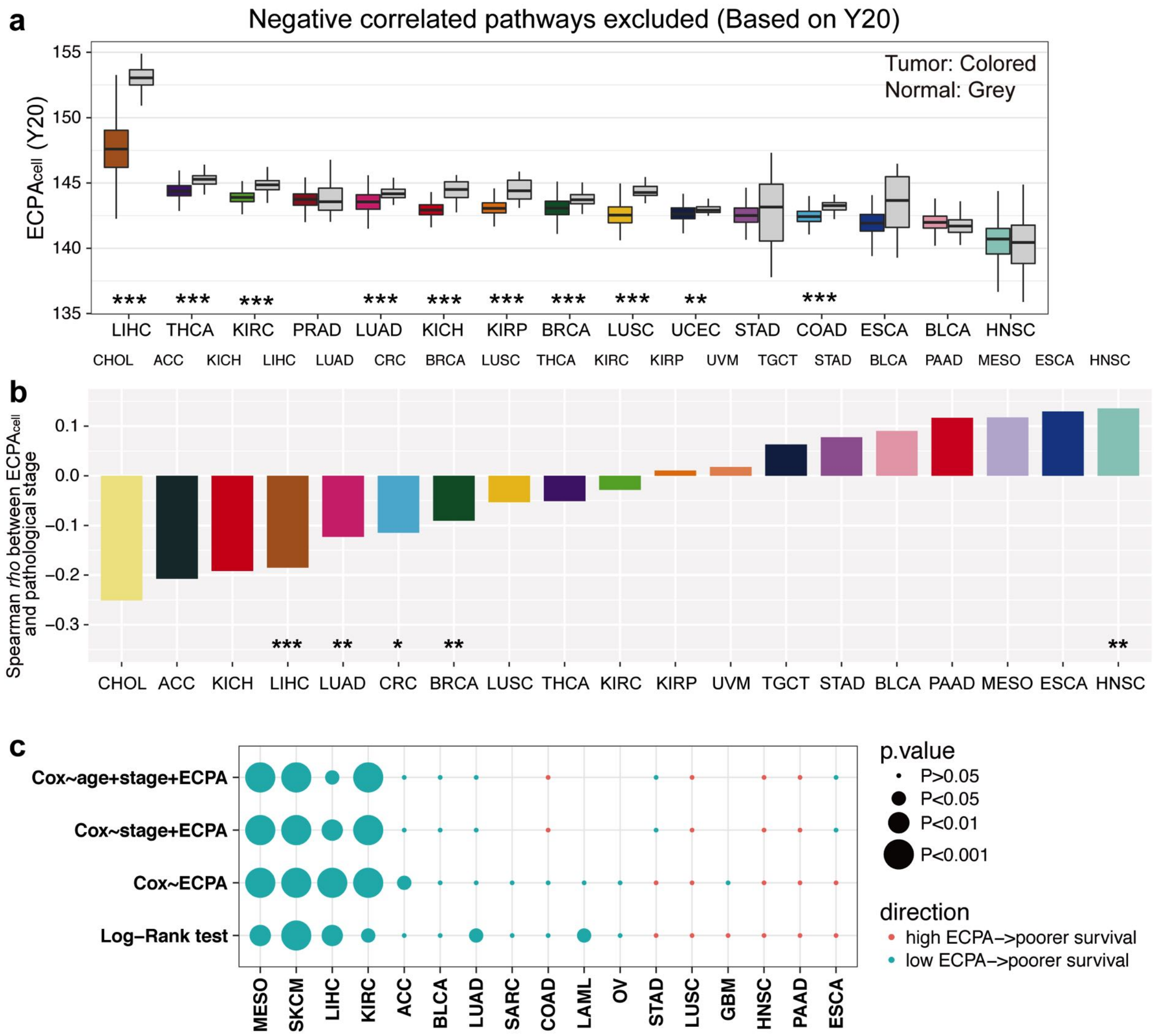

Supplementary Figure 26. Clinically relevant patterns of ECPA$_{cell}$ (Y20) across cancer types after excluding genes that had expression levels negatively correlated with ECPA$_{cell}$ among samples (FDR < 0.05) in at least nine of the 20 cancer types that had lower ECPA$_{cell}$ in tumors or had ECPA$_{cell}$ associated with pathological stage of tumors or patient survival time.

(a) ECPA$_{cell}$ of tumor samples is significantly lower than that of matched normal tissue samples in 13 out of 15 cancer types for which RNA-Seq data of >10 normal samples were available. Wilcoxon rank-sum tests are performed to compared the difference in ECPA$_{cell}$ between tumor samples and normal tissue samples (***, $P < 0.001$; **, $P < 0.01$; *, $P < 0.05$). Center line, median; box limits, upper and lower quartiles; whiskers, 1.5 times the interquartile range.

(b) ECPA$_{cell}$ is significantly negatively correlated with the pathologic stage for patients in 6 out of 19 cancer types that have pathological stage data available (***, $P < 0.001$; **, $P < 0.01$; *, $P < 0.05$). Colon and rectum adenocarcinoma are merged as colorectal carcinoma (CRC) in the analysis.

(c) ECPA$_{cell}$ is significantly associated with the patients' survival times in 8 out of 17 cancer types that have ≥75 cases and ≥25% events. Circle size indicates significance of the correlation; color indicates direction of the correlation. The log-rank tests or Cox proportional hazards model was used in the analysis.

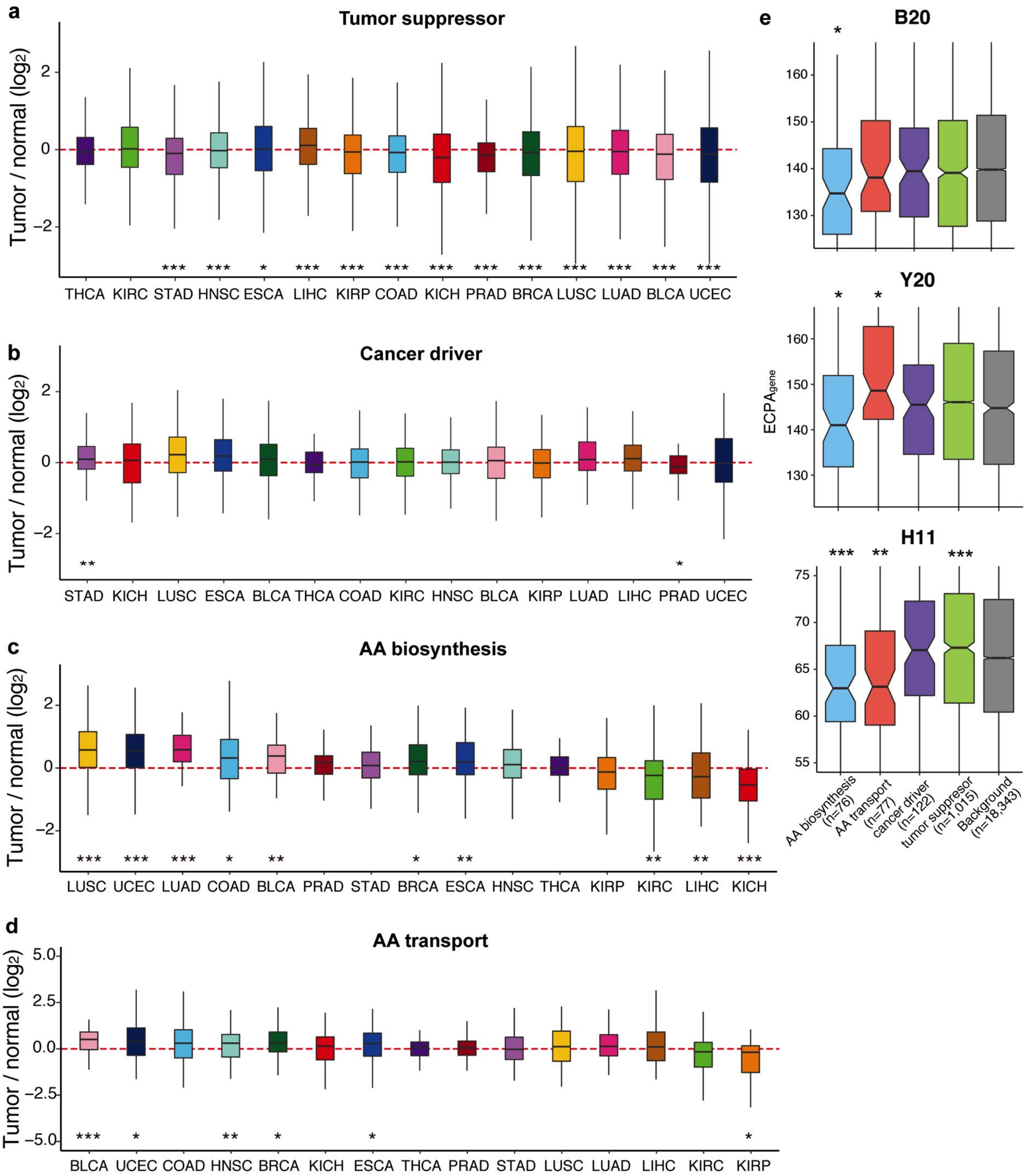

Supplementary Figure 27. Differential expression of gene categories in tumors compared to normal tissues in TCGA data.

(a-d) Log2 fold change in tumors compared to normal tissues for tumor suppressors **(a)**, cancer driver genes **(b)**, and genes related to AA biosynthesis **(c)** or AA transport **(d)**. Only the 15 cancer types with at least 10 normal samples were analyzed. Wilcoxon signed rank tests were used in the comparisons. **(e)** Comparison of $ECPA_{gene}$ of genes in the four categories to that of the remaining genes (background) with Wilcoxon rank-sum test. The number of genes in each category (*n*) was shown in the parenthesis. $ECPA_{gene}$ was calculated with all three cost metrics. Center line, median; box limits, upper and lower quartiles; whiskers, 1.5 times the interquartile range. *, $P < 0.05$; **, $P < 0.01$; ***, $P < 0.001$.

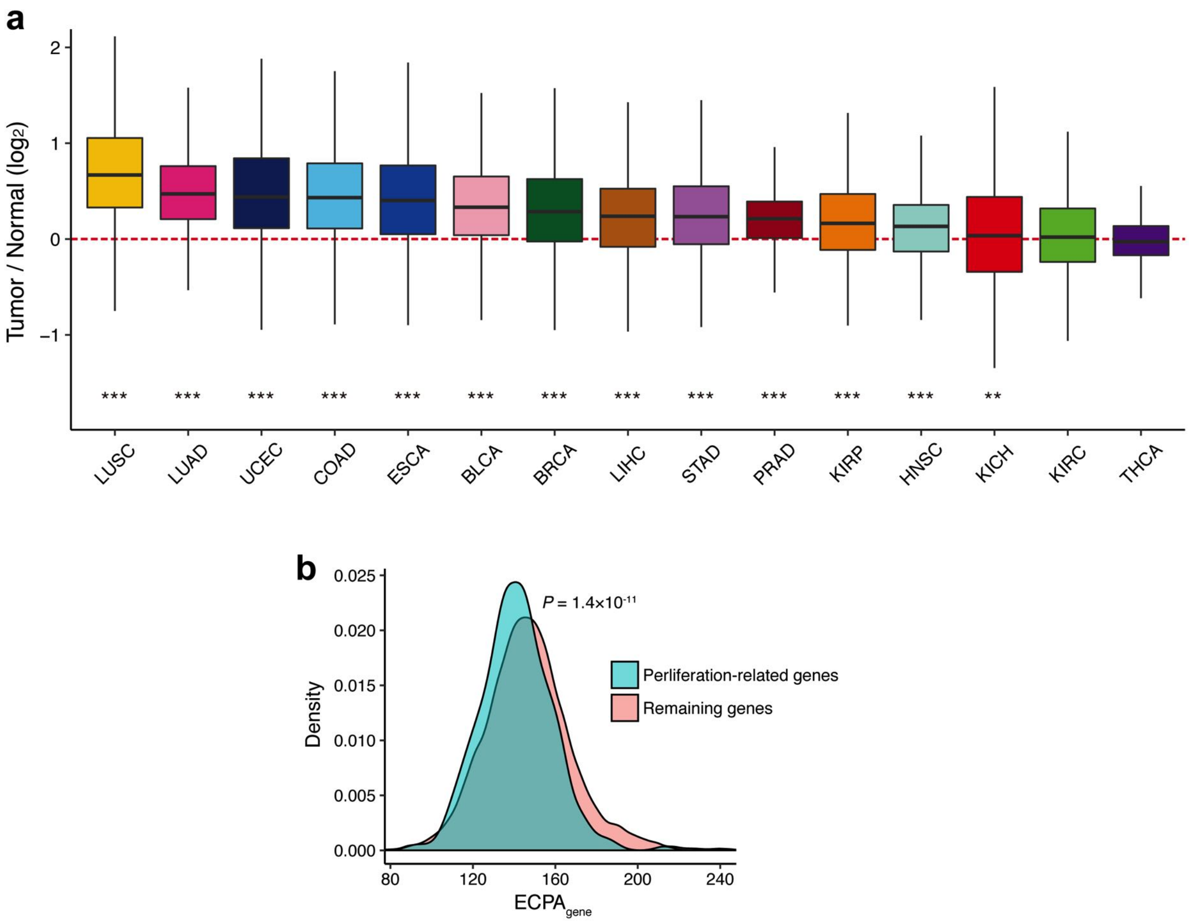

Supplementary Figure 28. Proliferation-related genes were up-regulated in tumors and have lower ECPA$_{gene}$ (Y20).

(a) Box plot of the log_2 ratio of gene expression levels between tumor and normal samples for proliferation-related genes in 15 cancer types for which RNA-Seq data of >10 normal samples were available (Wilcoxon signed rank tests, ***, $P< 0.001$; **, $P < 0.01$; *, $P < 0.05$). Center line, median; box limits, upper and lower quartiles; whiskers, 1.5 times the interquartile range.

(b) The distribution of ECPA$_{gene}$ for proliferation related genes and the remaining genes. Significance of differences was determined with Wilcoxon rank-sum test.

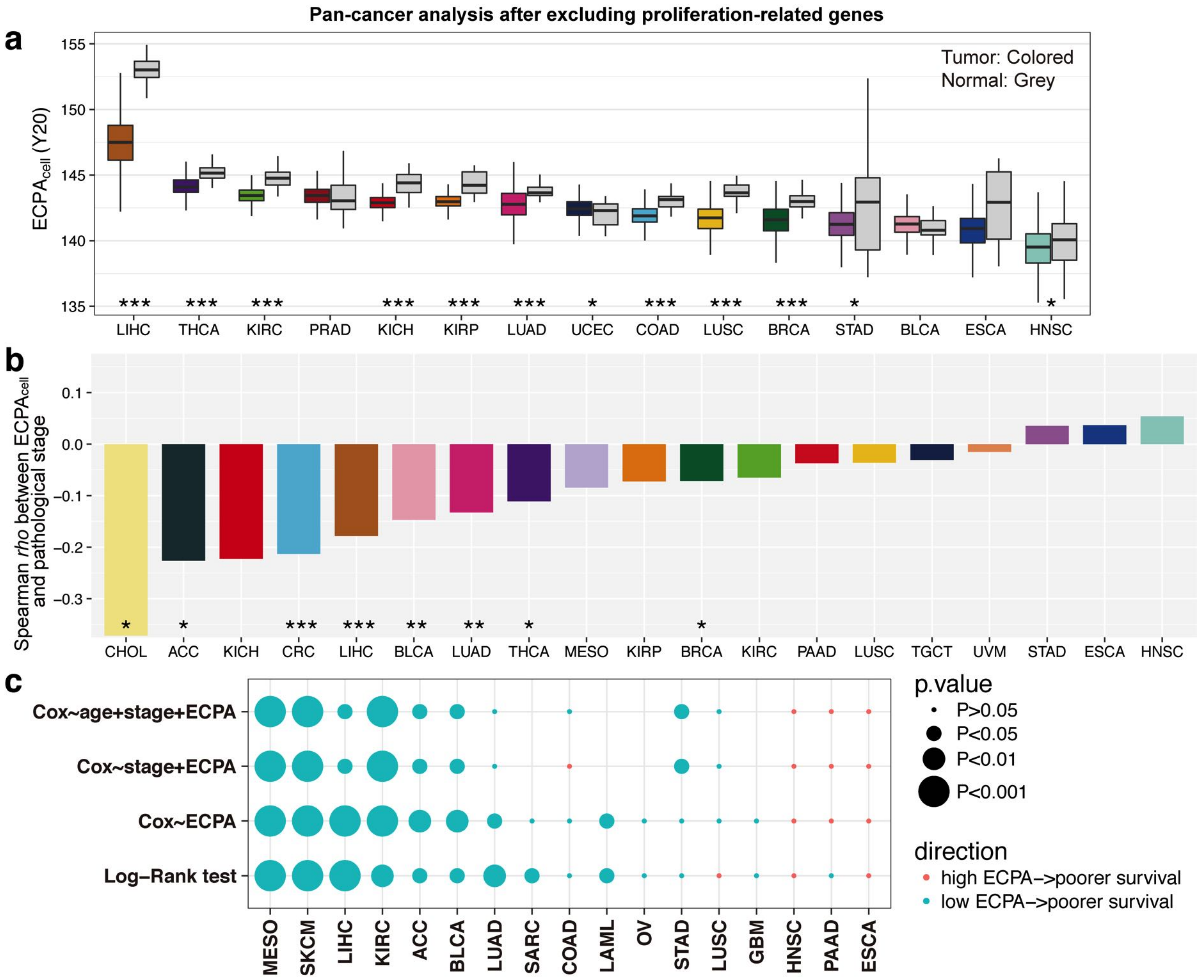

Supplementary Figure 29. Clinically relevant patterns of $ECPA_{cell}$ (Y20) across cancer types after excluding proliferation-related genes.

(a) $ECPA_{cell}$ of tumor samples is significantly lower than that of matched normal tissue samples in 11 out of 15 cancer types for which RNA-Seq data of >10 normal samples were available. Wilcoxon rank-sum tests are performed to compare the difference in $ECPA_{cell}$ between tumor samples and normal tissue samples (***, $P< 0.001$; **, $P < 0.01$; *, $P < 0.05$). Center line, median; box limits, upper and lower quartiles; whiskers, 1.5 times the interquartile range.

(b) $ECPA_{cell}$ is significantly negatively correlated with the pathologic stage for patients in 8 out of 19 cancer types that have pathological stage data available (***, $P < 0.001$; **, $P < 0.01$; *, $P < 0.05$). Colon and rectum adenocarcinoma are merged as colorectal carcinoma (CRC) in the analysis. **(c)** $ECPA_{cell}$ is significantly associated with the patients' survival times in 10 out of 17 cancer types that have ≥75 cases and ≥25% events. Circle size indicates significance of the correlation; color indicates direction of the correlation. The log-rank tests or Cox proportional hazards model was used in the analysis.

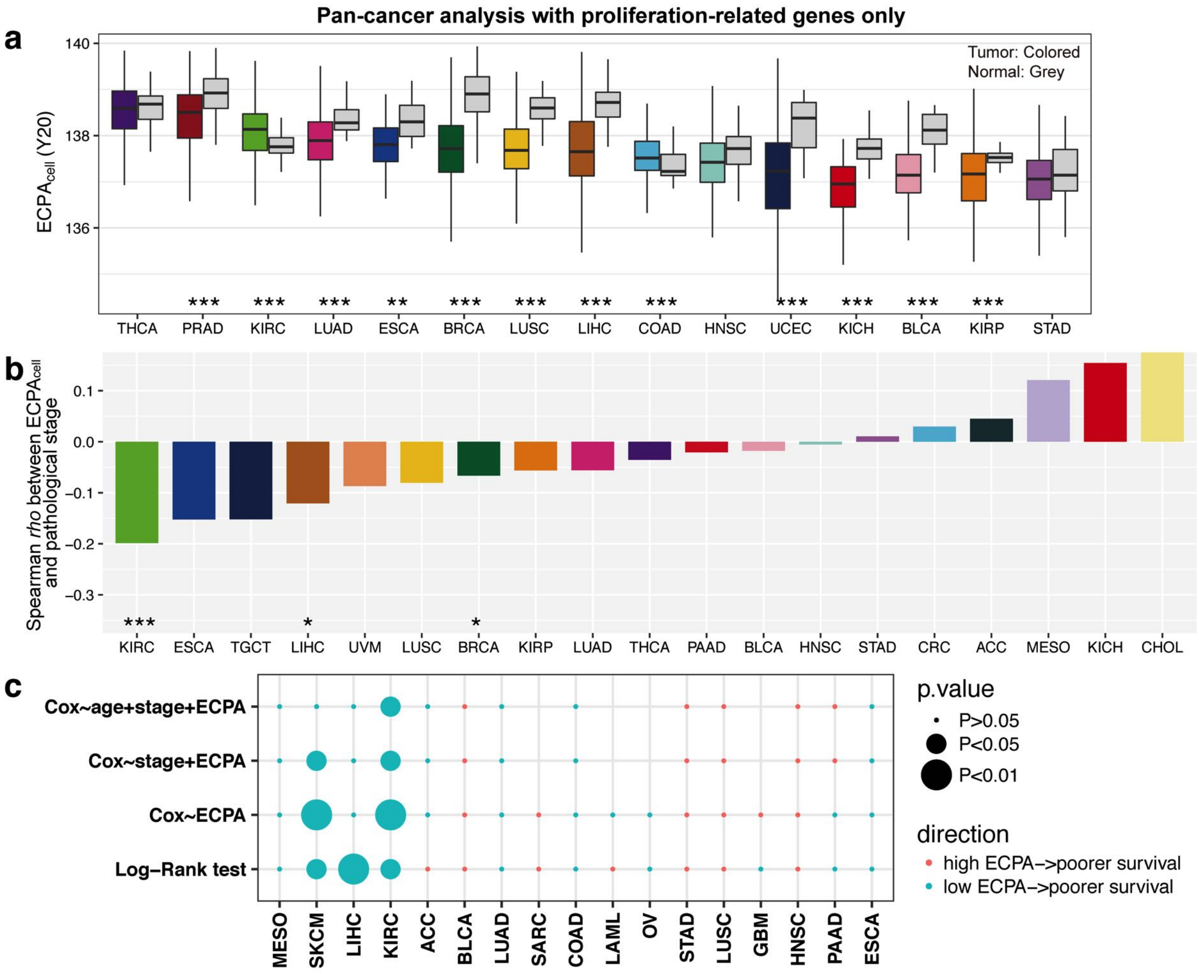

Supplementary Figure 30. Clinically relevant patterns across different cancer types with $ECPA_{cell}$ (Y20) that were calculated with proliferation-related genes only.

(a) Differences in $ECPA_{cell}$ between tumor and normal samples among 15 cancer types for which RNA-Seq data of >10 normal samples were available. Wilcoxon rank-sum tests are performed to determine the significance of differences (***, $P< 0.001$; **, $P < 0.01$; *, $P < 0.05$). Center line, median; box limits, upper and lower quartiles; whiskers, 1.5 times the interquartile range.

(b) $ECPA_{cell}$ is significantly negatively correlated with the pathologic stage for patients in 3 out of 19 cancer types that have pathological stage data available (***, $P < 0.001$; **, $P < 0.01$; *, $P < 0.05$). Colon and rectum adenocarcinoma are merged as colorectal carcinoma (CRC) in the analysis.

(c) $ECPA_{cell}$ is significantly associated with the patients' survival times in 3 out of 17 cancer types that have ≥75 cases and ≥25% events. Circle size indicates significance of the correlation; color indicates direction of the correlation. The log-rank tests or Cox proportional hazards model was used in the analysis.

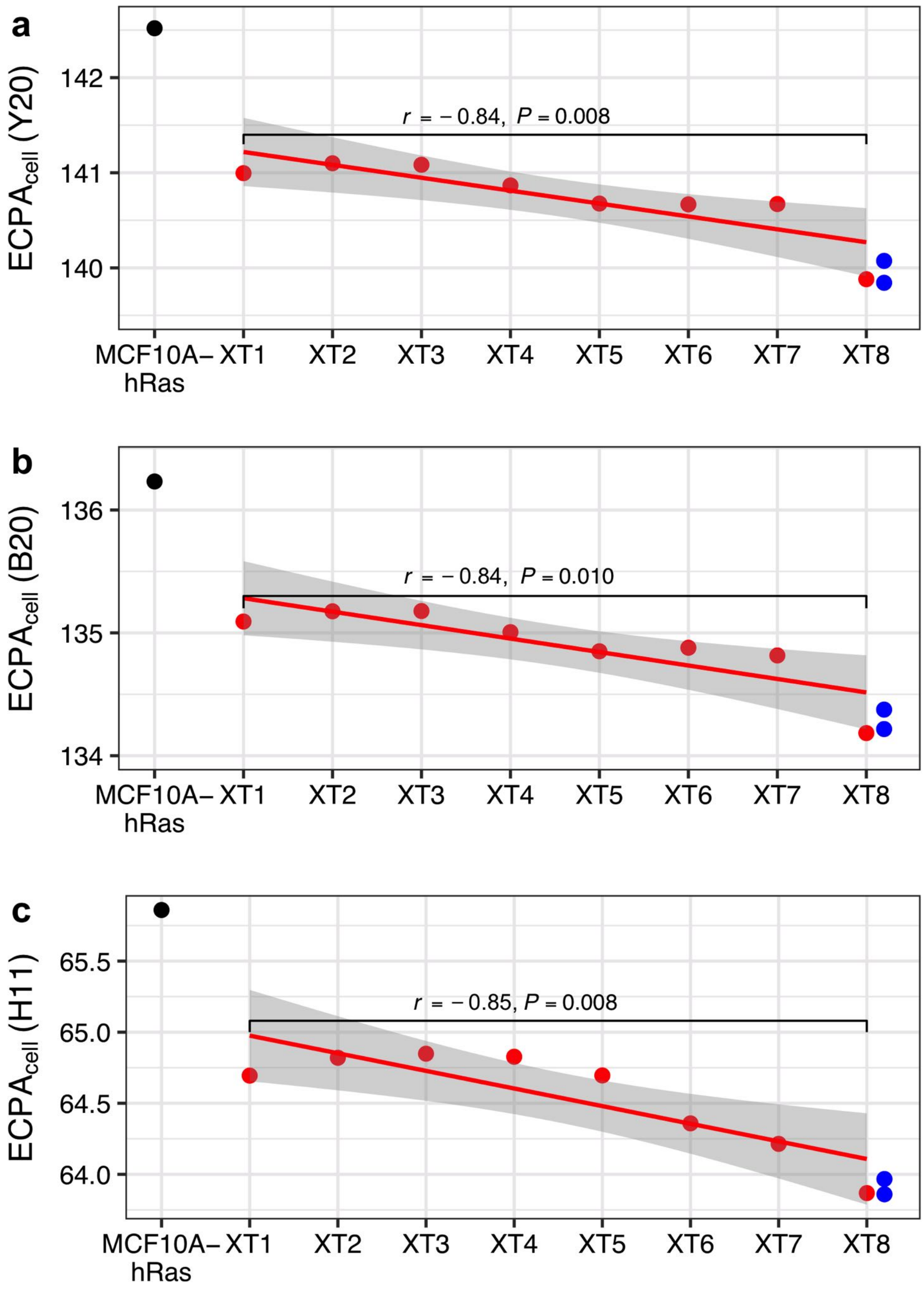

Supplementary Figure 31. The decreasing trend of $ECPA_{cell}$ during the experimental evolution of a xenograft tumor.

$ECPA_{cell}$ was calculated after excluding proliferation-related genes with Y20 **(a)**, B20 **(b)**, and H11**(c)**. The MCF10A-HRAS cells (in black) were xenografted into mice for generations. XT1, XT2, …, XT8 represent the first-stage xenograft tumor, the second-stage, …, the eighth-stage (in red); two metastatic tumors were detected in the mouse carrying XT8 (in blue). *P* values for linear regression of $ECPA_{cell}$ against generation number (XT1 to XT8) are shown.

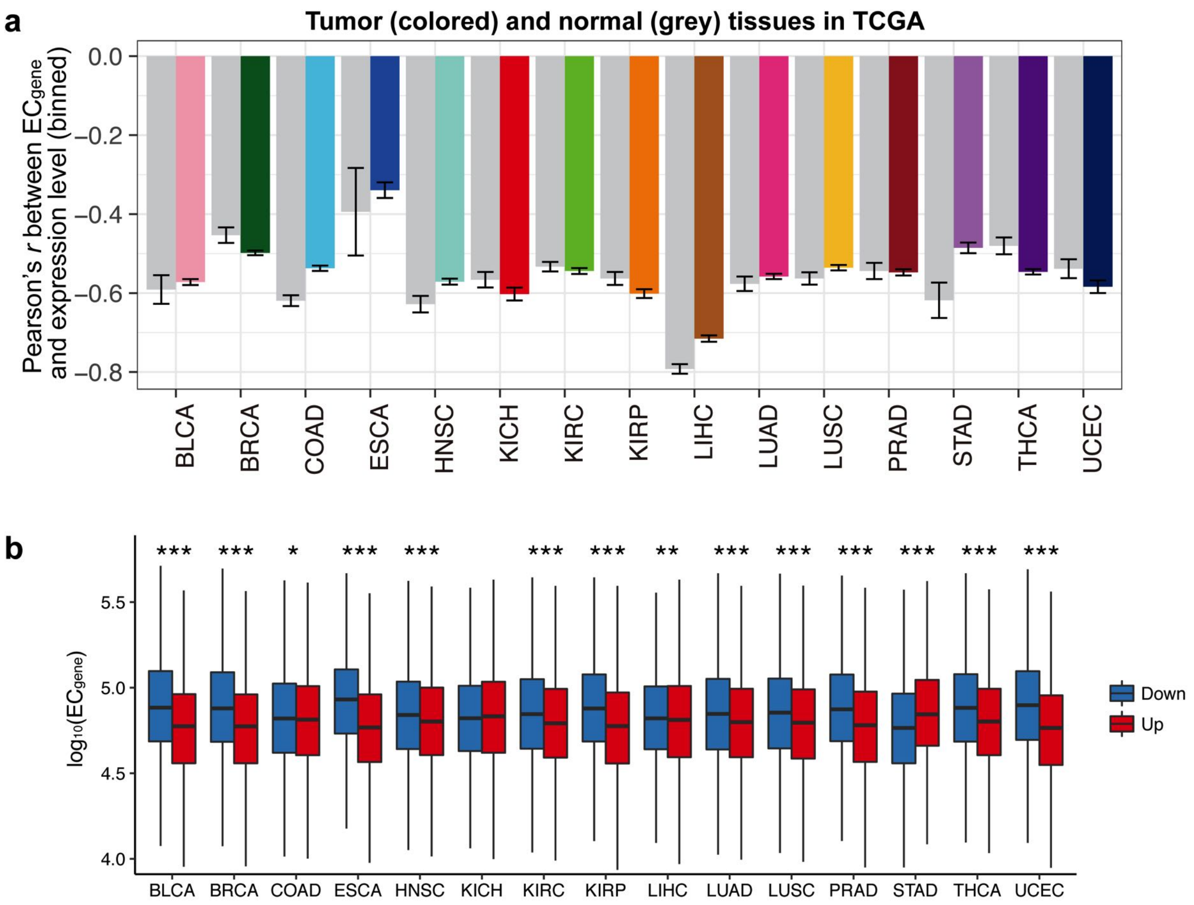

Supplementary Figure 32. Cancer cells prefer expressing genes with lower EC_{gene} (Y20).

(a) Correlations between EC_{gene} and gene expression level across different cancer (colored) and normal tissues (gray) using TCGA RNA-Seq data. For each sample of each cancer type, genes were divided into 100 groups based on their expression levels, and the median expression level and median EC_{gene} in each group were used in the correlation analysis. Error bars indicate the 95% confidence intervals of *r*. **(b)** Differences in EC_{gene} between genes significantly up-regulated in tumors and those significantly down-regulated in tumors across the 15 cancer types for which RNA-Seq data of >10 normal samples were available. Wilcoxon rank-sum tests are performed to determine the significance of differences (***, $P< 0.001$; **, $P < 0.01$; *, $P < 0.05$). Center line, median; box limits, upper and lower quartiles; whiskers, 1.5 times the interquartile range.

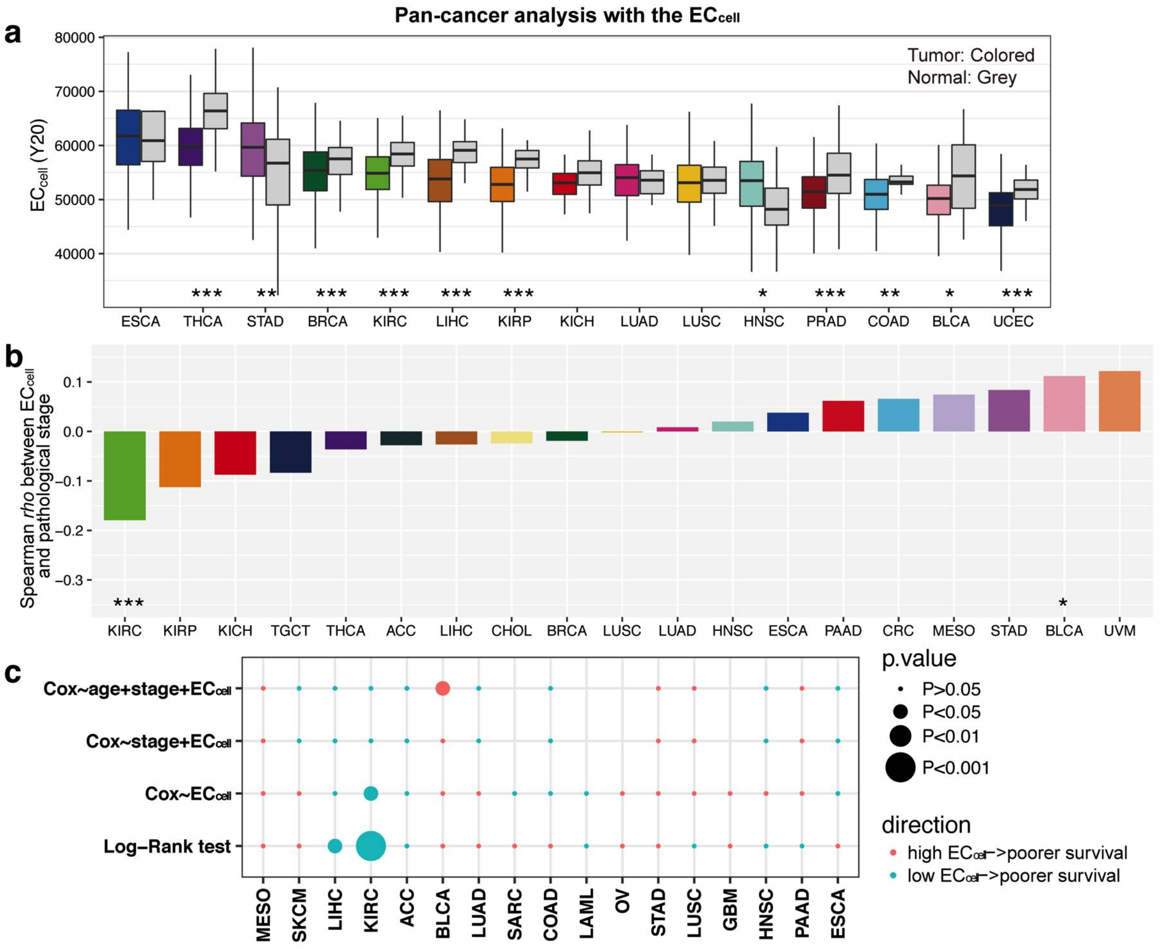

Supplementary Figure 33. Clinically relevant patterns of EC_{cell} (Y20) across cancer types.

(a) Differences in EC_{cell} between tumor and normal samples among 15 cancer types for which RNA-Seq data of >10 normal samples were available. Wilcoxon rank-sum tests are performed to determine the significance of differences (***, P< 0.001; **, P < 0.01; *, P < 0.05). Center line, median; box limits, upper and lower quartiles; whiskers, 1.5 times the interquartile range.

(b) Spearman's *rho* between EC_{cell} and the pathologic stage for patients among 19 cancer types that have pathological stage data available (***, P < 0.001; **, P < 0.01; *, P < 0.05). Colon and rectum adenocarcinoma are merged as colorectal carcinoma (CRC) in the analysis.

(c) EC_{cell} is significantly associated with the patients' survival times in 3 out of 17 cancer types that have ≥75 cases and ≥25% events. Circle size indicates significance of the correlation; color indicates direction of the correlation. The log-rank tests or Cox proportional hazards model was used in the analysis.

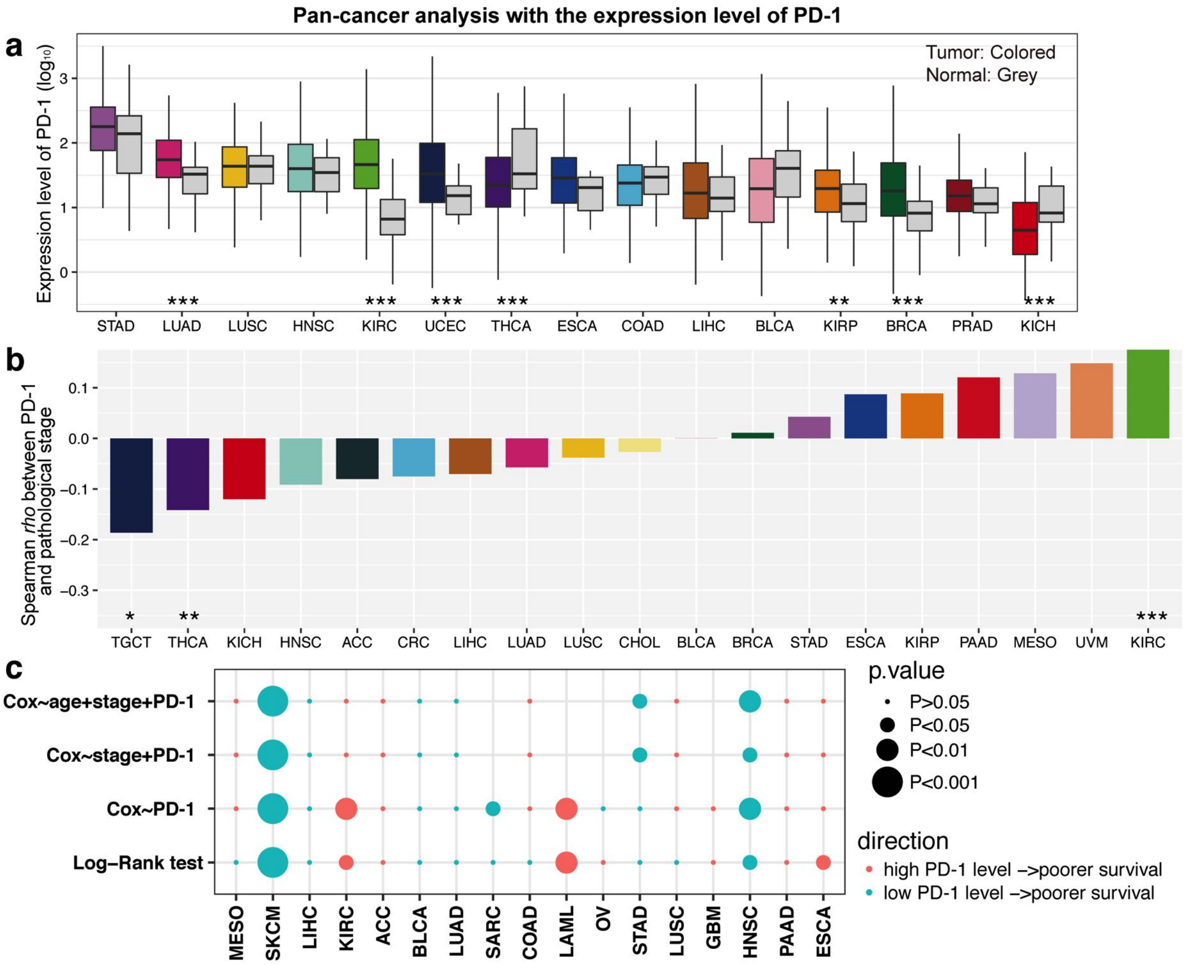

Supplementary Figure 34. Clinically relevant patterns of PD-1 expression levels across cancer types.

(a) Differences in PD-1 expression levels between tumor and normal samples among 15 cancer types for which RNA-Seq data of >10 normal samples were available. Wilcoxon rank-sum tests are performed to determine the significance of the differences (***, $P < 0.001$; **, $P < 0.01$; *, $P < 0.05$). Center line, median; box limits, upper and lower quartiles; whiskers, 1.5 times the interquartile range.

(b) Spearman's *rho* between PD-1 expression levels and the pathologic stage for patients among 19 cancer types that have pathological stage data available (***, $P < 0.001$; **, $P < 0.01$; *, $P < 0.05$). Colon and rectum adenocarcinoma are merged as colorectal carcinoma (CRC) in the analysis.

(c) PD-1 expression levels are significantly associated with the patients' survival times in 7 out of 17 cancer types that have ≥75 cases and ≥25% events. Circle size indicates significance of the correlation; color indicates direction of the correlation. The log-rank tests or Cox proportional hazards model was used in the analysis.

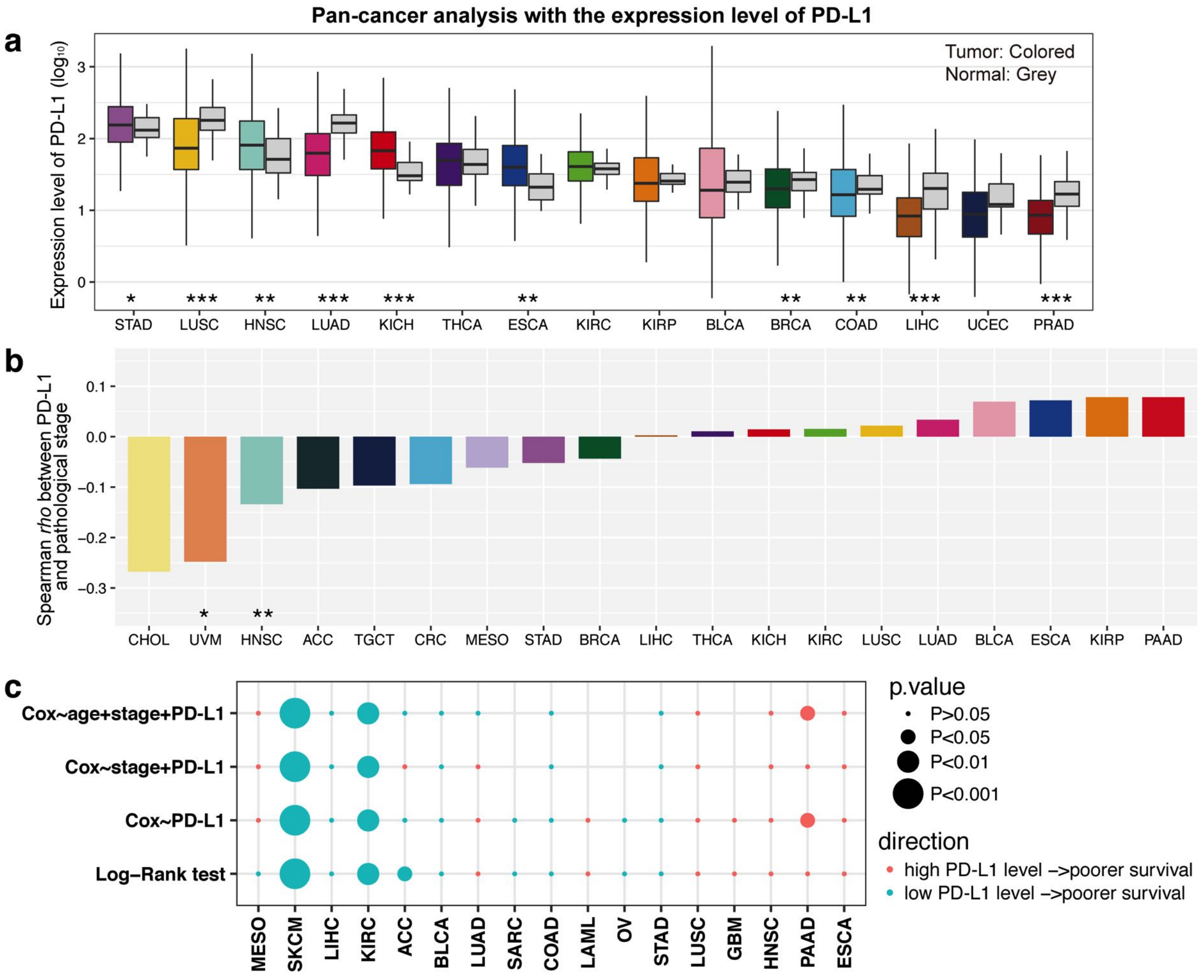

Supplementary Figure 35. Clinically relevant patterns of PD-L1 expression levels across cancer types.

(a) Differences in PD-L1 expression levels between tumor and normal samples among 15 cancer types for which RNA-Seq data of >10 normal samples were available. Wilcoxon rank-sum tests are performed to determine the significance of the differences (***, $P < 0.001$; **, $P < 0.01$; *, $P < 0.05$). Center line, median; box limits, upper and lower quartiles; whiskers, 1.5 times the interquartile range.

(b) Spearman's *rho* between PD-L1 expression levels and the pathologic stage for patients among 19 cancer types that have pathological stage data available (***, $P < 0.001$; **, $P < 0.01$; *, $P < 0.05$). Colon and rectum adenocarcinoma are merged as colorectal carcinoma (CRC) in the analysis.

(c) PD-L1 expression levels are significantly associated with the patients' survival times in 4 out of 17 cancer types that have ≥75 cases and ≥25% events. Circle size indicates significance of the correlation; color indicates direction of the correlation. The log-rank tests or Cox proportional hazards model was used in the analysis.

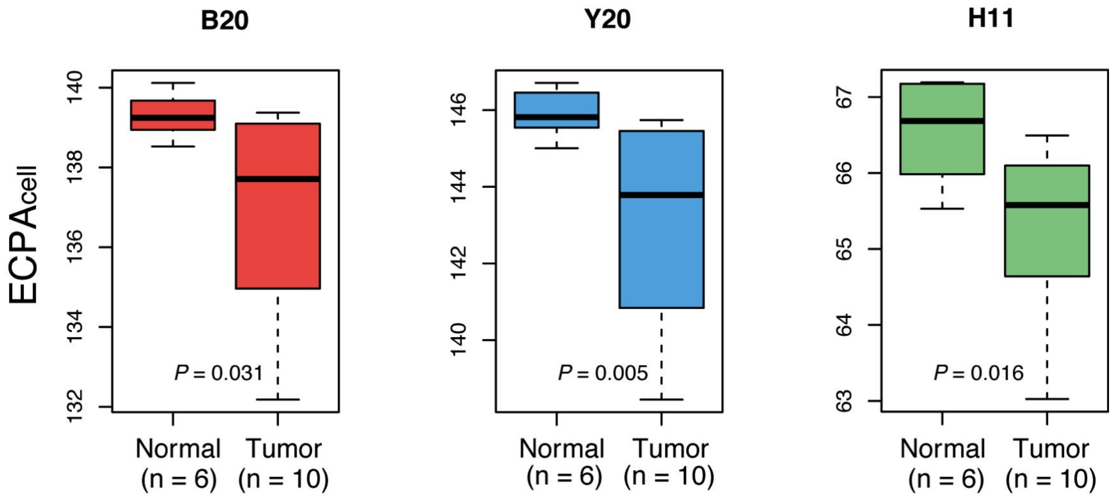

Supplementary Figure 36. The difference in $ECPA_{cell}$ calculated with the ribosome profiling data between Tumor tissues (*n* = 10) and normal tissues (*n*=6) take from four patients (Loayza-Puch et al., 2016).

Wilcoxon rank-sum tests were performed to evaluate the significance of the difference and *P* values are displayed below each panel. Center line, median; box limits, upper and lower quartiles; whiskers, 1.5 times the interquartile range.

Supplementary References

1 Akashi, H. & Gojobori, T. Metabolic efficiency and amino acid composition in the proteomes of Escherichia coli and Bacillus subtilis. *Proceedings of the National Academy of Sciences* **99**, 3695-3700, doi:10.1073/pnas.062526999 (2002).

2 Craig, C. L. & Weber, R. S. Selection costs of amino acid substitutions in ColE1 and ColIa gene clusters harbored by Escherichia coli. *Molecular Biology and Evolution* **15**, 774-776, doi:10.1093/oxfordjournals.molbev.a025981 (1998).

3 Raiford, D. W. *et al.* Do Amino Acid Biosynthetic Costs Constrain Protein Evolution in Saccharomyces cerevisiae? *Journal of Molecular Evolution* **67**, 621-630, doi:10.1007/s00239-008-9162-9 (2008).

4 Wagner, A. Energy Constraints on the Evolution of Gene Expression. *Molecular Biology and Evolution* **22**, 1365-1374, doi:10.1093/molbev/msi126 (2005).

5 Lehninger, A., Nelson, D. & Cox, M. *Lehninger Principles of Biochemistry*. (W. H. Freeman, 2008).

6 van den Heuvel, R. H., Curti, B., Vanoni, M. A. & Mattevi, A. Glutamate synthase: a fascinating pathway from L-glutamine to L-glutamate. *Cellular and molecular life sciences : CMLS* **61**, 669-681, doi:10.1007/s00018-003-3316-0 (2004).

7 Wu, G. & Morris, S. M., Jr. Arginine metabolism: nitric oxide and beyond. *The Biochemical journal* **336 (Pt 1)**, 1-17 (1998).

8 Lepiniec, L., Vidal, J., Chollet, R., Gadal, P. & Crétin, C. Phosphoenolpyruvate carboxylase: structure, regulation and evolution. *Plant Science* **99**, 111-124, doi:10.1016/0168-9452(94)90168-6 (1994).

9 Selhub, J. Homocysteine metabolism. *Annual review of nutrition* **19**, 217-246, doi:10.1146/annurev.nutr.19.1.217 (1999).

10 Krick, T. *et al.* Amino Acid metabolism conflicts with protein diversity. *Mol Biol Evol* **31**, 2905-2912, doi:10.1093/molbev/msu228 (2014).

11 Martini, A. E. V., Miller, M. W. & Martini, A. Amino acid composition of whole cells of different yeasts. *Journal of Agricultural and Food Chemistry* **27**, 982-984, doi:10.1021/jf60225a040 (1979).

12 Okayasu, T., Ikeda, M., Akimoto, K. & Sorimachi, K. The amino acid composition of mammalian and bacterial cells. *Amino Acids* **13**, 379-391, doi:10.1007/BF01372601 (1997).

13 Dereziński, P., Klupczynska, A., Sawicki, W., Pałka, J. A. & Kokot, Z. J. Amino Acid Profiles of Serum and Urine in Search for Prostate Cancer Biomarkers: a Pilot Study. *International Journal of Medical Sciences* **14**, 1-12, doi:10.7150/ijms.15783 (2017).
